# Diversity, Relationships, and Biogeography of the Lambeosaurine Dinosaurs from the European Archipelago, with Description of the New Aralosaurin *Canardia garonnensis*

**DOI:** 10.1371/journal.pone.0069835

**Published:** 2013-07-26

**Authors:** Albert Prieto-Márquez, Fabio M. Dalla Vecchia, Rodrigo Gaete, Àngel Galobart

**Affiliations:** 1 Bayerische Staatssammlung für Paläontologie und Geologie, Munich, Germany; 2 Grup de Recerca del Mesozoic, Institut Català de Paleontologia Miquel Crusafont, Sabadell, Spain; 3 Museu de la Conca Dellà, Isona, Spain; University of Pennsylvania, United States of America

## Abstract

We provide a thorough re-evaluation of the taxonomic diversity, phylogenetic relationships, and historical biogeography of the lambeosaurine hadrosaurids from the European Archipelago. Previously published occurrences of European Lambeosaurinae are reviewed and new specimens collected from upper Maastrichtian strata of the south-central Pyrenees are described. No support is found for the recognition of European saurolophines in the available hadrosaurid materials recovered so far from this area. A new genus and species of basal lambeosaurine, *Canardia garonnensis*, is described on the basis of cranial and appendicular elements collected from upper Maastrichtian strata of southern France. *C. garonnensis* differs from all other hadrosaurids, except *Aralosaurus tuberiferus*, in having maxilla with prominent subrectangular rostrodorsal flange; it differs from *A. tuberiferus* in a few maxillary and prefrontal characters. Together with *A. tuberiferus*, *C. garonnensis* integrates the newly recognized tribe Aralosaurini. Inference of lambeosaurine interrelationships via maximum parsimony analysis indicates that the other three known European lambeosaurines are representatives of two additional subclades (tribes) of these hadrosaurids: Tsintaosaurini (*Pararhabdodon isonensis*) and Lambeosaurini (the *Arenysaurus ardevoli*-*Blasisaurus canudoi* clade). The tribes Aralosaurini, Tsintaosaurini, Lambeosaurini, and Parasaurolophini are formally defined and diagnosed for the first time. Three event-based quantitative methods of ancestral range reconstruction were implemented to infer the historical biogeography of European lambeosaurines: Dispersal-Vicariance Analysis, Bayesian Binary MCMC, and Dispersal-Extinction-Cladogenesis. The results of these analyses, coupled with the absence of pre-Maastrichtian lambeosaurines in the Mesozoic vertebrate fossil record of Europe, favor the hypothesis that aralosaurins and tsintaosaurins were Asian immigrants that reached the Ibero-Armorican island via dispersal events sometime during the Maastrichtian. Less conclusive is the biogeographical history of European lambeosaurins; several scenarios, occurring sometime during the Maastrichtian, are possible, from vicariance leading to the splitting of Asian or North American from European ranges to a dispersal event from North America to the European Archipelago.

## Introduction

Lambeosaurine hadrosaurids represent one of the most morphologically derived clades of ornithopod dinosaurs [1], consisting of *Lambeosaurus lambei* and all taxa more closely related to it than to *Hadrosaurus foulkii*, *Saurolophus osborni*, or *Edmontosaurus regalis* [2]. Lambeosaurines are notorious for the great development of the premaxilla and nasal bones to form hollow supracranial crests, which enclose hypertrophied and caudodorsally migrated nasal passages [3], [4]. The fossils of these herbivores have been found in Eurasia and the Americas, in strata spanning the Santonian through the Maastrichtian [5], [6].

Although the remains of hadrosaurids (sensu [2], i.e., the last common ancestor of *H. foulkii*, *Edmontosaurus regalis*, *Saurolophus osborni*, and *Lambeosaurus lambei*, and all its descendants) are relatively common in the uppermost Cretaceous of Europe [7], [8], most of the material is undiagnostic at generic and specific levels. Thus, our understanding of the European hadrosaurid diversity and their evolutionary relationships remains poor in comparison with that of North America and Asia. Although indeterminate saurolophines (i.e., solid crested/unadorned hadrosaurids, Hadrosaurinae of authors; [1]) have been reported in Europe [9], virtually all named hadrosaurid species in the continent are lambeosaurines and come from upper Maastrichtian outcrops of the Tremp and Arén formations in northeastern Spain. These species are *Pararhabdodon isonensis* Casanovas-Cladellas, Santafé-Llopis, and Isidro-Llorens, 1993 [10] from Lleida Province, and *Arenysaurus ardevoli* Pereda-Suberbiola, Canudo, Cruzado-Caballero, Barco, López-Martínez, Oms, and Ruiz-Omeñaca, 2009 [11] and *Blasisaurus canudoi* Cruzado-Caballero, Pereda-Suberbiola, and Ruiz-Omeñaca, 2010 [12] from Huesca Province.

Other European taxa such as *Telmatosaurus transsylvanicus* [13] from the lower Maastrichtian of Romania and *Tethyshadros insularis* Dalla Vecchia, 2009 [14] from the upper Campanian-lower Maastrichtian of Italy are non-hadrosaurid Hadrosauroidea (sensu [2]).

Here, we describe a new genus and species of lambeosaurine hadrosaurid from the latest Cretaceous Ibero-Armorican island of the European Archipelago, identifying a new tribe of basal Lambeosaurinae. Furthermore, we review the systematics of the European lambeosaurine record, documenting new specimens recovered from upper Maastrichtian strata of the south-central Pyrenees. Finally, we re-evaluate the phylogenetic position of all European taxa recognized so far and infer their historical biogeography using quantitative techniques of ancestral area reconstruction.

### Geological Setting of the Uppermost Cretaceous Lambeosaurine-bearing Strata in the Pyrenean Domain

**Figure 1:**
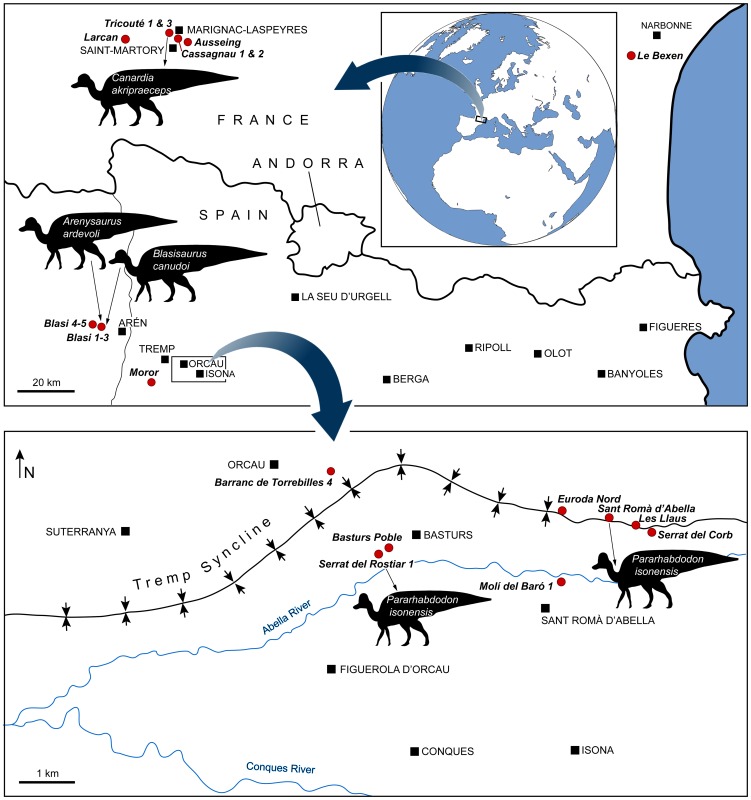
Geographical location of European lambeosaurine localities. Maps constructed, in part, from the data and maps provided by Pereda-Suberbiola et al. [11], Cruzado-Caballero et al. [12], Laurent et al. [16], Laurent [17], Bilotte et al. [18], Riera et al. [24], Brinkmann [76], and Prieto-Márquez et al. [80].

All European lambeosaurine remains come from the Pyrenees (Fig. 1). In the northern Pyrenees, the lambeosaurine-bearing localities occur in the Aurignac Anticline (Tricouté and Cassagnau localities), the Plaigne Anticline (Ausseing locality), and the Latoue/Sepx Syncline (Larcan locality) of the Petit Pyrenees (Haute-Garonne Department, France); purported lambeosaurines have been reported also from the Corbières orientales (Le Bexen locality, Aude Department) [15]. In the south-central Pyrenees, the lambeosaurine-bearing localities occur in the Tremp Syncline of Catalonia and Aragon (Spain).

**Figure 2:**
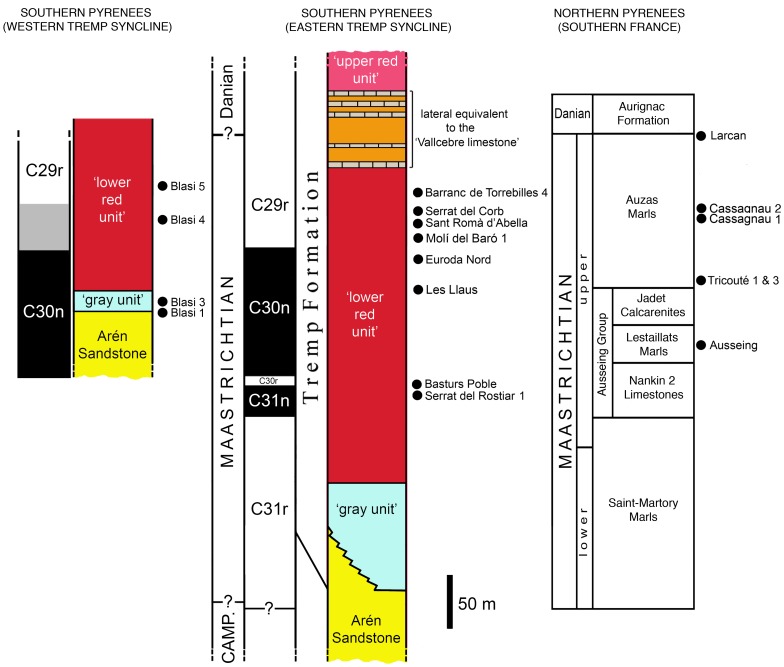
Stratigraphical position of the European lambeosaurine record. The figure shows the stratigraphical and probable chronostratigraphical position of the lambeosaurine and other hadrosaurid-bearing localities discussed in the present study. Stratigraphical columns and stratigraphical positions of the localities are constructed from data in Pereda-Suberbiola et al. [11], Laurent et al. [16], Laurent [17], Riera et al. [24], Riera [29], and Dalla Vecchia et al. [72].

The upper Maastrichtian of the Aurignac Anticline is mostly composed of the ‘Nankin Limestone facies’ and the overlying Auzas Marls Formation [16] (Fig. 2). The Tricouté 1 and 3 localities occur in the basal part of the Auzas Marls Formation (Fig. 2), while the Cassagnau localities are in the middle section of that formation [16], [17]. The Auzas Marls Formation is 100 m thick and shows a regressive trend from transitional (paralic) conditions (lagoon, tidal marsh, etc.) in the lower part to continental ones (alluvial plain) in the upper part. Its late Maastrichtian age is based on the foraminifer content, the age of the underlying units, and the lateral correlations with the ‘blue marls of Saint Loup’ and the Marly limestones of Gensac of the close Larcan area [16], [18]. Fossils in the Tricouté 3 locality were preserved in a marly-sandstone lens rich in plant remains within a thick sandstone bed [17], while the Cassagnau localities are located in the transition zone between the paralic and the limnic deposits [16].

The upper Maastrichtian of the Saint Martory and Plaigne Anticlines is composed of the Ausseing Group (Nankin 2 Limestones, Lestaillats Marls, and Jadet Calcarenites formations, corresponding to the ‘Nankin Limestone facies’ of the close Aurignac [16], [17] and the overlying Auzas Marls Formation (Fig. 2). The Ausseing locality occurs in the middle of the upper Maastrichtian Lestaillats Marls Formation [17] (Fig. 2). This 20 m-thick unit deposited in an brackish estuarine marsh environment [19] and is dated to the early late Maastrichtian, based on the biostratigraphy of the overlying and underlying units (e.g., the transition between the Saint Martory Marls and the Nankin Limestone 2 occurs within the nannoplancton biozone CC25 referred to the upper Maastrichtian [19]).

In the Latoue/Sepx Syncline, the Larcan locality occurs at the top of the uppermost Maastrichtian Marly limestones of Gensac. The lambeosaurine fossils were collected at maximum 1 m below the discontinuity with relatively high iridium concentration marking locally the Cretaceus-Paleogene (K-Pg) boundary [18]. The marly limestones of Gensac are a lateral (seaward, i.e., westward) equivalent of the uppermost part of the Auzas Marls Formation; they deposited in a marine environment and contain Maastrichtian foraminfers (*Lepidorbitoides socialis*, *Orbitoides apiculata, Hellenocyclina beotica, Siderolites calcitrapoides* etc.) and ammonites [18].

The purported lambeosaurine fossils from the Corbières orientales (Le Bexen locality near the town of Fonjoncouse) are from the continental Red marls of Roquelogue that are referred to the upper Maastrichtian by Laurent et al. [15], [17].

The uppermost Cretaceous-Palaeogene transitional to continental deposits in the south-central Pyrenees (Fig. 1) have been subdivided into different lithostratigraphic units by various authors [20]–[22]. Here, we follow the division scheme by [22], where the Tremp Formation (equivalent to the ‘Garumnian’ in Leymerie [23] and the Tremp Group in Cuevas [20] is composed of four informal units. In stratigraphic order, these units are: the ‘grey unit’ (or ‘grey Garumnian’, corresponding to La Posa Formation in Cuevas [20]), the ‘lower red unit’ (or ‘lower red Garumnian’, corresponding to the Conques Formation and the Cretaceous portion of the Talarn Formation in Cuevas [20]), the ‘Vallcebre limestone and laterally equivalent limestone beds’ (equivalent to the Suterranya y Sant Salvador de Tolò Formation in Cuevas [20]), and the ‘upper red unit’ (or ‘upper red Garumnian’, corresponding to the Paleogene portion of the Talarn Formation, the Esplugafreda Formation, and the Claret Formation in Cuevas [20]) (Fig. 2). The ‘grey unit’ was deposited in a transitional environment (lagoon, tidal flat, and coastal marsh) with a few marine intercalations, while the ‘lower red unit’ is mainly composed of floodplain mudstone and fluvial sandstone (point bars, channels, etc.) with intercalations of lacustrine limestone.

In the Tremp Syncline, the Tremp Formation overlies and is partly equivalent laterally to the upper Campanian-Maastrichtian Arén Sandstone [11], [24] (Fig. 2). The sedimentary deposits forming the Arén Sandstone have been variably interpreted as originating in barrier-island complexes [25], [26], beach [27], or deltaic [28] environments. The Tremp Formation-Arén Sandstone boundary is diachronous because those units were deposited during a regressive event in which the sea was retreating to the west. Thus, the boundary is older in the eastern Tremp Syncline (Conca Dellà, Catalonia) and younger in the westernmost Tremp Syncline (Noguera Ribagorzana valley, Aragon) [11], [24] (Fig. 2). In addition, the thickness of both the ‘gray unit’ and ‘lower red unit’ varies from east to west. The ‘gray unit’ is approximately 100 m thick in the easternmost Tremp Syncline, but thins to 35–40 m near Orcau (see Fig. 1) and is only 20 m thick in the western Tremp Syncline (Blasi sites) [11], [29]. The ‘lower red unit’ is approximately 300 m thick in the eastern Tremp Syncline [29].

According to the biostratigraphic and magnetostratigraphic evidence presented in Berástegui and Losantos [30], [31], Riera et al. [24], and Riera [29], the ‘gray unit’ in the eastern Tremp Syncline lays probably at the boundary between the lower and upper Maastrichtian. Given the diachronous deposition of the ‘grey unit’, which took place in a regressive context, it becomes younger westward. Thus, this unit is probably upper Maastrichtian at Moror and certainly is that young at the Blasi sites (northwestern Tremp Syncline) where it is found within the C30n [11]. The ‘lower red unit’ is upper Maastrichtian in the whole Tremp Syncline; the uppermost strata of the unit in the eastern Tremp Syncline fall within the magnetochron C29r and the K-Pg boundary lies within the overlying ‘Vallcebre limestone and laterally equivalent limestone beds’ [24], [29], [32].

Over 45 fossil localities yielded hadrosaurid fossils in the ‘lower red unit’ of the eastern Tremp Syncline [24], [33]. The new specimens here described were found in the Serrat del Rostiar 1, Molí del Baró 1, Serrat del Corb, and Barranc de Torrebilles 4 localities (Fig. 1). Serrat del Rostiar 1 is stratigraphically just below the Basturs Poble locality in the mid-lower part of the ‘lower red unit’ of the Tremp Formation (Fig. 2). Serrat del Rostiar 1 occurs about 150 m above the contact with the underlying Arén Formation, and about 180 m below the limestone beds considered laterally equivalent to the Vallcebre Limestone by Riera et al. [24]. It lies some 30 m above an interval with consistent reverse geomagnetic polarity correlated with magnetochron C31r (V. Riera, pers. comm.). Molí del Baró 1, Serrat del Corb, and Barranc de Torrebilles 4, are higher in the section, in the upper part of the ‘lower red unit’ (Fig. 2) where fluvial sandstone and conglomerate bodies (meander point bar and channel infilling) are common. Their stratigraphical position corresponds roughly with that of Sant Romà d’Abella, Les Llaus, and Euroda Nord sites (also reported below) [24] (Fig. 2). They probably lay within the lower part of the magnetochron C29r according to Riera [29] and Oms et al. [32]. Molí del Baró 1 occurs 220 m above the boundary with the ‘grey unit’, and about 60 m below the limestone beds considered as laterally equivalent to the Vallcebre Limestone by Riera et al. [24]. Barranc de Torrebilles 4 is the highest site stratigraphically, occurring approximately 30 m below the base of the limestone beds considered as laterally equivalent to the Vallcebre Limestone [34]. The charophytes *Peckichara sertulata, Peckichara* ‘with tubercles’, and *Maedleriella* sp. A are reported from a level just below this base and support a very high position in the Maastrichtian [35]. The upper part of the ‘lower red unit’ in the eastern Tremp Syncline is therefore roughly time equivalent to the Lance and Hell Creek Formations of the northern Western Interior Basin of North America.

In the Aragon Region, the Blasi 1–5 localities occur in the western Tremp Syncline, near the town of Areny (also known as Arén) in the Noguera Ribagorzana valley (Huesca province, northeastern Spain; Fig. 1). Blasi 1–3 are actually different horizons contained within a few meters-thick section of a single outcrop [8], which is located at the transition between the Arén and Tremp formations. The Blasi 1 horizon is the topmost layer of the Arén Formation, whereas the Blasi 2 and 3 horizons are at the base of the Tremp Formation in the ‘gray unit’. Blasi 4 and 5 lie higher in the Tremp Formation [21], [29]. Blasi 1–3 occur in the upper part of magnetochron C30n [11] (Fig. 2). Blasi 4 is stratigraphically 70 m above Blasi 3 [21] inside an interval of variable polarity that could correspond to the uppermost part of C30n or the lowermost part of the C29r [11]. Blasi 5 is stratigraphically 100 m above Blasi 3 [21] inside an interval of reverse polarity corresponding to the C29r [11] (Fig. 2). Lambeosaurine remains were collected from Blasi 1, 3–5.

## Results

### Systematic Paleontology

Dinosauria Owen, 1842 [36].

Ornithischia Seeley, 1887 [37].

Ornithopoda Marsh, 1881 [38].

Hadrosauridae Cope, 1870 [39].

Lambeosaurinae Parks, 1923 [40].

### Aralosaurini, New Tribe

urn:lsid:zoobank.org:act:543C13C2-EECE-4B57-A3B8-9BAE6C5A89AB.

#### Definition

The most exclusive clade of lambeosaurine hadrosaurids containing *Aralosaurus tuberiferus* Rozhdestvensky, 1968 [41] and *Canardia garonnensis* n. gen. et sp.

#### Diagnosis

Lambeosaurine hadrosaurids possessing maxilla with rostrodorsal region expanded to form prominent subrectangular flange that rises vertically above rostroventral process.

#### Type genus

*Aralosaurus* Rozhdestvensky, 1968 [41].

### *Canardia* gen. nov

urn:lsid:zoobank.org:act:283535B0-93EF-4E28-B6C8-CEC74B674D8E.

#### Etymology

The generic name is a derivative of “canard”, the French word for “duck”, alluding to the hadrosaurian nature of this animal (hadrosauroids are also informally known as “duck-billed” dinosaurs).

#### Diagnosis

As for the only known species.

### Canardia garonnensis sp. nov

urn:lsid:zoobank.org:act:D42E193F-24A1-4A85-B4AF-811E116572B0.

Figs. 3–10, Tables 1 and 2.

**Figure 3:**
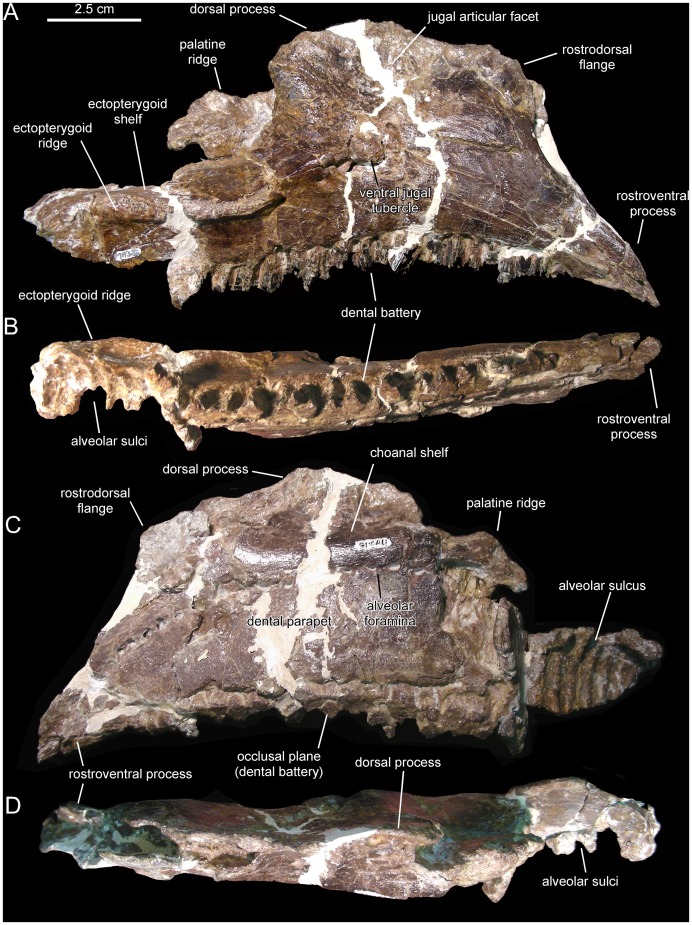
*Canardia garonnensis*, MDE-Ma3–16 (holotype), right maxilla. Maxilla in lateral (A), ventral (B), medial (C), and dorsal (D) views.

**Figure 4:**
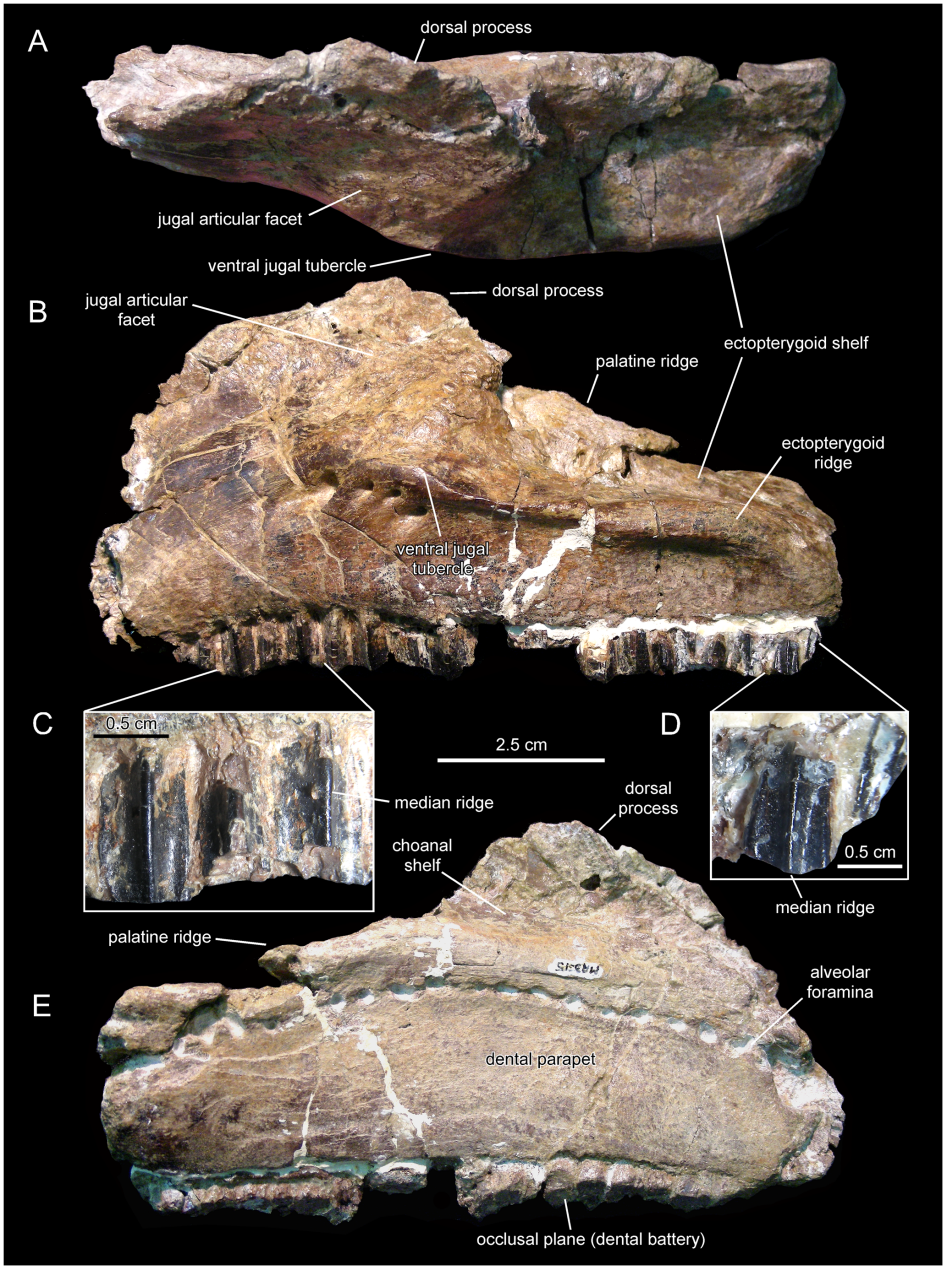
*Canardia garonnensis*, MDE-Ma3–15, left maxilla. Maxilla in dorsal (A), lateral (B), and medial (E) views. Details of the lingual side of tooth crowns are presented in C and D.

**Figure 5:**
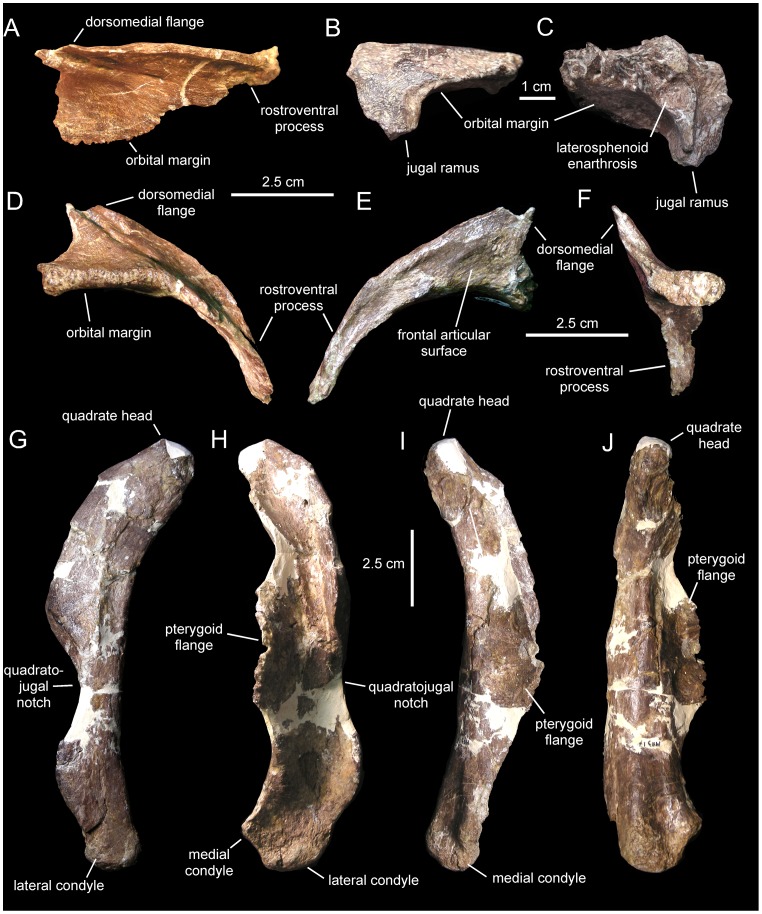
*Canardia garonnensis*, selected cranial elements. A. Right prefrontal (MDE-Ma3–18) in dorsal view. B. Right postorbital (MDE-Ma3–29) in lateral view. C. Medial view of same. D. Lateral view of the prefrontal MDE-Ma3–18. E. Medial view of same. F. Caudal view of same. G. Left quadrate (MDE-Ma3–17) in lateral view. H. Rostral view of same. I. Medial view of same. J. Caudal view of same.

**Figure 6:**
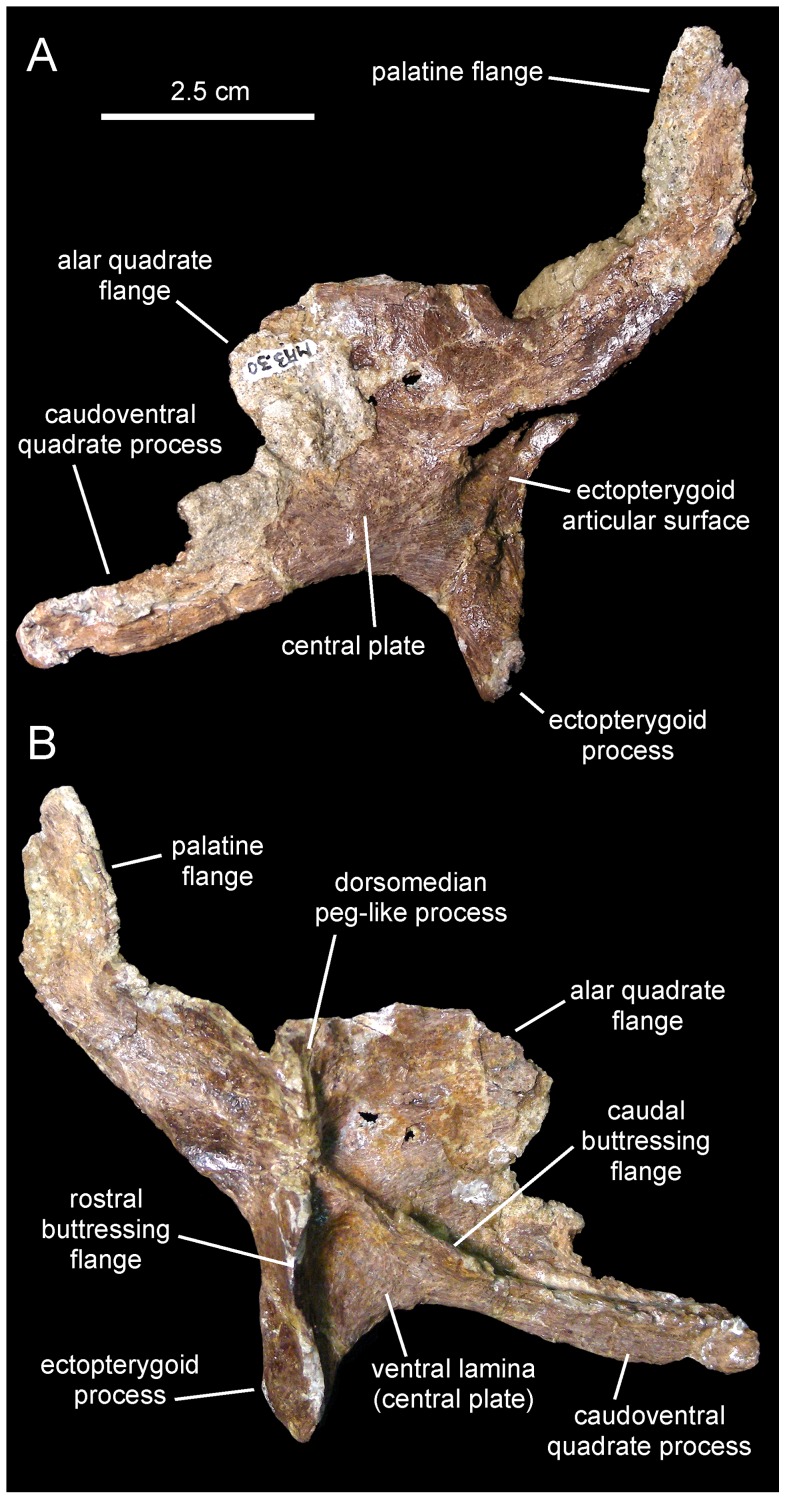
*Canardia garonnensis*, MDE-Ma3–30, right pterygoid. Pterygoid in lateral (A) and medial (B) views.

**Figure 7:**
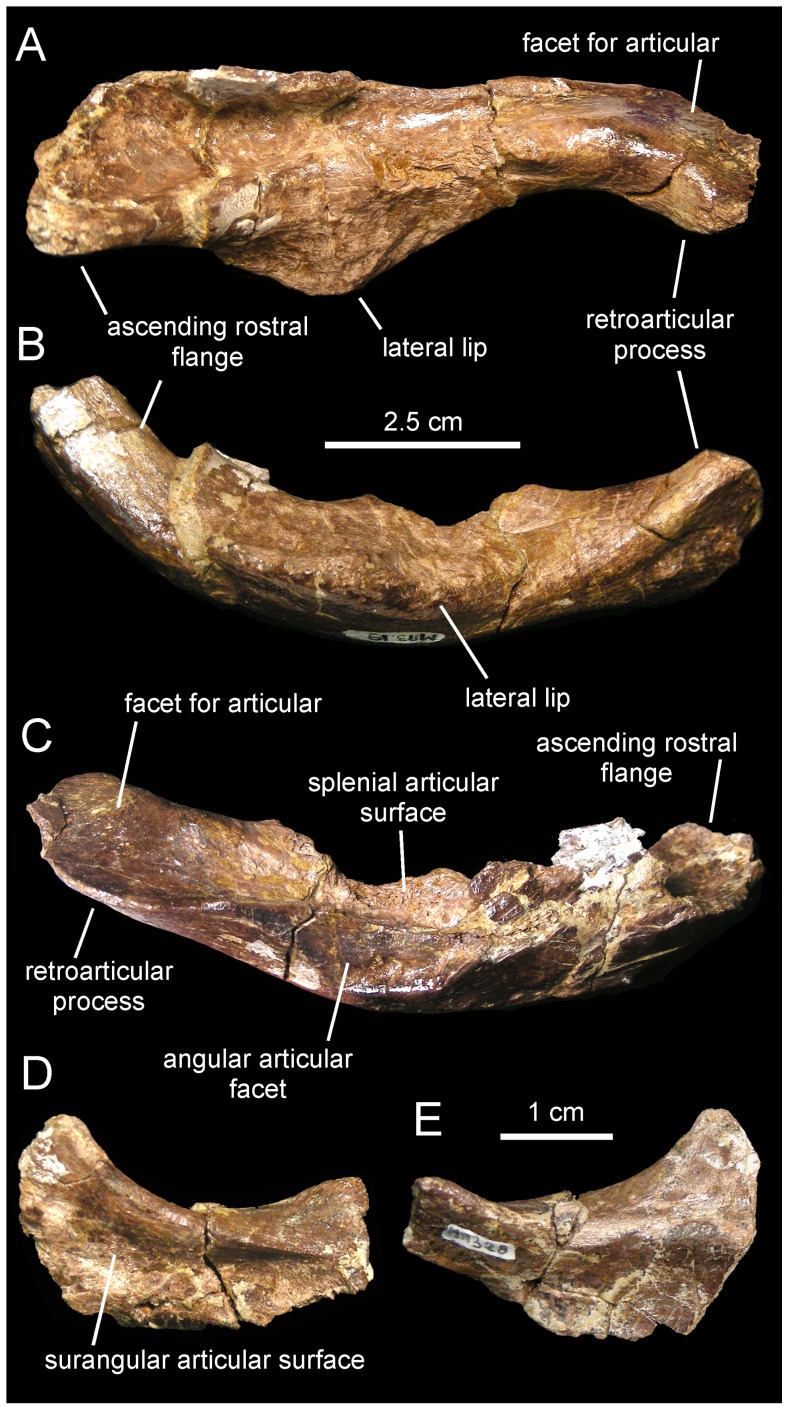
*Canardia garonnensis*, selected mandibular elements. A. Left surangular (MDE-Ma3–19) in dorsal view. B. Lateral view of same. C. Medial view of same. D. Right articular (MDE-Ma3–28) in lateral view. E. Medial view of same.

**Figure 8:**
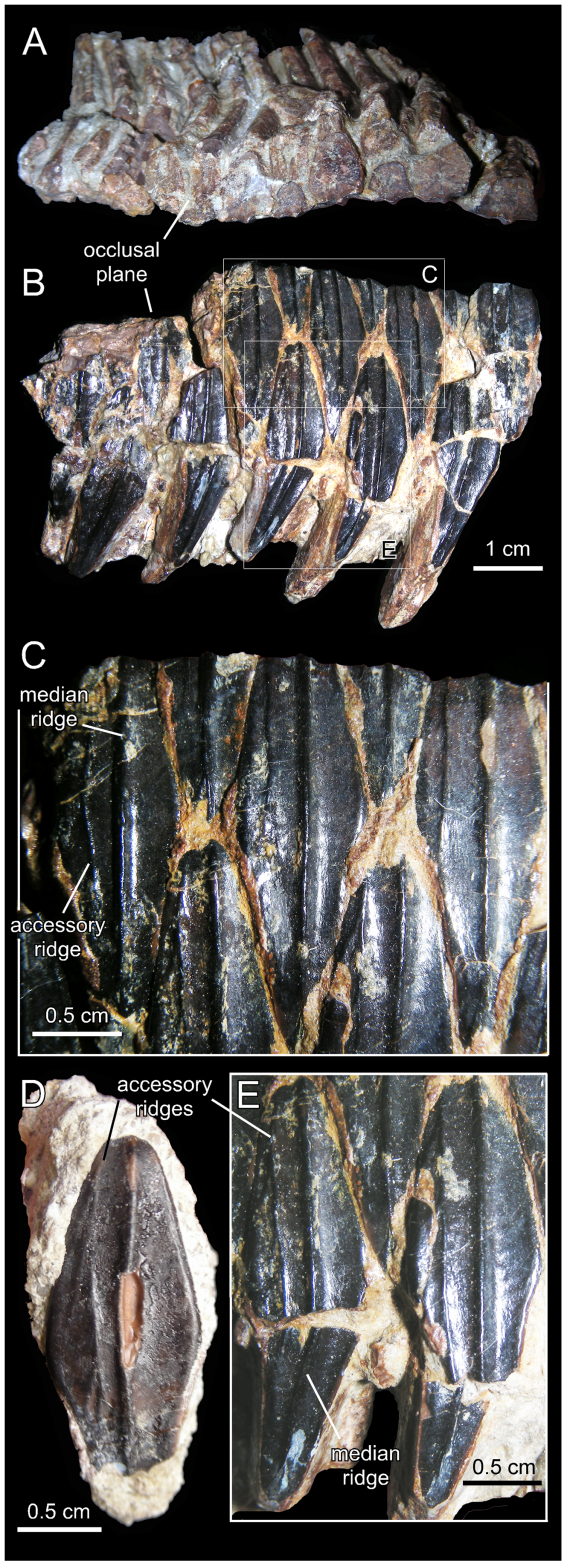
*Canardia garonnensis*, dentary dentition. A. Partial dentary dental battery (MDE-Ma3–26) in occlusal view. B. Lingual view of same. C. Dentary tooth crowns of MDE-Ma3–26 in lingual view. D. Isolated dentary tooth crown (MDE-Ma3–25) in lingual view. E. Dentary tooth crowns of MDE-Ma3–26 in lingual view.

**Figure 9:**
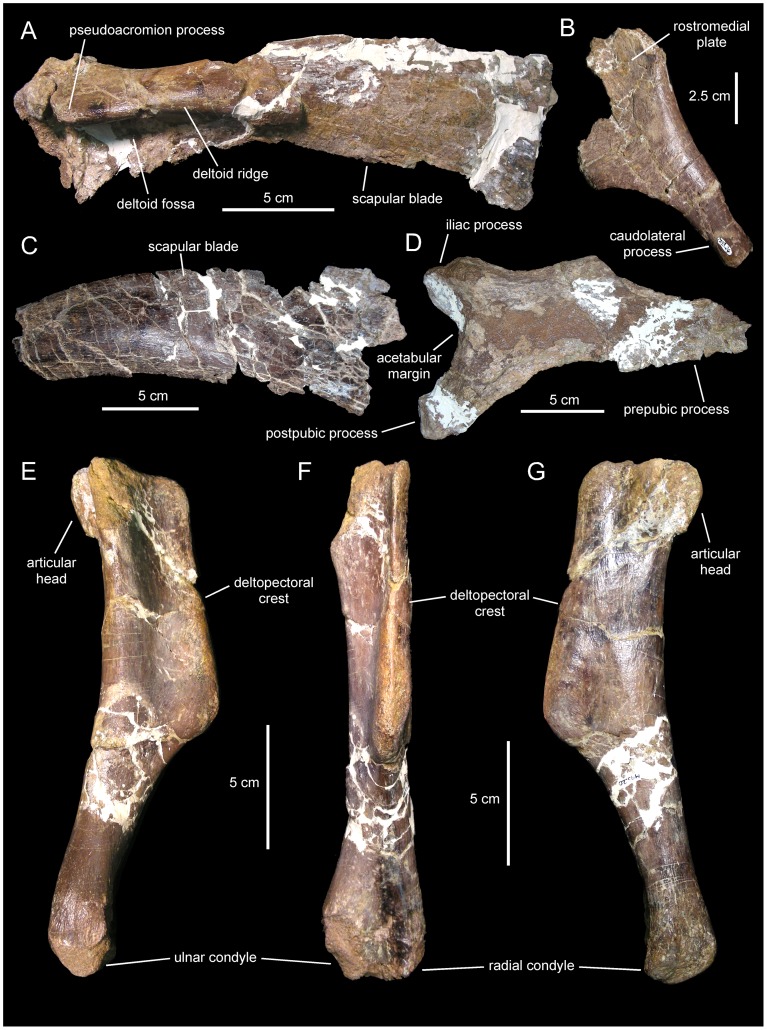
*Canardia garonnensis*, selected appendicular elements. A. Left scapula (MDE-Ma3–21) in lateral view. B. Left sternal plate (MDE-Ma3–24) in ventrolateral view. C. Distal blade of left scapula (MDE-Ma3–12) in lateral view. D. Right pubis (MDE-Ma3–23) in lateral view. E. Left humerus (MDE-Ma3–20) in medial view. F. Rostral view of same. G. Lateral view of same.

**Figure 10:**
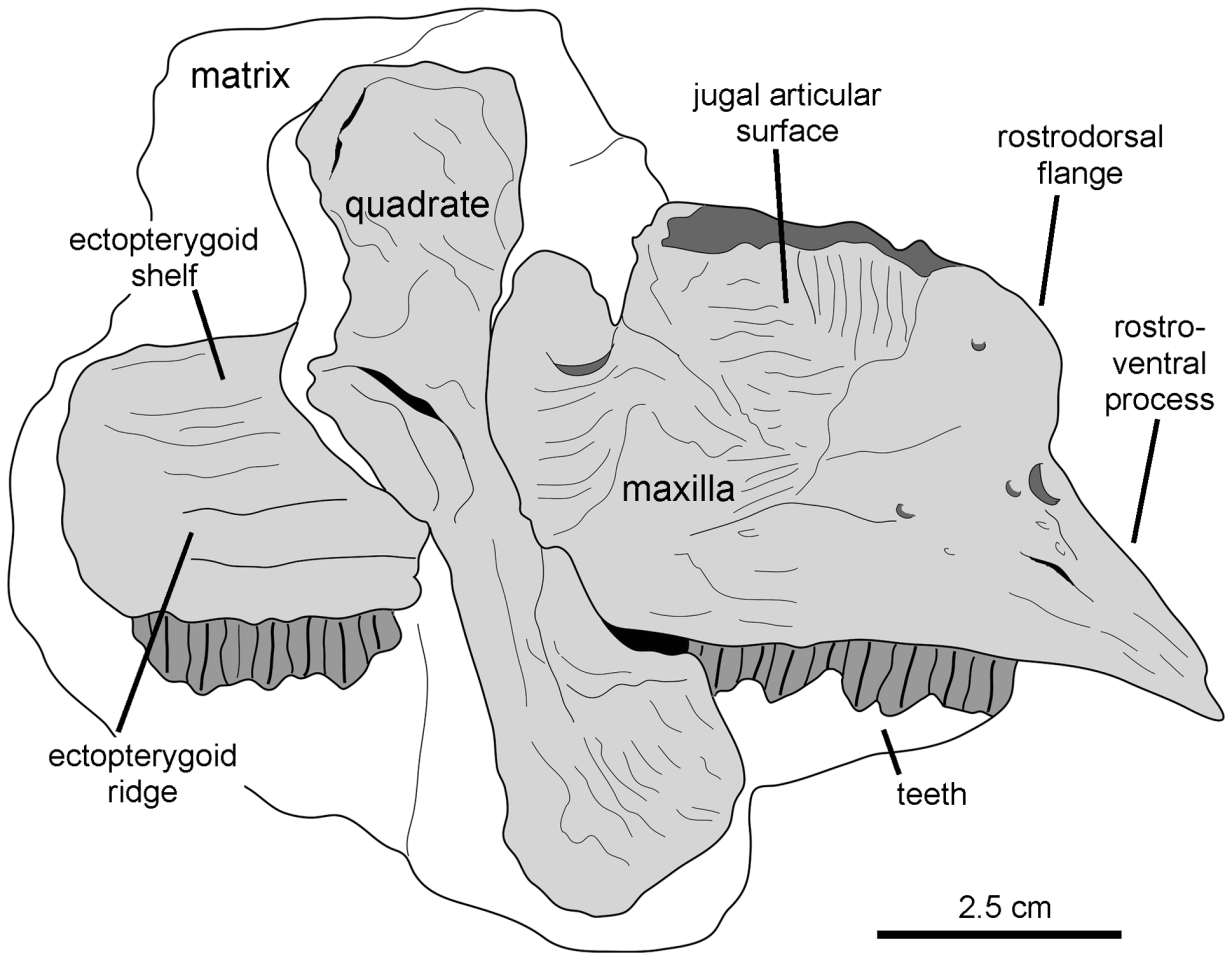
*Canardia garonnensis*, the Larcan specimen. Right maxilla and quadrate of REP-LCR-k6-001. Drawn from Bilotte et al. ([18]: fig. 3).

**Table 1:** Selected cranial measurements (in mm) of *Canardia garonnensis*.

Element	Measurement
Maxilla (MDE-Ma3–15), total length (incomplete specimen)	124
Maxilla (MDE-Ma3–15), length of ectopterygoid shelf	69
Maxilla (MDE-Ma3–15), height from alveolar margin to highest point of dorsal process (incomplete)	59
Maxilla (MDE-Ma3–16), total length	169
Maxilla (MDE-Ma3–16), length of ectopterygoid shelf	70
Maxilla (MDE-Ma3–16), height from alveolar margin to highest point of dorsal process (incomplete)	56
Prefrontal (MDE-Ma3–18), length from rostroventral end to caudodorsal end of dorsomedial flange	71
Prefrontal (MDE-Ma3–18), length of horizontal orbital margin rostral process	30
Prefrontal (MDE-Ma3–18), height from highest point of dorsomedial flange to ventral edge of horizontal orbital margin at mid-length	26
Quadrate (MDE-Ma3–17), total length	129
Quadrate (MDE-Ma3–17), length of quadratojugal notch	44
Quadrate (MDE-Ma3–17), width of jugal flange	26
Pterygoid (MDE-Ma3–30), length of caudoventral buttressing flange including caudoventral quadrate process	52
Pterygoid (MDE-Ma3–30), length of rostral buttressing flange including ectopterygoid process	29
Pterygoid (MDE-Ma3–30), length from distal end of palatine flange to end of caudoventral quadrate process	99
Surangular (MDE-Ma3–19), total length (incomplete asceding rostral process)	76
Surangular (MDE-Ma3–19), máximum mediolateral width at level of lateral lip	27
Articular (MDE-Ma3–28), total length	30
Articular (MDE-Ma3–28), máximum width of dorsal margin	8

**Table 2:** Selected appendicular measurements (in mm) of *Canardia garonnensis*.

Element	Measurement
Scapula (MDE-Ma3–21), length from dorsal margin of coracoid facet to dorsodistal corner of scapular blade	218
Scapula (MDE-Ma3–21), maximum width of proximal constriction	40
Scapula (MDE-Ma3–21), maximum width of distal end of blade	72
Scapula (MDE-Ma3–12), length of preserved scapular blade	196
Scapula (MDE-Ma3–12), maximum width of distal end of blade (incomplete)	73
Scapula (MDE-Ma3–12), maximum width of proximal constriction (incomplete)	39
Sternal plate (MDE-Ma3–24), maximum longitudinal length (incomplete rostromedial expansion and caudoventral process)	125
Humerus (MDA-Ma3–20), total length from articular head to ulnar condyle	194
Humerus (MDA-Ma3–20), máximum width of deltopectoral crest	42
Pubis (MDA-Ma3–20), máximum mediolateral width accross distal condyles (eroded ulnar condyle)	40
Pubis (MDA-Ma3–23), craniocaudal length from iliac process to cranial end of incomplete prepubic process	201
Pubis (MDA-Ma3–23),height of acetabular margin	76
Pubis (MDA-Ma3–23), height of prepubic constriction (eroded ventral margin)	61

#### Etymology

The specific name refers to Haute-Garonne, the department in southern France where this lambeosaurine has been found.

#### Diagnosis

Lambeosaurine hadrosaurid differing from all other hadrosaurid taxa, except *Aralosaurus tuberiferus*, in having rostrodorsal region expanded in the form of prominent subrectangular flange that rises vertically above rostroventral process. *Canardia garonnensis* differs from *A. tuberiferus* in displaying subhorizontal (i.e., parallel to the caudal segment of tooth row) ectopterygoid shelf and prefrontal with dorsomedial flange and narrow rostroventral process.

#### Holotype

MDE (Musée des Dinosaures d’Espéraza, France)-Ma3–16, a nearly complete right maxilla (Fig. 3).

#### Referred material

MDE-Ma3–12 (partial left scapula), MDE-Ma3–15 (partial left maxilla), MDE-Ma3–17 (left quadrate), MDE-Ma3–18 (right prefrontal), MDE-Ma3–19 (partial left surangular), MDE-Ma3–20 (left humerus), MDE-Ma3–21 (partial left scapula), MDE-Ma3–23 (partial right pubis), MDE-Ma3–24 (partial left sternal plate), MDE-Ma3–25 (dentary tooth crown), MDE-Ma3–26 (partial right dentary dental battery), MDE-Ma3–28 (articular), MDE-Ma3–29 (partial right prefrontal), MDE-Ma3–30 (partial right pterygoid), and REP-LCR (private collection of Dominique Téodori, France) k6-001 (partial right quadrate and nearly complete right maxilla). Laurent [17] regarded the MDE-Ma3 material as representing a single individual. However, the presence of two left scapulae indicates that the recovered material represents at least two specimens. Because it is uncertain which bones belong to which individual and the sample represents an indeterminate number of specimens, it was deemed more conservative to consider solely one bone (a right maxilla) as the holotype.

#### Occurrence

The holotype and referred material of *Canardia garonnensis* came from the Tricouté 3 locality of the Aurignac anticline (southwest of Marignac-Laspeyres, in the Petites-Pyrénées, approximately 65 km southwest of Toulouse, Haute-Garonne Department, southern France), in the basal section of the upper Maastrichtian Marnes d’Auzas Formation [17]. According to Laurent [17], the fossil bones were found piled up on top of each other in a small lenticular body of marly soundstone within a thick sandstone bed.

### Description and Comparisons

#### Maxilla

The right maxilla is missing the dorsal process above the articular facet for the jugal, the caudal fourth of the dental battery, the palatine ridge, and the caudomedial region of the ectopterygoid shelf (Fig. 3). The left maxilla lacks the rostral third and the dorsal process above the articular facet for the jugal (Fig. 4). The most remarkable feature of the maxilla of *Canardia garonnensis* is that the rostrodorsal corner of the lateral surface of the maxilla, caudodorsal to the rostroventral process, forms a prominent flange rostral to the facet for the rostral process of the jugal. As noted by Bilotte et al. [18], aside from MDE-Ma3–16 this condition is only observed so far in *Aralosaurus tuberiferus* (upper Santonian-lower Campanian Bostobynskaya Formation of central Kazakhstan; [42], [43] and in a specimen (REP-LCR-k6-001; Fig. 10) from the upper Maastrichtian Marly Limestones of Gensac cropping out at the Larcan locality, not far from the Tricouté 3 site (Haute-Garonne Department of southern France; Fig. 1). That maxillary flange adds to the rostrocaudal width of the base of the dorsal process, so that the preserved portion of the process is subrectangular and extends along nearly half of the length of the maxilla. The rostral half of the dorsal process that contains the rostrodorsal maxillary flange, and that underlies the lacrimal, is rostrocaudally wide as in *Aralosaurus tuberiferus*. In all other lambeosaurines in which this region of the maxilla is known, such as *Hypacrosaurus stebingeri* (e.g., MOR [Museum of the Rockies, Bozeman, USA] 549-6-19-8-9), *Amurosaurus riabinini* (e.g., AEHM [Amur Natural History Museum, Blagoveschensk, Russia] 1/12), and *Corythosaurus casuarius* (e.g., AMNH [American Museum of Natural History, New York, USA] 5338), the laterally-exposed rostrodorsal surface is narrow, tall, and triangular to finger-shaped in lateral profile. At the rostral end of the maxilla, the dorsal and ventral margins of the edentulous rostroventral process converge forming an angle of 28° and a sharp apex. The maxillary rostroventral process is ventrally deflected 22° relative to the long axis of the maxilla. Pendant rostroventral processes are also present, to a greater or lesser degree of deflection, in *Tsintaosaurus spinorhinus* [44], *Angulomastacator daviesi* [45], *Magnapaulia laticaudus* [46], and *Olorotitan ararhensis* [6].

**Figure 11:**
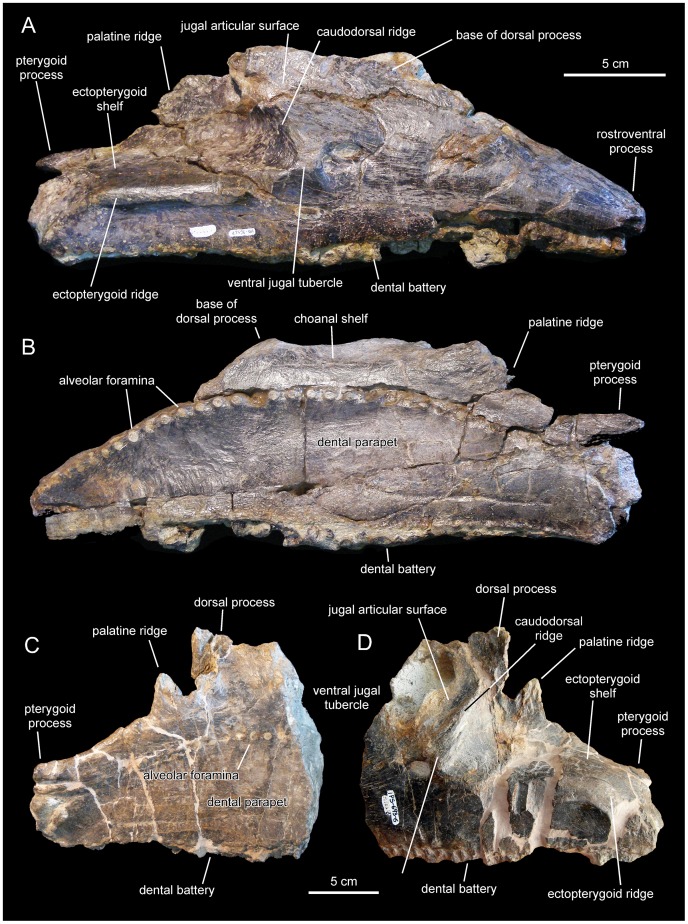
*Pararhabdodon isonensis*, maxillae. A. Right maxilla (IPS 36327) in lateral view. B. Medial view of same. C. Left maxilla (IPS 693-6) in medial view. D. Lateral view of same.

**Figure 12:**
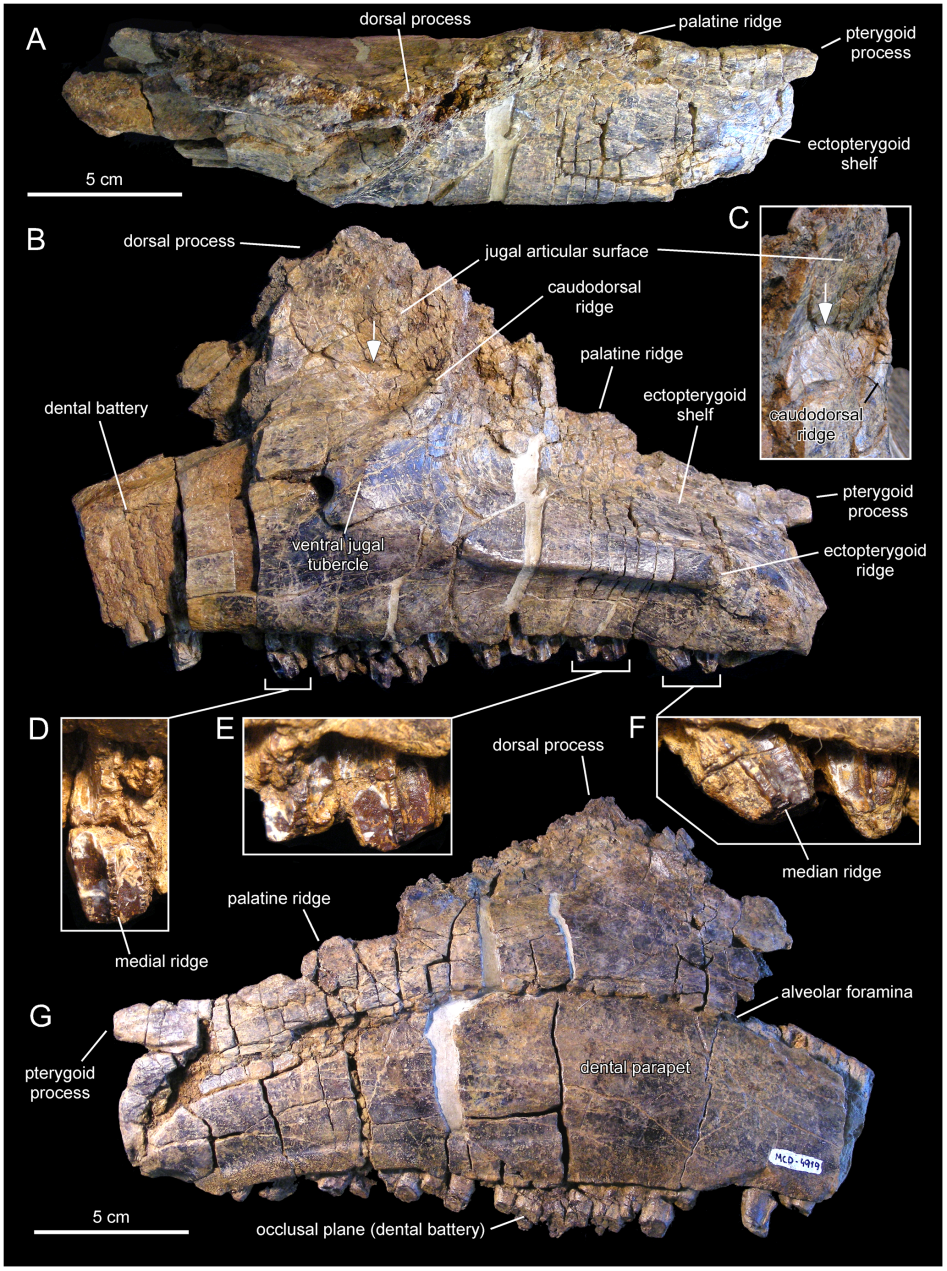
*Pararhabdodon isonensis*, MCD 4919, left maxilla. Maxilla in dorsal (A), lateral (B), and medial (G) views. A rostrolateral view of the lateral surface of the dorsal maxillary process, displaying breakage line and medial distortion (white arrow, also in A), is shown in C. Details of the lingual side of tooth crowns are presented in D–F.

**Figure 13:**
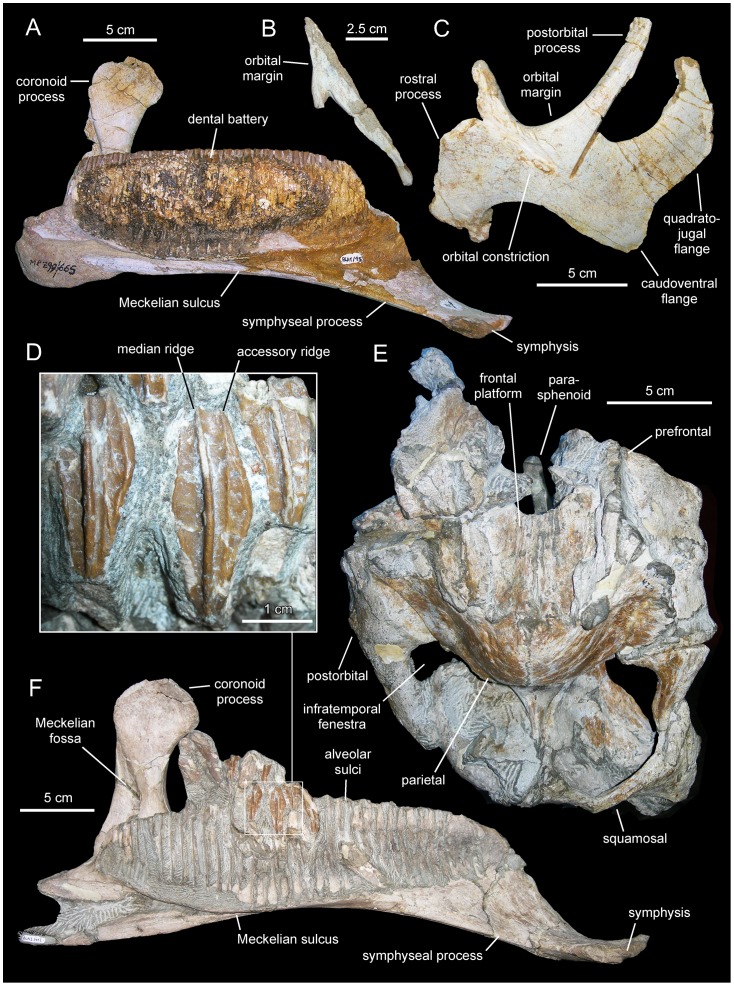
*Arenysaurus ardevoli* and *Blasisaurus canudoi*, selected cranial elements. A. Left dentary of *B. canudoi* (MPZ 99/665) in medial view. B. Right lacrimal of *B. canudoi* (MPZ 2009/348) in lateral view. C. Left jugal of *B. canudoi* (MPZ 99/667) in lateral view. D. Dentary tooth crowns of *A. ardevoli* (MPZ 2008/258) in lingual view. E. Caudal region of the skull roof of *A. ardevoli* (MPZ 2008/1) in dorsal view. F. Left dentary of *A. ardevoli* (MPZ 2008/258) in medial view.

As preserved in the more complete right maxilla, the mid-length of the base of the dorsal process is positioned slightly rostral to the mid-length of the maxilla. However, because the distal end of the maxilla is missing, when complete the base of the rostral process would lie more rostrally relative to the mid-length of the maxilla than it currently appears, similar to the condition in the Larcan specimen REP-LCR-k6-001 (Fig. 10). The articular surface for the rostral process of the jugal (the dorsal facet for the lacrimal is missing) is triangular, covering two thirds of the width of the dorsal process. This articular surface wedges rostrally, reflecting the presence of a similarly wedge-shaped rostral process in the jugal. The ventral tubercle of the jugal articular facet is prominent. There are four small foramina piercing the lateral surface of the maxilla below the ventral jugal tubercle. A sharp ridge borders ventrally the articular facet for the jugal. This ridge is caudally continuous with the ectopterygoid ridge, which extends caudal to the ventral jugal tubercle and is oriented parallel to the caudal segment of the tooth row. The maxilla of *Canardia* differs from that of *Pararhabdodon* (Fig. 11–13) in that the ectopterygoid ridge ends rostrally against the base of the articular surface of the jugal, as in all hadrosaurids except *Tsintaosaurus* and the aforementioned Spaniard genus where the ridge ends below that articular surface and forms an embayment curving dorsocaudally and transforming into a caudodorsally ascending margin [47] (Fig. 12A and D).

The ectopterygoid ridge becomes gradually thicker near its caudal end. The ectopterygoid shelf extends medial to the ectopterygoid ridge and gently slopes lateroventrally. The partially preserved rostral region of the palatine flange rises dorsally from the medial border of the ectopyerigoid ridge.

#### Prefrontal

The prefrontal is a crescentic element that forms the rostrodorsal corner of the orbit (Fig. 5A, D–F). The orbital margin is rugose in texture and becomes thicker at its caudal end. Rostrally, the orbital margin becomes gradually thinner as it bounds the rostrodorsal corner of the orbit, forming a sharp edge at the rostroventral end of the prefrontal at the contact with the lacrimal. The dorsal surface above the orbit is smooth and gently concave. The ventral orbital surface is smooth and arches rostrocaudally.

The entire medial border of the prefrontal is dorsally and slightly medially expanded due to the presence of a longitudinal ridge. Although this ridge is very low along the rostroventral segment of the prefrontal, it gradually becomes higher toward the caudomedial corner of the bone until it rises into a thin triangular and caudodorsally-projected flange (Fig. 5A, D). The caudodorsal end of the flange is incomplete; as preserved, the caudodorsal apex of the flange ends rostral to the caudal margin and caudolateral orbital corner of the prefrontal. A dorsomedial flange is present in all hadrosaurids [2], [48], with the exception of *Aralosaurus tuberiferus*. Both Rozhdestvensky ([41]: fig. 8) and Godefroit et al. ([42]: fig. 2A, pl. 1A) figured the left prefrontal articulated in the skull roof of *A. tuberiferus*, showing no sign of a dorsomedial flange. Indeed, neither Rozhdestvensky [41] nor Godefroit et al. [42] mentioned the presence of a prefrontal dorsomedial flange in the description of *A. tuberiferus*. In *Canardia garonnensis* the dorsomedial flange is lower than that of other lambeosaurines like, for example, *Lambeosaurus lambei* (e.g., CMN [Canadian Museum of Nature, Ottawa, Canada] 2869), *Hypacrosaurus stebingeri* (e.g., MOR 553S-7-27-2-93), or *Corythosaurus casuarius* (e.g., AMNH 5338). However, during lambeosaurine ontogeny the flange becomes more vertical and caudally developed [49], changes that are associated with the progressively greater development of the supracranial crest that the flange helps support. Thus, the low prefrontal crest of *C. garonnensis* may be an indicator of the immature nature of the specimen rather than a diagnostic or phylogenetically informative condition. The lateral surface of the dorsomedial flange of *C. garonnensis* is carved by a longitudinal groove and a series of four elliptical foramina. The caudal margin of the flange is excavated and displays a gently concave profile in lateral view.

The medial surface of the prefrontal shows a shallow wide longitudinal groove rostral to the dorsomedial flange. A sharp ridge separates this groove from the ventral orbital surface of the rostroventral process of the prefrontal. The grooved medial surface and the ventral orbital side of the prefrontal are orthogonal relative to each other. As in the majority lambeosaurines, the rostroventral process of the prefrontal is rostrocaudally narrow; this stands in contrast to the broad laterally well-exposed rostroventral process of the prefrontal of *Aralosaurus tuberiferus* ([41]: fig. 8, [42]: fig. 2A) and saurolophine hadrosaurids [2].

#### Postorbital

All that remains of the postorbital is a fragment of its central body containing the proximal regions of the rostral and jugal rami (Fig. 5B, C). The rostral ramus is dorsoventrally compressed, whereas the jugal one is compressed rostrocaudally. The ventral surfaces of these two rami enclose the caudodorsal concave orbital surface. The lateral margin of the caudodorsal corner of the orbit is relatively thin, in contrast to the substantially thicker medial border. The latter shows a crenulated texture for articulation with the frontal. At the center of the medial surface of the central body of the postorbital there is a subcircular excavation. This excavation constitutes the laterosphenoid enarthrosis, which is delimited by well-defined margins from the remaining bone surface. The medial surface of the postorbital that lies caudal and adjacent to the laterosphenoid enarthrosis is laterally offset relative to the rest of the medial margin of the bone.

#### Quadrate

As in other lambeosaurines ([50]: fig. D76), the quadrate of *Canardia garonnensis* (Fig. 5G–J) is caudodorsally curved, a condition that is more evidently observable along the caudal margin of the bone. Specifically, the long axis of the quadrate dorsal to the quadratojugal notch forms an angle of 147° relative to the long axis of the rest of the element. The quadrate head is mediolaterally compressed and triangular in cross section. The quadratojugal notch is widely arcuate and its dorsal and ventral margins are approximately equal in length. The center of the notch lies ventral to the mid-length of the quadrate, as in many basal hadrosauroids [51] and saurolophine hadrosaurids [2]. Only the central region of the pterygoid flange is incompletely preserved. The fragmentary central region of that flange projects rostromedially from the medial margin of the quadrate. Most of the medial surface of the pterygoid flange is occupied by a deep fossa; in contrast, the lateral side is dorsoventrally convex (Fig. 5I, J). The ventral end of the quadrate is transversely expanded into two condyles, which together form a laterally skewed triangular profile in distal view. The much larger lateral condyle articulates with the surangular and displays an equilateral triangular cross section. This condyle is greatly offset ventrally relative to the medial condyle. The latter constitutes a relatively small spur that projects medially form the distal end of the quadrate.

#### Pterygoid

This tetraradiate bone consists of a medially buttressed central region from which two large flanges extend rostrodorsally and caudodorsally, and two shorter but robust processes project rostroventrally and caudoventrally (Fig. 6). The only recovered pterygoid is missing the dorsal margin of the palatine flange and most of the dorsal and caudal regions of the alar quadrate flange. The alar quadrate flange is a thin bony sheet that extends caudodorsally to contact the concave medial surface of the pterygoid flange of the quadrate. Ventral to the alar flange, a shorter but relatively thick process projects caudoventrally to abut the strongly depressed caudoventral extent of the medial flange of the quadrate, adjacent to the caudal margin of the latter. On the opposite side of the pterygoid, the preserved rostroventral half of the palatine flange projects rostrodorsally to contact the dorsal margin of the palatine. Ventral to the palatine flange is the ectopterygoid process. This process, the shortest of the radial rami of the pterygoid, is dorsoventrally compressed and tapers distally. Its rostromedial surface contacts the caudal surface of the maxilla, whereas rostrolaterally it articulates with the caudomedial region of the ectopterygoid. A relatively large D-shaped excavation for reception of the ectopterygoid exists on the lateral surface of the pterygoid, between the palatine flange and the ectopterygoid process and adjacent to the rostral edge of the pterygoid (Fig. 6A).

On the medial side of the pterygoid, three prominent large ridges converge at the center of the element. The convergence of these ridges forms a thick dorsoventrally compressed buttress; in hadrosaurids this buttress contacts the basipterygoid process of the basisphenoid [52]. One of the three ridges extends rostrodorsally onto the palatine flange. A second ridge extends caudoventrally from the central buttress onto the medial surface of the caudoventral process. The third ridge extends ventrally and slightly rostrally from the central buttress, merging with the medial edge of the ectopterygoid process. Thus, in medial view the rostroventral and caudoventral ridges define an A-shaped profile, the apex of which constitutes the central buttress (Fig. 6B). The space enclosed by the rostroventral and caudoventral ridges is bounded laterally by a bony lamina. This lamina also constitutes the ventral region of the lateral surface of the central plate of the pterygoid. Only the base of the dorsomedian peg-like process, that lies between the proximal regions of the alar quadrate and the vaulted palatine flanges, is preserved.

#### Surangular

The surangular is a rostrocaudally gently arcuate post-dentary element in the mandible of *Canardia garonnensis* (Fig. 7A–C). The rostral ascending flange is only preserved proximally. It curves rostrodorsally and its mediodorsal surface shows a large concavity that ends caudally near the central region of the surangular. The lateral margin of the central region of the surangular is expanded laterally to form a thick and D-shaped lip. Its dorsal surface is slightly convex rostrocaudally and contains a shallow longitudinal ridge. The caudal slope of the lateral lip is continuous with the glenoid facet that received the quadrate. On the medial side of the surangular, a longitudinal prominent ridge constitutes the dorsal border of the long shallow articular facet for the angular, and separates this facet from the articular surface for the splenial above (Fig. 7C). The splenial articular surface is laterally recessed relative to that for the angular, and extends dorsally to form a sharp thin flange. The caudal end of the surangular is composed of the mediolaterally compressed caudal process. The distal tip of the process is missing. The articular contacts the relatively smooth mediodorsal surface of the retroarticular process. In dorsal view, caudal process curves laterally, a widespread condition among lambeosaurine hadrosaurids [2].

#### Articular

The articular is a relatively small saddle-shaped post-dentary element (Fig. 7D, E). It is mediolaterally compressed, more so ventrally than dorsally, and it contributes to form with the surangular the retroarticular process of the mandible. The concave lateral surface of the articular contacts the medial surface of the caudal process of the surangular, whereas the convex medial surface of the articular meets the lateral surface of the splenial.

#### Dentition

**Figure 14:**
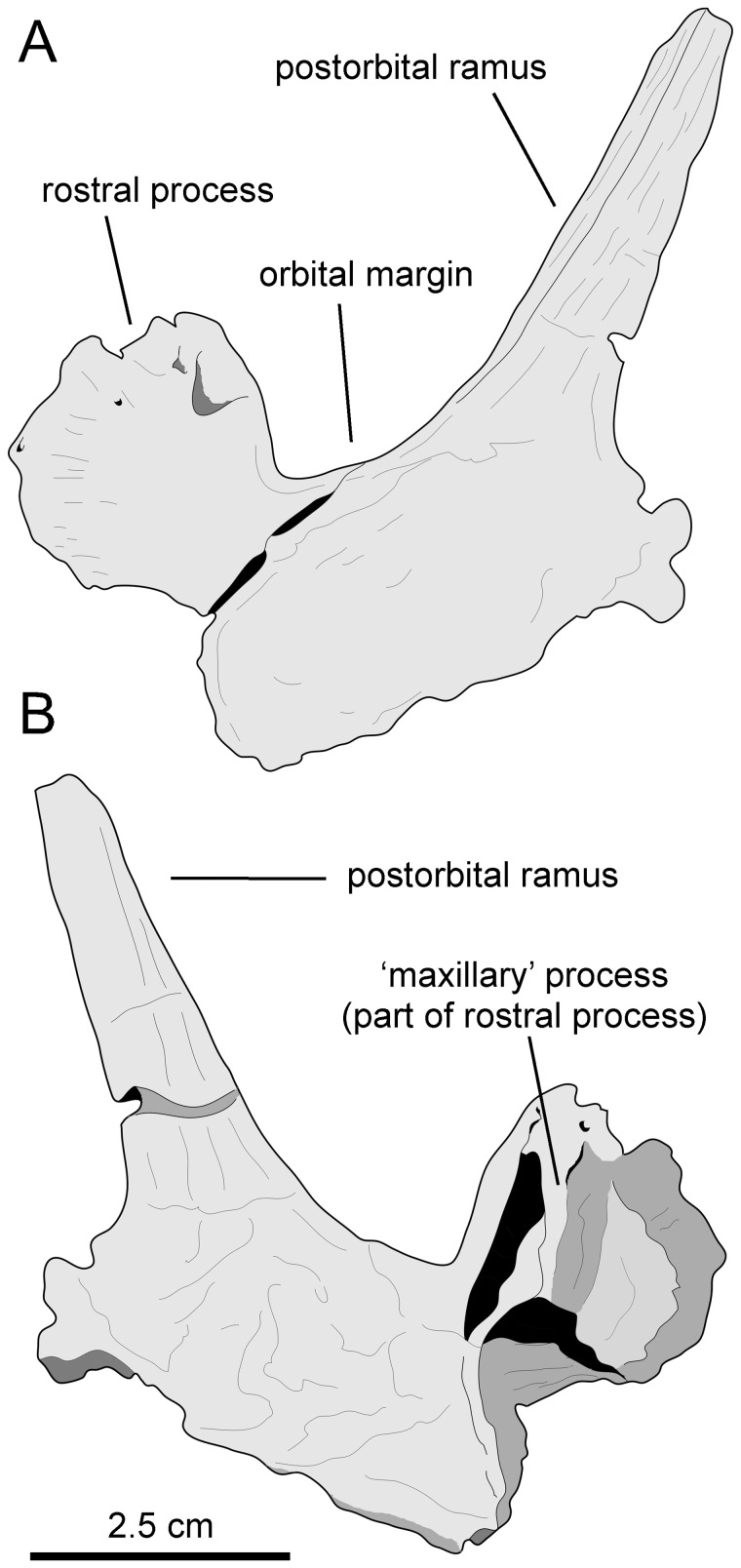
*Arenysaurus ardevoli*, left jugal. A. Lateral view of partial left jugal (MPZ 2011/01). B. Medial view of same. Drawings based on the photographs in Cruzado-Caballero ([66]: fig. 4.83).

The number of alveoli in the dentary is unknown, since both this element and its dentition are only represented by a fragmentary dental battery (Fig. 8A–C, E). The fragment contains 11 tooth positions. The occlusal surface shows a maximum of two functional teeth arranged transversely. However, a count of up to three teeth exposed occlusally, as occurs in Hadrosauridae [2], cannot be ruled out since the fossil fragment probably corresponds to a mesial or distal region of the dental battery. Tooth crowns are lanceolate and, on average, have a height/width ratio of 3.7. Unlike in *Blasisaurus canudoi* (Fig. 14A), the mesial margins of the tooth crowns do not overlap the distal margins of preceding teeth. In *Canardia garonnensis* the enameled surface of each crown shows a prominent ridge that is slightly offset distally form the midline. In addition, there is a finer subsidiary ridge positioned near the mesial margin of the tooth. One or two subsidiary ridges are commonly present among lambeosaurine teeth [50]. However, in lambeosaurines (e.g., *Lambeosaurus lambei*, CMN 2869) these subsidiary ridges are typically not as continuous as in *C. garonnensis*, in which the ridge extends apicobasally along the entire length of the crown. In this regard, the pattern and development of ridges in *C. garonnensis* is very similar to that in *Arenysaurus ardevoli* (Fig. 14D), IPS (Institut de Paleontologia Miquel Crusafont, currently Institut Català de Paleontologia ‘Miquel Crusafont’, Sabadell, Spain) 36338 (an isolated left dentary of an indeterminate hadrosauroid unearthed from uppermost Maastrichtian strata of the Tremp Formation at the Fontllonga locality, Lleida province, northeastern Spain [53], and MGUV 2200 and MPV (Museo Paleontológico de Valencia, Valencia, Spain) 181 (dentaries from La Solana locality of an unnamed upper Maastrichtian unit correlated with the Villalba de la Sierra Formation, Valencia Province, eastern Spain [54], [55]). Denticles, in the form of small papillae, are so reduced in size that they appear to be absent to the naked eye. Among lambeosaurines, the apparent absence or extreme reduction of marginal denticulation occurs in *Arenysaurus ardevoli*, *Blasisaurus canudoi*, *Amurosaurus riabinini*, *Sahaliyania elunchunorum Hypacrosaurus altispinus*, and *Charonosaurus jiayinensis* [2], [11], [12].

Neither of the two available maxillae preserves complete dental batteries. The right maxilla shows 26 tooth positions (MDE-Ma3–16; Fig. 3) and the left one 20 (MDE-Ma3–15; Fig. 4). Maxillary tooth crowns show a single straight ridge centered at the midline of the enameled surface; no subsidiary ridges are present (Fig. 4C, D). As in the dentary, denticles are extremely reduced papillae.

#### Sternal plate

The sternal plate of *Canardia garonnensis* shows an incompletely preserved medial margin of the craniomedial expansion and lacks the distal segment of the caudolateral process (Fig. 9B). The sternal plate is strongly compressed dorsoventrally. It becomes thicker medially, particularly mediodorsally. The craniomedial expansion is fan-shaped. The craniomedial process branches out from the caudomedial corner of the plate. Only the relatively broad proximal region of this process is preserved, which projects caudomedially at about 90° from the long axis of the caudolateral process. The lateral margin of the lateral process is continuous with the lateral margin of the craniomedial expansion.

#### Scapula

As it occurs in hadrosaurids [2], the scapula of *Canardia garonnensis* displays gently curved dorsal and ventral margins (Fig. 9A, C). The cross-section of the bone is aerofoil-shaped through most of the length of the scapular blade. In the most complete scapula, MDE-Ma3–21, the dorsal margin of the proximal constriction and the pseudoacromion process are distorted, having been pushed ventrally over the deltoid fossa from their original position (Fig. 9A). Thus, characters like the relative breadth of the proximal scapular constriction, the orientation of the pseudoacromion process, and the prominence of the deltoid ridge cannot be ascertained. The proximal region of MDE-Ma3–21 (the only recovered scapula so far that includes the proximal articular region) contains incompletely preserved and heavily distorted coracoid and glenoid facets. The glenoid is mediolaterally narrower than the coracoid facet. Ventrally, the glenoid and the proximoventral margin of the scapula converge forming an apex. Except for their distal ends, most of the scapular blade is preserved in the two recovered scapulae of *C. garonnensis*. MDE-Ma3–12 shows the most complete scapular blade; as preserved in this specimen, the maximum distal expansion of the blade is 1.9 times the width of the proximal edge (which approximately corresponds to the proximal constriction of this scapula). This indicates a relatively deep distal scapular blade, a condition that is prevalent among lambeosaurines [56].

#### Humerus

This element is known from a single specimen (Fig. 9E–G). At the caudodorsal corner of the bone, the humeral head is heavily eroded. The deltopectoral crest projects cranially from the proximal region of the bone, as in the saurolophine *Wulagasaurus dongi* [57], and the lambeosaurines *Parasaurolophus cyrtocristatus* (e.g., FMNH [The Field Museum, Chicago, USA] P27393) and *Arenysaurus ardevoli* (according to Pereda-Suberiola et al. [11]; but see below). The crest accounts for slightly more than half of the total length of the humerus, as in most hadrosaurids [58]. Specifically, the ratio between the maximum width of the crest and the minimum craniocaudal diameter of the humeral shaft is 1.65. Such a ratio falls well below the ratios found in lambeosaurines, which are typically in excess of 1.9 ([50]: fig. H16). However, given the possibility that MDE-Ma3–20 represents a juvenile specimen, this ratio may reflect an immature condition; indeed, the expansion of the deltopectoral crest has been shown to increase during ontogeny in hadrosauroids [51]. The lateral margin of the deltopectoral crest becomes more than twice as thick towards its angular ventral margin. The humeral shaft is subcircular in cross section. A shallow poorly developed median tuberosity is present on the caudal surface of the humerus, nearer the mid-length of the bone than to its proximal end. The distal end of the humerus is mediolaterally expanded to form the lateral ulnar and medial radial condyles. As is common in hadrosaurids [1], the ulnar condyle, the distal surface of which is heavily eroded, is substantially wider and more craniocaudally expanded than the radial condyle.

#### Pubis

The single available pubis is heavily abraded, lacking most of the distal blade of the prepubic process, part of the ischiadic process, and the entire postpubic process (Fig. 9D). The prepubic process preserves the proximal constriction and a fragmentary distal blade, which is, like the rest of the pubis, mediolaterally compressed. Although its ventral margin is incompletely preserved, the proximal constriction is still relatively wide dorsoventrally. Specifically, the minimum breadth of the proximal constriction is 75% of the maximum breadth of the acetabular margin. The point of maximum concavity of the dorsal margin of the proximal constriction lies caudally relative to that of the ventral margin. Distally, the dorsal and ventral margins of the prepubic process are too incompletely preserved to gain any insight into the geometry of the prepubic blade. The tetrahedral iliac process projects caudodorsally from the dorsal extent of the acetabular region of the pubis. The gently arcuate caudal margin of this process forms nearly half of the acetabular margin. The acetabular surface of the process faces caudolaterally. Ventrally, the iliac process is continuous with the ventral half of the acetabular margin and the proximal region of the ischiadic process. The ischiadic process projects caudoventrally at approximately 140° from the long axis of the prepubic process.

#### Comments

Laurent [17] provisionally referred the MDE-Ma3 material to *Pararhabdodon* sp. because at the time this was the only lambeosaurine genus known from the Upper Cretaceous of Europe. Yet, none of those bones show characters diagnostic of *Pararhabdodon*. The articular surface for the jugal in MDE-Ma3–15 and 16 is as in all other hadrosaurids, i.e., the ventral margin and ventral jugal tubercle are continuous with the ectopterygoid ridge. In contrast, in *Pararhabdodon,* as well as in the closely related *Tsintaosaurus*, the ectopterygoid ridge is discontinuous with (and ends below) the articular surface for the jugal, forming an embayment that curves dorsocaudally [47] (Fig. 11D and 13B).

Recently, Bilotte et al. (2010) described the right maxilla and quadrate of a relatively small lambeosaurine specimen (REP-LCR-k6-001; Fig. 10) from the Larcan locality in the uppermost Maastrichtian of the Petites Pyrénées, Haute-Garonne, southern France. This specimen can be confidently distinguished from *Pararhabdodon isonensis* because it lacks the characteristic tsintaosaurin elevated jugal articulation surface described above. The Larcan lambeosaurine is referred to *Canardia garonnensis* because it possesses the maxillary characters given in the differential diagnosis for this species, i.e., the combination of a tall subrectangular rostromedial flange of the maxilla and a subhorizontal ectopterygoid shelf.

### Tsintaosaurini, New Tribe

urn:lsid:zoobank.org:act:142EFF1A-A2BC-4DD7-96D9-E90DF276D738.

Fig. 2.

#### Definition

The most exclusive clade of lambeosaurine hadrosaurids containing *Tsintaosaurus spinorhinus* Young, 1958 [44] and *Pararhabdodon isonensis* Casanovas-Cladellas, Santafé-Llopis, and Isidro-Llorens, 1993 [10].

#### Diagnosis

Lambeosaurine hadrosaurids possessing maxilla with elevation of articular facet for jugal, such that ectopterygoid ridge is discontinuous with, and ends below, articular surface for jugal; and maxilla showing acute embayment between jugal facet and ectopterygoid shelf, caused by continuation of ectopterygoid ridge into ascending border that curves dorsocaudally.

#### Type genus

*Tsintaosaurus* Young, 1958 [44].

*Pararhabdodon isonensis* Casanovas-Cladellas, Santafé-Llopis, and Isidro-Llorens, 1993 [10].

#### Holotype

IPS SRA 1, a nearly complete mid-caudal cervical vertebra.

#### Referred material

IPS 693-6 (caudal half of left maxilla, formerly IPS SRA 23), IPS 693-12 (fragment of proximal rib), IPS 693-13 (caudal middle dorsal vertebra), IPS 36327 (right maxilla, formerly IPS SRA 22), IPS SRA 12 (caudal middle dorsal vertebra), IPS SRA 13 (centrum of dorsal vertebra), IPS SRA 15 (left humerus), IPS SRA 16 (proximal fragment of left scapula), IPS SRA 17 (caudal vertebra), IPS SRA 18 (middle cervical vertebra), IPS SRA 20 (caudal middle dorsal vertebra), IPS SRA 24 (partial sacrum), IPS SRA 25 (middle cervical vertebra), IPS SRA 26 (distal end of right ischium), MCD (Museu de la Conca Dellà, Isona, Spain) 4730 (cranial cervical vertebra), MCD 4731 (cranial dorsal vertebra), and MCD 4919 (partial left maxilla).

#### Occurrence

All the available material of *Pararhabdodon isonensis* came from upper Maastrichtian strata of the Tremp Formation cropping out at two localities near the town of Isona, Lleida province, in the northeastern Tremp Syncline of northeastern Spain (Fig. 1). The holotype and referred specimens other than MCD 4919 were excavated from the Sant Romà d’Abella locality, at the base of the Tossal de la Doba hill; MCD 4919, here reported for the first time, comes from the Serrat del Rostiar 1 locality, near the village of Basturns. The Sant Romà d’Abella locality occurs in the upper section of the ‘lower red unit’ of the Tremp Formation, and therefore, is younger than Serrat del Rostiar 1, which is in the middle-lower section of the ‘lower red unit’ of the Tremp Formation, 30 m above a horizon that is in the C31r (Fig. 2). Referral of MCD 4919 to *Pararhabdodon isonensis* extends the chronostratigraphical range of this species down within the upper Maastrichtian.

#### Description of the maxilla MCD 4919

This element is missing its rostral end (including the premaxillary dorsal flange and articular surface, and the rostroventral dentigerous margin), as well as the apex of the dorsal process; otherwise, the maxilla is relatively complete (Fig. 12 and Table 3). The fragmentary rostral end of the specimen exposes laterally four alveolar positions of the dental battery that are filled with partially eroded teeth. Up to four teeth are present arranged dorsoventrally within each alveolar position.

**Table 3:** Selected maxillary measurements (in mm) of *Pararhabdodon isonensis*.

Element	Measurement
Maxilla (IPS 36327), total length	322
Maxilla (IPS 36327), length of ectopterygoid shelf	120
Maxilla (IPS 36327), height from alveolar margin to highest point of dorsal process (incomplete)	103
Maxilla (IPS 693-6), total length (missing rostral half of bone)	165
Maxilla (IPS 693-6), length of ectopterygoid shelf	101
Maxilla (IPS 693-6), height from alveolar margin to highest point of dorsal process (incomplete)	126
Maxilla (MCD 4919), total length (missing most of rostral half)	243
Maxilla (MCD 4919), length of ectopterygoid shelf	148
Maxilla (MCD 4919), height from alveolar margin to highest point of dorsal process (incomplete)	134

The morphology of the articular facet for the jugal and lacrimal bones is distorted, having collapsed medially (Fig. 12C). Signs indicative of this crushing include a fracture extending rostrocaudally (arrows in Fig. 12C and D) and multiple cracking on the bone surface. This facet occupies the lateral surface of the preserved dorsal process of the maxilla. MCD 4919 displays two foramina ventral to the articular facet for the jugal. The more dorsal of the two is relatively large and opens rostrolaterally. A much smaller foramen is located caudoventral to the large foramen. IPS 36327 (paratype of *Pararhabdodon isonensis*) and maxillae of *Tsintaosaurus spinorhinus* ([44]: fig. 7) also show a pair of foramina of similar size ventral to the jugal facet. As preserved, the dorsal process shows a D-shaped lateral profile and, although missing its apex, it is relatively tall as in other lambeosaurines [1]. The height of the maxilla from the alveolar margin to the dorsal margin of the dorsal process is over twice the maximum height of the rostral region of the bone.

The ectopterygoid ridge is prominent thick, gradually becoming thicker caudally. The ventral edge of this ridge is sharply defined, in contrast to the smooth poorly delimited dorsal border. The ectopterygoid shelf is nearly horizontal, subparallel to the tooth row, and comprises approximately half of the length of the preserved maxilla. In the complete maxilla, the ectopterygoid shelf would probably account for more than 35% of the total length of the maxilla. The palatine ridge forms a large flange that projects mediodorsally form the dorsal border of the maxilla, medial to the ectopterygoid shelf. The dorsal margin of the palatine process is irregular and incomplete. Caudally, this margin is continuous with the pterygoid process. This process is finger-like and transversely narrow, projecting caudally from the caudomedial border at the end of the maxilla. The caudal tip of the process is not preserved.

The medial surface of the MCD 4919 maxilla is nearly flat and shows a gently arcuate row of alveolar foramina. As in other hadrosaurids [2], this row of foramina is positioned dorsal to the mid-depth of the maxilla and separates the choanal shelf above from the dental parapet below. The choanal shelf slopes lateroventrally and the surface immediately below the shelf becomes gently convex adjacent and dorsal to the row of alveolar foramina. The tooth battery is entirely covered by a dental parapet. The dental parapet is gently convex dorsoventrally throughout most of its surface dorsoventrally; ventrally, however, it becomes nearly flat.

There are 27 tooth positions preserved, most of them holding tooth crowns still in place. Given the substantial portion missing at the rostral region of the maxilla, the total number of teeth probably reached a minimum of 32, a hadrosaurid synapomorphy [2]. There are two functional teeth throughout most of the dental battery length, gradually changing to one near the rostral and caudal ends of the maxilla. Each tooth shows a single straight carina located symmetrically at the center of the crown, with no additional ridges (Fig. 12D–F). No denticles can be unambiguously observed.

MCD 4919 possesses tsintaosaurin synapomorphies: a jugal facet for the maxilla that is elevated such that it is entirely above the level of the lateral margin of ectopterygoid shelf and the latter being discontinuous with, and ending below the articular surface for the jugal, forming an embayment that curves into a caudodorsal ridge (Fig. 12B and C; see also Prieto-Márquez and Wagner, 2009). It must be noted that additional preparation of one of the two maxillae recovered for *Pararhabdodon isonensis*, IPS 693-6 (Fig. 11C and D), reveals the most of the dorsoventral extent of the articular facet for the jugal. Notably, the ventral margin of the facet and the ventral jugal tubercle lie several millimeters above the level of the ectopterygoid shelf, which is a more ventral boundary (Fig. 11D) than previously shown by Prieto-Márquez and Wagner ([47]: fig. 2E). A comparable position of the ventral margin of the jugal facet is observed in MCD 4919 (Fig. 12B). A caudodorsal ridge, continuous with the ectopyerygoid ridge, bounds caudally the articular surface for the jugal in IPS 693-6 and MCD 4919.

Prieto-Márquez and Wagner [47] distinguished *Pararhabdodon isonensis* from *Tsintaosaurus spinorhinus* on the basis of the relative rostrocaudal breadth of the rostrodorsal region of the maxilla, being greater in the former than in the latter. Although the rostral extent of the dorsal process and rostrodorsal region of MCD 4919 is missing, enough is preserved of the dorsal process to show that it is rostrocaudally broader than the narrow subtriangular process of *T. spinorhinus*. Thus, we refer this maxilla to *Pararhabdodon isonensis*.

### Lambeosaurini Parks, 1923 [40]

#### Definition

*Lambeosaurus lambei* Parks, 1923 [40] and all lambeosaurine taxa more closely related to it than to *Parasaurolophus walkeri* Parks, 1922 [59], *Tsintaosaurus spinorhinus* Young, 1958 [44], or *Aralosaurus tuberiferus* Rozhdestvensky, 1968 [41].

#### Diagnosis

Lambeosaurine hadrosaurids possessing vertical groove on lateral process of premaxilla, located rostral to dorsal process of maxilla and extending ventrally from small opening between premaxillary medial and lateral processes; vertical groove bounded rostrally by triangular ventral projection of lateral process of the premaxilla; nasal articulation surface for frontal shaped into rostroventrally-sloping platform; nasal vestibule folded into S-loop in enclosed premaxillary passages rostral to dorsal process of maxilla; and lateral premaxillary process extending caudodorsal to prefrontal in adults.

#### Type genus

*Lambeosaurus* Parks, 1923 [40].

#### Comments

The vast majority of phylogenetic analyses of Lambeosaurinae published over the last decade have recovered two main lambeosaurine clades, the *Parasaurolophus*-clade and another clade including helmet-crested genera like *Corythosaurus*, *Hypacrosaurus*, and *Lambeosaurus* [2], [6], [11], [12], [46], [49], [57], [60]–[66]. The *Corythosaurus*-*Lambeosaurus* clade has been referred to as Corythosaurini in the literature [12], [64], [65], [67], [68], albeit this name has never received a formal definition and diagnosis. However, as correctly pointed out by Sullivan et al. [69], the corresponding name for that clade must be Lambeosaurini. This is because according to article 37.1 of the International Code of Zoological Nomenclature, “When a family-group taxon is subdivided, the subordinate taxon that contains the type genus of the superior taxon is denoted by the same name (except for suffix) with the same author and date [Art. 36.1]; this subordinate taxon is termed the ‘nominotypical taxon’” [70].

### *Arenysaurus ardevoli* Pereda-Suberbiola, Canudo, Cruzado-Caballero, Barco, López-Martínez, Oms, and Ruiz-Omeñaca, 2009 [11]

#### Holotype

MPZ (Museo Paleontológico de la Universidad de Zaragoza, Zaragoza, Spain) 2008/1, a partial articulated braincase and skull roof (Fig. 13E).

#### Referred material

MPZ 2008/256 (fragment of right maxilla), 2008/257 (fragment of left maxilla), 2008/258 (left dentary bearing 12 teeth), 2008/259 (right surangular), 2008/260–263 (four isolated teeth), 2008/333 (partial right scapula), 2008/334 (right coracoid) 2008/336 (right humerus), 2008/335 (fragment of right ilium), 2007/707 (right pubis), 2007/711 (right femur), 2008/337 (left femur), and numerous axial bones (Pereda-Suberbiola et al., 2009a).

#### Occurrence

Blasi 3 locality, a bed within the ‘grey unit’ at the base of the Tremp Formation, uppermost Maastrichtian (upper part of C30n) cropping out in the western side of the Tremp Syncline (near the village of Arén, Huesca province, northeastern Spain; Fig. 1) [11].

#### Emended diagnosis

Lambeosaurine hadrosaurid possessing autapomorphic, extremely prominent frontal dome that rises at least to level of dorsal margin of medial ramus of squamosal.

#### Comments

The holotype and referred materials of *Arenysaurus aredevoli* constitute the most complete cranial remains recovered so far from a hadrosaurid in Europe. Pereda-Suberbiola et al. (2009a) diagnosed *Arenysaurus ardevoli* based on the following autapomorphies: very prominent frontal dome, more so than in other adult lambeosaurines; nearly vertical prequadratic and jugal processes of the squamosal and postorbital, respectively; and cranially oriented deltopectoral crest of the humerus. These authors also added to the diagnosis of *A. ardevoli* the following unique combination of characters: caudal length/width ratio of the frontals estimated in 0.5; midline ridge of the parietal at the level of the postorbital-squamosal bar; parietal excluded from the occipital margin of the skull; and lateral side of the squamosal relatively low above the condyloid cavity. From all these characters, we have only retained the extremely high frontal dome in the revised diagnosis of *A. ardevoli*.

As seen in Fig. 3C of Pereda-Suberbiola et al. [11], the putative nearly vertical orientation of the jugal ramus of the postorbital and the prequadratic process of the squamosal stems from orienting the squamosal ramus of the postorbital subhorizontally. Actually, however, in articulated lambeosaurine skulls (e.g., *Lambeosaurus lambei*, ROM (Royal Ontario Museum, Toronto, Canada) 1218; *L. magnicristatus*, CMN 8705; *Corythosaurus casuarius*, AMNH 5240; *Hypacrosaurus stebingeri*, MOR 455) the caudal region of the skull roof is, to a greater or lesser degree, commonly tilted caudoventrally relative to the long axis of the maxillary tooth row, so that the prequadratic and jugal processes project rostroventrally. Nonetheless, in many (e.g., *C. casuarius*, TMP 84.121.1; *C. intermedius*, CMN 8703 or ROM 776) although not all (e.g., *C. casuarius*, AMNH 5240) lambeosaurine specimens, the prequadratic squamosal process and proximal region of the postorbital jugal ramus are nearly perpendicular to the the squamosal ramus of the postorbital above the infratemporal fenestra. These observations indicate that the orientation of the prequadratic process and jugal ramus of the postorbital of *A. ardevoli* are not autapomorphic.

Pereda-Suberbiola et al. [11] also considered the cranial orientation of the deltopectoral crest of the humerus as autapomorphic for *Arenysaurus ardevoli*. Yet, such orientation is not exclusive of MPZ 2008/336; it is also present in *Canardia garonnensis* (see description above), *Parasaurolophus cyrtocristatus* (FMNH P27393), and the saurolophine *Wulagasaurus dongi* [57]. Furthermore, Fig. 5 of Pereda-Suberbiola et al. [11] shows that an eroded low ridge is all that remains of the deltopectoral crest of *A. ardevoli*. This eroded remnant corresponds to the proximal-most base of the crest. Both its poor state of preservation and the fact that the vast majority of the crest is missing precludes an accurate assessment of the actual orientation of the deltopectoral crest.

Finally, the combination of characters given by Pereda-Suberbiola et al. [11] for diagnosing *Arenysaurus ardevoli* is not unique to this taxon. For example, Lambeosaurine neurocrania with frontals approximately twice as wide as they are long, exclusion of the parietal from the occipital margin of the skull, rostral half (the caudal segment is always rising in lambeosaurines [2]) of the parietal midline ridge of the parietal set at the level of the postorbital-squamosal bar, and lateral side of the squamosal relatively low above the condyloid cavity are present in other taxa; examples are *Hypacrosaurus stebingeri*, MOR 553S-7-27-2-93, and *H. altispinus* CMN 8675. Furthermore, a lateral side of the squamosal relatively low above the quadrate cotylus, defined as such, is applicable to most lambeosaurine skulls, unless “relatively low” is more objectively described. Nonetheless, observation of MPZ 2008/1 reveals that the position of the lateral surface of the squamosal above the cotyloid cavity is no different from that of any other lambeosaurine we have observed.

### *Blasisaurus canudoi* Cruzado-Caballero, Pereda-Suberbiola, and Ruiz-Omeñaca, 2010 [12]

#### Holotype

MPZ 99/667, a left jugal (Fig. 13C)

#### Referred material

MPZ 99/666 (fragmentary left maxilla), 2009/348 (right lacrimal; Fig. 13B), 99/665 (left dentary; Fig. 13A), and 99/664 (right surangular).

#### Occurrence

Blasi 1 locality, corresponding to the uppermost Maastrichtian (upper part of C30n; Fig. 2) topmost horizon of the Arén Formation, which outcrops in the western side of the Tremp Syncline (near the village of Arén, Huesca province, northeastern Spain; Fig. 1) [11], [12], [21].

#### Emended diagnosis

Lambeosaurine hadrosaurid characterized by the following autapomorphies: hook-shaped quadratojugal flange of jugal due to relatively elongated rostrally recurved dorsal process; and strongly asymmetrical, caudally skewed lateral contour of ventral flange of the jugal, so that rostroventral margin of flange is twice as long as its caudoventral border. Furthermore, *Blasisaurus canudoi* can be distinguished from other lambeosaurines by possessing the following unique combination of three jugal characters: orbital margin being wider than infratemporal margin, concave caudoventral margin of the ventral flange, and length/height ratio of jugal less than 1.2 (diagnosis modified from Cruzado-Caballero et al., 2010a).

#### Comments

Originally, Cruzado-Caballero et al. [12] provided the following autapomorphies for *Blasisaurus canudoi*: jugal with hook-like dorsal process of the quadratojugal flange and relatively narrow D-shaped infratemporal fenestra. Of these, the D-shaped morphology of the infratemporal fenestra has not been retained in the revised diagnosis because the jugal only accounts for the ventral border of the entire outline geometry of the fenestra. This outline depends also on the morphology of the nearby postorbital, squamosal, and quadrate bones. Furthermore, the ventral outline morphology of the infratemporal fenestra is variable intraspecifically (e.g., in *Corythosaurus casuarius* it is relatively narrow D-shaped in ROM 1933 but broad arcuate in ROM 871). Instead, it is the shape and rostrocaudal width of the rostral margin of the infratemporal fenestra (formed by the postorbital and squamosal) that may be phylogenetically or taxonomically informative ([50]: figs 27 and 28). Cruzado-Caballero et al. [12] also added a combination of characters to their diagnosis of *B. canudoi*: caudal margin of the rostral process projected ventrally into a straight line; concave caudoventral margin beneath infratemporal fenestra; and very short jugal with length/height ratio of less than 1.2. This combination of characters has been retained here, albeit with exclusion of the ventrally straight caudal margin of the rostral process of the jugal. The shape (and length) of such margin is intraspecifically variable. For example, within *Corythosaurus casuarius*, it is straight in TMP 80.40.1 but slightly curved in ROM 1933; or within *Lambeosaurus lambei*, the margin is straight in TMP 81.37.1 but gently curved in ROM 1218.

Cruzado-Caballero et al. [12], [71] based the taxonomic distinction of *Arenysaurus ardevoli* and *Blasisaurus canudoi* on a number of dental, dentary, and jugal characters. However, as we show below, none of those characters is informative for taxonomic distinction of these taxa.

Dental characters consisted of: absence of accessory ridges on the dentary teeth of *B. canudoi* but present in *A. ardevoli*; and height/width ratio of dentary tooth crowns being as high as 3.65 in *B. canudoi* but only 3.15 in *A. ardevoli*. Accessory ridges are commonly occurring on the enameled surface of dentary tooth crowns of lambeosaurines, where they are found accompanying the median primary ridge [1]. According to our observations of the teeth in numerous lambeosaurine specimens from well-sampled taxa like *Lambeosaurus lambei*, *Corythosaurus casuarius*, and *C. intermedius*, when accessory ridges are present, these occur among all individuals within a species (however not necessarily in all teeth within a dental battery). More specifically, the number, apicobasal length, prominence, and distribution within a single dental battery of these accessory ridges vary intraspecifically [2], [50]. For example, within *L. lambei*, CMN 8633 shows teeth lacking accessory ridges and others with one fine long accessory ridge, whereas CMN 2869 shows two rather prominent accessory ridges in nearly all teeth that do not reach the apical region of the crown. In *C. casuarius*, many but not all dentary teeth of ROM 870 show two relatively well developed and apicobasally continuous accessory ridges, one at each side of the primary carina; ROM 868 shows only one accessory ridge in many teeth, and this is finer than in ROM 870; and ROM 1933 displays teeth with one accessory ridge that is more prominent than in ROM 870. A similar variation is also found in specimens of *C. intermedius*. In all these taxa, at least one specimen (e.g., ROM 871 for *C. casuarius*, ROM 776 for *C. intermedius*, and ROM 794 for *L. lambei*) shows teeth lacking accessory ridges and teeth with less pervasive ridges than in the other specimens known for the species, ridges that do not extend along the entire apicobasal length of the tooth crowns and show a very low relief. This variable intraspecific distribution pattern of accessory ridges indicates that the lack of observed accessory ridges in *B. canudoi* does not allow, in itself, distinction of this species from *A. ardevoli*. This is particularly true in the case of MPZ 99/665 because only a relatively small section of the enameled surface of tooth crowns, adjacent to the occlusal plane of the dental battery, is exposed; most of the lingual surface of the dental battery is heavily damaged and poorly preserved (Fig. 13A). Accessory ridges might well be present in *B. canudoi* but given the limited exposure of well-preserved tooth crowns in the specimen, they might remain unnoticed if the ridges occurred in a fraction of the teeth, had a lower relief, and did not extend along the entire apicobasal height of the crown.

Regarding the height/width ratio of tooth crowns, the few teeth that appear to show completely preserved crowns in the dentary of *A. ardevoli* are not entirely exposed (Fig. 13D). Thus, the height/width ratio of these tooth crowns would actually be greater than 3.15 if it could be measured on the completely exposed teeth, becoming closer (although probably not as high) to the 3.65 ratio of *B. canudoi* and, likely and more importantly, beyond the 3.3 cut value for state 3 of character 4 in Prieto-Márquez [2]. It must be noted also that the preserved dentary teeth in *A. ardevoli* occupy a position slightly caudal to the mid-length of the dental battery, not mesial as indicated by Cruzado-Caballero et al. [71] when comparing to the mesial teeth of *B. canudoi*. Furthermore, the height/width ratio of tooth crowns may be greater in more mesial crowns when compared to that of more distal teeth; for example, in *Corythosaurus intermedius* ROM 776, the ratio ranges from 2.9 at the distal end of the dental battery to 3.5 near the mesial end. Therefore, we do not consider the height/width ratios of dentary teeth in the two Blasi lambeosaurine species as distinctive characters.

Dentary characters supporting the distinction of the two lambeosaurine species from Blasi, according to Cruzado-Caballero et al. [12], [71], included the following: in dorsal view, the dentary of *A. ardevoli* is labiolingually concave (probably meaning arcuate or ‘bowed’ along the alveolar margin), whereas that of *B. canudoi* is rather straight; distance between the first tooth position and the inflexion point of the symphysis being about 15.5% of the total length of the dental battery in *B. canudoi*, but only 4.5% in *A. ardevoli*; convex dorsal margin of the coronoid process of *B. canudoi*, while being tip-like in *A. ardevoli*; coronoid process further inclined rostrally in *B. canudoi* than in *A. ardevoli*; and dental battery of *A. ardevoli* containing 37 tooth positions, versus the 35 of *B. canudoi*. However, all these characters are either intraspecifically variable or based on inaccurate observations. More specifically, two observations cast doubt on the possibility of distinguishing the dentaries of *A. ardevoli* and *B. canudoi* on the basis of the curved or straight alveolar margins. While the alveolar margin of the dentary of *B. canudoi* is indeed straight, that of *A. ardevoli* is also straight throughout the rostral and middle thirds of MPZ 2008/258. The caudal third of the alveolar margin in MPZ 2008/258 appears curved laterally in dorsal view. However, this apparent curvature is most likely an artifact of the erosion of the alveolar ridges and sulci observed along the caudal third of the dental battery. Furthermore, except for a handful of preserved teeth, the dental battery of *A. ardevoli* is missing its teeth, whereas that of *B. canudoi* preserves all its dentition in place. When viewed dorsally, a tooth-bearing dental battery contributes to a more straight orientation of the dentary alveolar margin; in contrast, a tooth-less dentary appears to have a curved alveolar margin as the alveolar ridges and sulci become less prominent rostrally and, specially, caudally.

As shown in Fig. 13A and F, the origin of the ventral deflection of the symphyseal process character occurs near the mid-length of the dental battery in both *A. ardevoli* and *B. canudoi* (contra [12], [71]). This condition was quantified as in character 37 of Prieto-Márquez [2], i.e., as the ratio between the distance from the caudal margin of the coronoid process to the inflexion point of the ventral margin and the distance from the caudal margin of the coronoid process to the rostralmost alveolus of the dental battery. Following this measure, the resulting ratio for *A. ardevoli* was 0.57, a value close to the 0.54 obtained for *B. canudoi*; both values fall within state 2 of this character [2].

The dorsal margin of the apex of the coronoid process is incompletely preserved in *B. canudoi*, particularly the caudal edge that is dorsally projected in *A. ardevoli* to form a sharp tip (broken off from MPZ 2008/258 at the time that the photograph shown in Fig. 13F was taken); therefore, the claim that the dorsal margin of the apex in *B. canudoi* lacked a dorsal projection is unsubstantiated. The extent of rostral inclination of the coronoid process in lambeosaurines (and in hadrosaurids in general) varies intraspecifically. For example, within *Lambeosaurus lambei*, the angle formed by the long axis of the coronoid process and the tooth row ranges from 67° in YPM 3222 to 78° in AMNH 5373. The difference in rostral inclination between the coronoid processes of *B. canudoi* (75°; see Fig. 13A) and *A. ardevoli* (78°; see Fig. 13F) falls within the expected range of variation for a lambeosaurine species, and thus, has no diagnostic value.

The dentary of *Arenysaurus ardevoli* is approximately 30% longer than that of *Blasisaurus canudoi* (445 mm [11] vs 340 mm [12]). Thus, *A. ardevoli* is expected to show more dentary teeth than *B. canudoi* based only of the size difference. Furthermore, the number of alveoli varies to some extent even among individuals of comparable size within lambeosaurine species. For example, the number of dentary alveolar positions in adults (i.e., skull length at least 85% of the maximum skull length observed for the species) of *Lambeosaurus lambei* ranges from as low as 36 in ROM 1218 and 37 in CMN 351 to as many as 42 in ROM 794. Thus, a minor difference of two tooth positions between two specimens lacks diagnostic value.

Finally, the four jugal characters regarded by Cruzado-Caballero et al. [71] as distinctive between *Arenysaurus ardevoli* and *Blasisaurus canudoi* consisted of: caudodorsal margin of the rostral process being straight in *A. ardevoli*, unlike in *B. canudoi*; the maxillary process being projected less laterally and more rostrally in *B. canudoi* than in *A. ardevoli*; the postorbital ramus of *B. canudoi* being more caudodorsal relative to the longitudinal axis of the jugal (60°) than that of *A. ardevoli* (45°); and V-shaped orbital fenestra in *A. ardevoli*, unlike that of *B. canudoi*. Again, these characters have either no diagnostic value or are based on inaccurate observations of the specimens. Specifically, and contrary to Cruzado-Caballero et al. [71], the caudodorsal margin of the rostral process in *B. canudoi* is actually straight (see Fig. 13C). Furthermore, the orientation of the caudodorsal margin of the rostral process varies intraspecifically; for example, within *Corythosaurus intermedius*, this margin is strongly curved in CMN 8703 and ROM 777, but shows only a very gently curvature in ROM 845. The maxillary process, on the medial articular surface of the rostral process, is heavily eroded in the partial jugal of *A. ardevoli* (Fig. 14B; see also [66]: fig. 4.83A); thus, its orientation cannot be ascertained, precluding comparison with the nearly complete process preserved in *B. canudoi*. Likewise, the angle between the postorbital ramus and the longitudinal axis of the jugal of *A. ardevoli* cannot be measured with any degree of accuracy. This is because the entire ventral region, infratemporal margin, and ventral and quadratojugal flanges of the jugal of *A. ardevoli* are missing (Fig. 14; see also Cruzado-Caballero, 2012: fig. 4.83A), so that no longitudinal axis can be confidently set in the specimen. Finally, it must be noted that in hadrosaurids the jugal contributes to slightly less than the ventral half of the orbital fenestra, which Cruzado-Caballero et al. [71] referred to for distinguishing *A. ardevoli* and *B. canudoi*. Thus, based solely on the morphology of the jugal, the geometry of the orbital fenestra remains unknown. Regardless, the outline of the orbital margin in lambeosaurines lacks diagnostic value because it is too variable intraspecifically. For example, within *Corythosaurus intermedius*, the ventral margin of the orbital fenestra (as it is defined by the jugal) shows a wide asymmetrical U-shape contour in CMN 8703 and ROM 845, whereas it displays a narrower, more asymmetrical, and V-shape contour in CMN 8704 and ROM 777.

Despite the lack of diagnostic utility of the characters discussed above, the available osteological data neither support nor invalidate a possible synonymy of *Arenysaurus ardevoli* and *Blasisaurus canudoi*. Notably, the diagnostic characters of these species occur in non-overlapping elements, i.e., the frontal in *A. ardevoli* and the jugal in *B. canudoi*. Therefore, although *B. canudoi* might represent a junior synonym of *A. ardevoli*, we provisionally maintain their taxonomic separation pending the finding of sufficiently complete diagnostic overlapping elements and/or more conclusive data in this regard, as this choice allows for future testing of the validity of the two lambeosaurines from Blasi (e.g., via phylogenetic analysis). As a matter of fact, this conclusion is also applicable to the distinction of the two Blasi taxa and *Pararhabdodon isonensis* because there are no overlapping taxonomically informative elements among these three lambeosaurine species.

### Parasaurolophini Parks, 1922 [59]

#### Definition

*Parasaurolophus walkeri* Parks, 1922 [59] and all lambeosaurine taxa more closely related to it than to *Lambeosaurus lambei* Parks, 1923 [40], *Tsintaosaurus spinorhinus* Young, 1958 [44], or *Aralosaurus tuberiferus* Rozhdestvensky, 1968 [41].

#### Diagnosis

Lambeosaurine hadrosaurids possessing jugal with orbital margin wider than infratemporal margin and concomitant constricted ventral margin of infratemporal fenestra; nasal articulation surface of frontal shaped into dorsoventrally thickened, tongue-like platform that projects caudodorsally to overhang the parietal in adults; rostrocaudally shortened ectocranial surface of frontal, with length/width ratio less than 0.4; ilium with short postacetabular process, ratio between length of postacetabular process and length of the central plate up to 0.8; ischium with ratio between length of the ischial shaft and length of the distal ventral expansion of 0.25 or greater; and long axis of ‘boot-like’ distal process rostroventrally directed, inclination starting at dorsal margin of process.

#### Type genus

*Parasaurolophus* Parks, 1922 [59].

#### Comments

The *Parasaurolophus*-clade has been previously referred to as Parasaurolophini in the literature [12], [64], [66], [67], [69]. However, so far no formal definition and diagnosis have been provided. Here, we amend this shortcoming by proposing the definition and diagnosis of Parasaurolophini provided above.

### New lambeosaurine specimens from the upper Maastrichtian of the eastern Tremp Syncline (southern Pyrenees, northeastern Spain)

#### Serrat del Corb locality

**Figure 15:**
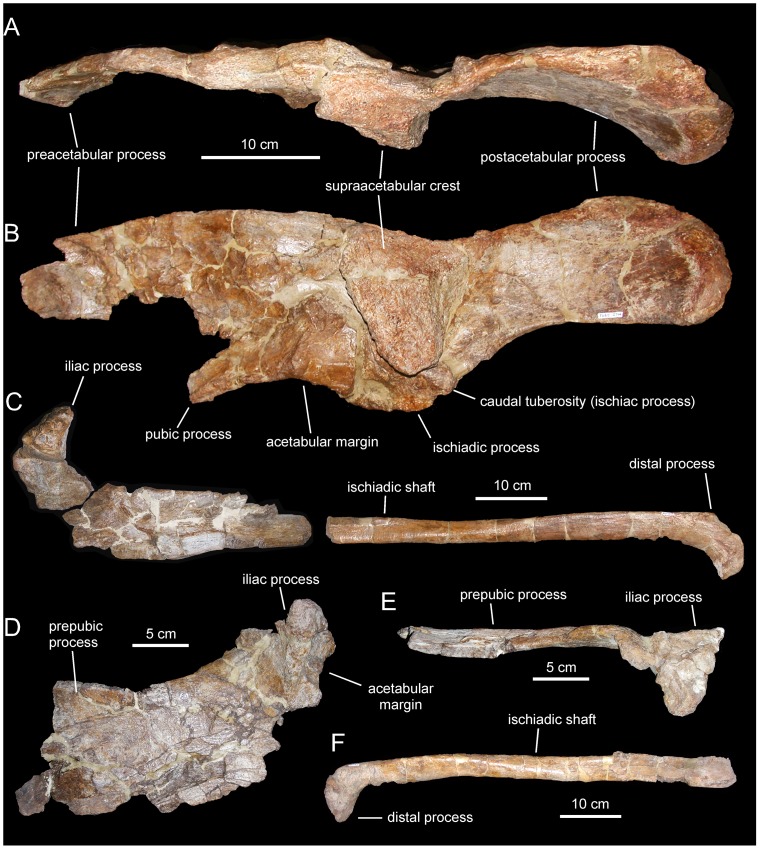
Indeterminate lambeosaurine specimen from Serrat del Corb. A. Left ilium (MCD 4791) in dorsal view. B. Lateral view of same. C. Left ischium (MCD 4787) in lateral view. D. Left pubis (MCD 5088) in dorsal view. E. Lateral view of same. F. Right ischial shaft (MCD 4788) in lateral view.

A nearly complete pelvis (left ilium, MCD 4791; ischia, MCD 4787 and 4788; and partial left pubis, MCD 5088; Fig. 15 and table 4) was found in this site associated to four sacral or proximal caudal vertebrae [72].

**Table 4:** Selected measurements (in mm) of the Serrat del Corb lambeosaurine.

Element	Measurement
Ilium (MCD 4791), total length (incomplete preacetabular process)	618
Ilium (MCD 4971), length of postacetabular process	218
Ilium (MCD 4791), length of central plane, from ventral margin of caudal tuberosity of ischiadic process to rostroventral end of (incomplete) pubic process	241
Ilium (MCD 4791), height of central place, from ventral margin of ischiadic process to highest point along convex dorsal margin	173
Ilium (MCD 4791), craniocaudal length of supraacetabular crest	112
Ilium (MCD 4971), dorsoventral extent of supraacetabular crest from lowest point of concave dorsal margin to ventral apex	111
Ischium (MCD 4787), total length	863
Ischium (MCD 4787), maximum depth of ischiadic shaft	41
Ischium (MCD 4787), maximum depth of distal process (incomplete)	95
Ischium (MCD 4788), total length (missing proximal region)	701
Ischium (MCD 4788), maximum depth of ischiadic shaft	44
Ischium (MCD 4788), maximum depth of distal process (incomplete)	97
Pubis (MCD 5088), total length (incomplete prepubic process)	267
Pubis (MCD 5088), maximum depth of proximal constriction (incomplete)	107

#### Ilium

MCD 4971 lacks the distal half of preacetabular process and the craniodorsal margin of the pubic process (Fig. 15A and B). The proximal segment of the preacetabular process is mediolaterally compressed and its lateral surface is slightly concave. The medial surface of the process shows longitudinal grooves and a medial ridge for attachment to the sacrum.

The central plate of the ilium contains the supraacetabular crest dorsally and the acetabular margin ventrally, which is formed by the pubic and ischiadic process. The dorsal margin shows a sinuous lateral profile, being gently convex above the proximal region of the preacetabular process and concave immediately caudal to the supraacetabular crest. A thin median low ridge extends longitudinally along most of the narrow dorsal surface of the central plate. The pubic process is triangular in lateral profile, as in other hadrosaurids, and mediolaterally expanded along its ventral acetabular margin. The ischiadic process is composed of two protrusions, as is characteristic of hadrosaurids, although these are heavily eroded. A prominent oblique ridge extends craniodorsally from the caudoventral margin of the posterior protrusion of the ischiadic process. The supraacetabular crest displays an asymmetrical V-shaped lateral profile, its apex slightly oriented caudoventrally. The supraacetabular crest projects ventrally beyond the level of the lateral ridge of the ischial process, extending along 80% of the dorsoventral depth of the central iliac plate. As is commonly seen in hadrosaurids, the apex of the supraacetabular crest is found cranial to the ventral margin of the caudal protrusion of the ischiadic process.

The postacetabular process is nearly as long as the central plate of the ilium, and projects caudodorsally from the caudal region of the central plate. It is mediolaterally compressed and slightly twisted medioventrally, and its lateral surface is axially concave. The medial side of the postacetabular process has an oblique, wide, but low ridge that crosses the process from its proximoventral margin, near the ischial process, to the dorsodistal border. Ventral to this ridge, the medial surface of the process faces caudoventrally as well as medially, whereas the surface above the ridge faces medially. Distally, the postacetabular process becomes dosoventrally expanded, more so dorsally than ventrally. The distal margin is arcuate and shows a convex dorsal expansion.

#### Ischium

This left ischium (MCD 4787; Fig. 15C) lacks the pubic process, the acetabular margin, and the ventral border of the proximal region that would contain the obturator process and notch; the right ischium (MCD 4788; Fig. 15H) is missing the entire proximal region. In MCD 4787, the caudodorsal margin of the iliac process is caudally recurved, ‘thumb-like’ in lateral profile, and greatly expanded mediolaterally. The articular surface is carved by a reticular pattern of grooves and ridges. The caudal portion of the proximal region of the ischium is strongly compressed mediolaterally, but this may have been enhanced by postdepositional compression of the specimen, as evidenced by the presence of fissures and distorted areas located on the lateral surface of the bone.

The shaft of these ischia shows a flat articular medial surface that contains numerous longitudinal striations. In cross section, the proximal half of the shaft is subrectangular deeper than wide. In contrast, the distal half of the shaft shows a well-defined triangular cross section. The distal end of the shaft is ventrally expanded to form the “foot-like” process seen in all lambeosaurines [1]. The caudodorsal region of this process is eroded in both ischia. As preserved, the dorsoventral length of the process is slightly greater than twice the dorsoventral width of the shaft. The distal process of the ischium is mediolaterally compressed and its lateral surface is slightly concave dorsoventrally.

#### Pubis

The left pubis (MCD 5088; Fig. 15D and E) is represented by the iliac process and part of the prepubic process. The iliac process is tetrahedral and caudolaterally oriented. The dorsal, lateral, and caudal surfaces of the process are triangular. The caudal surface is concave. The lateral and medial corners of the caudodorsal margin of the process are very prominent. The caudolateral and caudomedial margins form two thick ridges. The prepubic process is a thick bony lamina displaying a strongly concave lateral profile of its dorsal margin. The preserved portion of the prepubic process includes a relative deep proximal constriction.

#### Comments

Referral of this pelvic girdle to Lambeosaurinae is supported by two unambiguous synapomorphies [2]: well developed, ‘thumb-like’ caudal curvature of the caudodorsal corner of the iliac process and presence of a ‘boot-like’, ventrally expanded distal process of the ischium. At a more inclusive level, this individual also shows various hadrosaurid synapomorphies [2], [58]: ventral apex of the supraacetabular crest located craniodorsally relative to the caudoventral margin of the lateral ridge of the caudal protuberance of the ischiadic process; craniocaudally short supraacetabular crest, ratio between the breadth of the crest across its dorsal region and the craniocaudal length of the central iliac blade less than 0.55; and lateral margin of the iliac process progressively disappearing ventrally into the lateral surface of the region adjacent to the acetabular margin. No apomorphies were recognized in the specimen that would allow referral to a taxonomic level lower than Lambeosaurinae.

### Molí del Baró 1 Locality

#### Ischium

**Figure 16:**
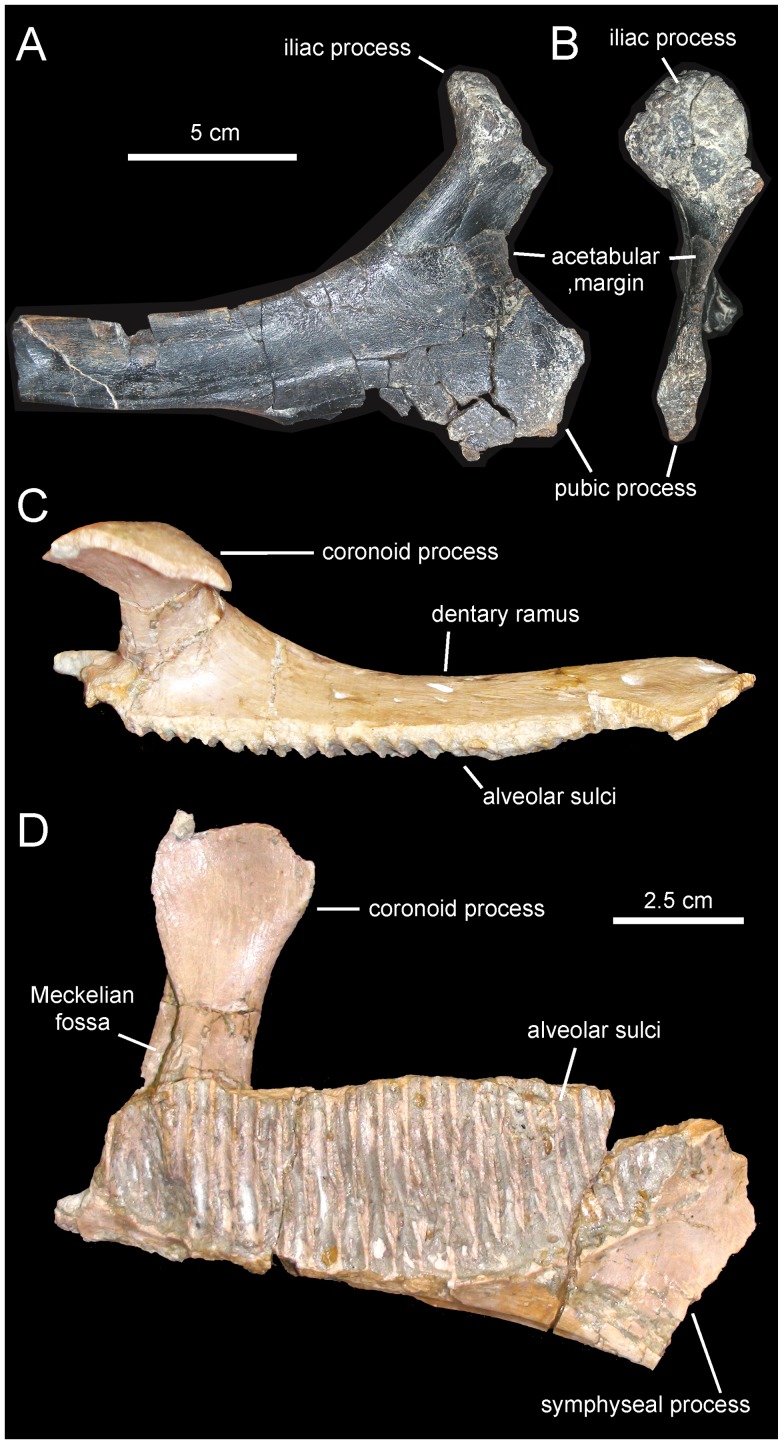
Indeterminate lambeosaurine specimens from Molí del Baró 1 and Barranc de Torrebilles 4. A. Right ischium (MCD 5089) from Molí del Baró 1 in lateral view. B. Cranial view of same. C. Left dentary (MCD 5059) from the Barranc de Torrebilles 4 locality in dorsal view. D. Medial view of same.

MCD 5089 is represented by the proximal region and proximal extent of the shaft of a right ischium (Fig. 16A and B). The fragment measures 203 mm in length from the caudal end of its incomplete shaft to the cranial margin of the pubic process. The caudodorsal margin of the iliac process is recurved caudally. The teardrop-shaped articular facet of the iliac process is mediolaterally expanded and exhibits a pitted surface. Ventral to the iliac process, the cranial region of the ischium adjacent to the acetabular margin is mediolaterally compressed and forms a thin lamina. This lamina is ventrally continuous with the pubic process. The latter is mediolaterally compressed and subrectangular, becoming slightly thicker towards its articular margin. The caudoventral margin of the proximal region of the ischium is incompletely preserved. The lateral surface of the pubic process is gently concave, whereas the medial one is strongly depressed near the articular margin. Caudal to the pubic process, along the ventral margin of the proximal region of the ischium, the obturator notch and process are only partially preserved. The proximal region of the obturator process is broad, thick, and medioventrally oriented. A ridge extends ventrally from the caudal margin of the obturator process obliquely into the mediodorsal region of the shaft. The preserved segment of the ischiadic shaft is subtriangular in cross section and mediolaterally wider dorsally than ventrally. Its lateral side is strongly concave dorsoventrally, whereas the medial surface is flat proximally and dorsoventrally convex further distally.

This ischium is referable to Lambeosaurinae because of the well-developed curvature in the caudodorsal corner of the distal margin of the iliac process of the ischium, a synapomorphy of this clade of hadrosaurids [2]. In the Molí del Baró 1 ischium the articular facet of the iliac process is less expanded mediolaterally than in the Serrat del Corb ischium described above. However, this difference may be ontogenetic rather than taxonomic, given the substantial size difference existing between these ischia and the observation that juvenile ischia show proportionately narrower articular facets of the iliac process [51].

### Barranc de Torrebilles 4 locality

#### Dentary

This specimen (MCD 5059, Fig. 16C and D) is represented by an incomplete left dentary. The dentary lacks the edentulous region and most of the symphyseal process, all the teeth, the ventral margin of its caudal third along the Meckelian groove, and the dorsal border of the apex of the coronoid process. The bone is 111 mm in length and its medial surface displays 24 alveoli. Each alveolus is narrow, bounded by thin vertical ridges, and slightly tilted caudally. The ventral deflection of the rostral edentulous region originates slightly caudal to the mid-length of the dental battery. The preserved ventral margin of the proximal region of the symphyseal process forms an angle of 20° with the dorsal margin of the alveolar region of the dentary. However, the actual deflection angle along the distal border of the symphyseal process might have been greater. In dorsal view, the dorsal margin of the alveolar region is parallel to the lateral surface of the dentary. The coronoid process is, from its ventral base to its dorsal apex, taller than twice the depth of the deepest region of the alveolar region of the dentary. The long axis of the coronoid process is slightly inclined rostrally, forming a 79-degree angle with the dorsal margin of the alveoli. The dorsal region of the coronoid process is rostrocaudally expanded, more so rostrally than caudally.

We consider MCD 5059 a saurolophid hadrosaurid based on the dorsal alveolar surface being parallel to the lateral side of dentary and the caudal edge of dental battery being located caudal to the coronoid process [2]. Furthermore, the specimen can be referred to Lambeosaurinae because of the presence of a strong ventral deflection of the symphyseal process that originates near the mid-length of the dental battery. Within hadrosaurids, the co-occurrence of such strong deflection and its relatively caudal point of origin can be observed in the lambeosaurines *Amurosaurus riabinini* (e.g., AEHM 1/12), *Sahaliyania elunchunorum* [57], and *Tsintaosaurus spinorhinus* (e.g., IVPP [Institute of Vertebrate Paleontology and Paleoanthropology, Beijing, China] V723). Outside Hadrosauridae, these two conditions are also present in *Protohadros byrdi* (e.g., SMU [Shuler Museum of Paleontology, Shouthern Methodist University, Dallas, USA] 74582).

### Review of Other European Material Previously Referred or Referrable to Lambeosaurinae

#### Additional material from the Blasi localities

The Blasi 1–5 localities (Fig. 1) have yielded a relatively rich and well preserved fossil record of late Maastrichtian hadrosaurid dinosaurs [11], [21], [55], [73], [74]. Most remarkable among these are the types and referred materials of *Arenysaurus ardevoli* [11], [71] (Fig. 13D-F) and *Blasisaurus canudoi* [12], [71] (Fig. 13A–C; see results section above).

#### Blasi 3

**Figure 17:**
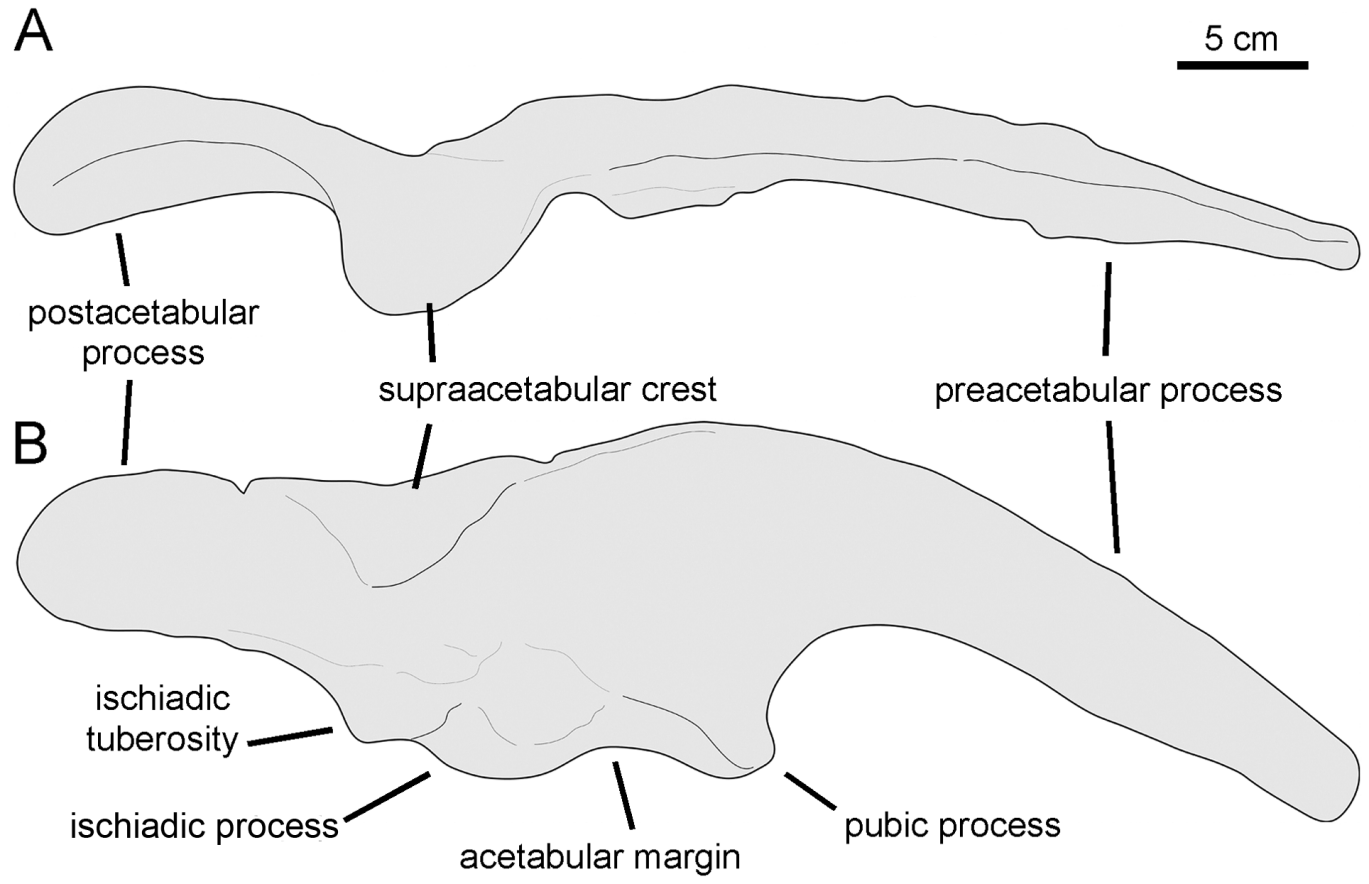
Indeterminate lambeosaurine ilium from Blasi 3 . A. Dorsal view of a right ilium (MPZ 2005/90) recovered from the Blasi 3 locality. B. Lateral view of same. Drawings based on the photographs in Cruzado-Caballero et al. ([73]: fig. 2).

Cruzado-Caballero et al. [73], [74] referred an isolated ilium (MPZ 2005/90; Fig. 17) to Lambeosaurinae on the basis of an arcuate ventrally deflected preacetabular process, relatively deep proportions and an elongate pendant supraacetabular crest. However, the angle of ventral deflection of the preacetabular process is pronounced (30° or more from the horizontal) in all hadrosaurids and many non-hadrosaurid hadrosaurs [12], [14]. The length/depth ratio was calculated by Cruzado-Caballero et al. [73] using the total length of the ilium; however, using the total length of the ilium reduces the sample of specimens that can be used to explore the variation of this bone, since in many exemplars the cranial end of the preacetabular process is incomplete. Based only on the central plate, a relatively deep ilium (length/depth ratio greater than 0.8) is present, among hadrosaurids, in all lambeosaurines except *Parasaurolophus cyrtocristatus*, and in the saurolophine *Brachylophosaurus canadensis* [2]. Extension of the supraacetabular crest ventral to the middle of the iliac central plate is a synapomorphy of Hadrosauridae (sensu [2]); outside Hadrosauridae, it occurs also in *Tethyshadros insularis* (see Dalla Vecchia 2009c). Additionally, MPZ 2005/90 shows the following unambiguous hadrosaurid synapomorphies according to Prieto-Márquez [2]: ilium with ventral-most margin of the supraacetabular crest located craniodorsally relative to the caudoventral margin of the caudal protuberance of the ischiadic process; short supraacetabular crest of the ilium, ratio between the craniocaudal breadth of the process across its dorsal region and the craniocaudal length of the central iliac blade less than 0.55; and lateral margin of the iliac process progressively disappearing ventrally into the lateral surface of the region adjacent to the acetabular margin. Therefore this ilium is unambiguously referable to Hadrosauridae. In addition, we concur with Cruzado-Caballero et al. (2005) in that MPZ 2005/90 probably belonged to a lambeosaurine; however, we base this conclusion solely on the relatively deep iliac plate of the specimen, given the rare occurrence of lambeosaurines with shallow iliac plates and saurolophines with deep plates.

#### Blasi 4

A fragment of rostral process of a left jugal (MPZ 2007/1884) recovered from this locality has recently been referred to Lambeosaurinae by Cruzado-Caballero et al. [9], [75]. These authors based their referral on the strong similarity that exists between the morphology of MPZ 2007/1884 and the holotype specimen of *Blasisaurus canudoi* (MPZ99/667, the jugal collected form Blasi 1). We agree with Cruzado-Caballero et al. [9], [75] in referring the element from Blasi 4 to Lambeosaurinae because it shows a ventrally projected, triangular, and narrow caudoventral margin of the rostral process of the jugal, at least twice as deep as it is wide, a synapomorphy for the clade [2].

#### Blasi 5

**Figure 18:**
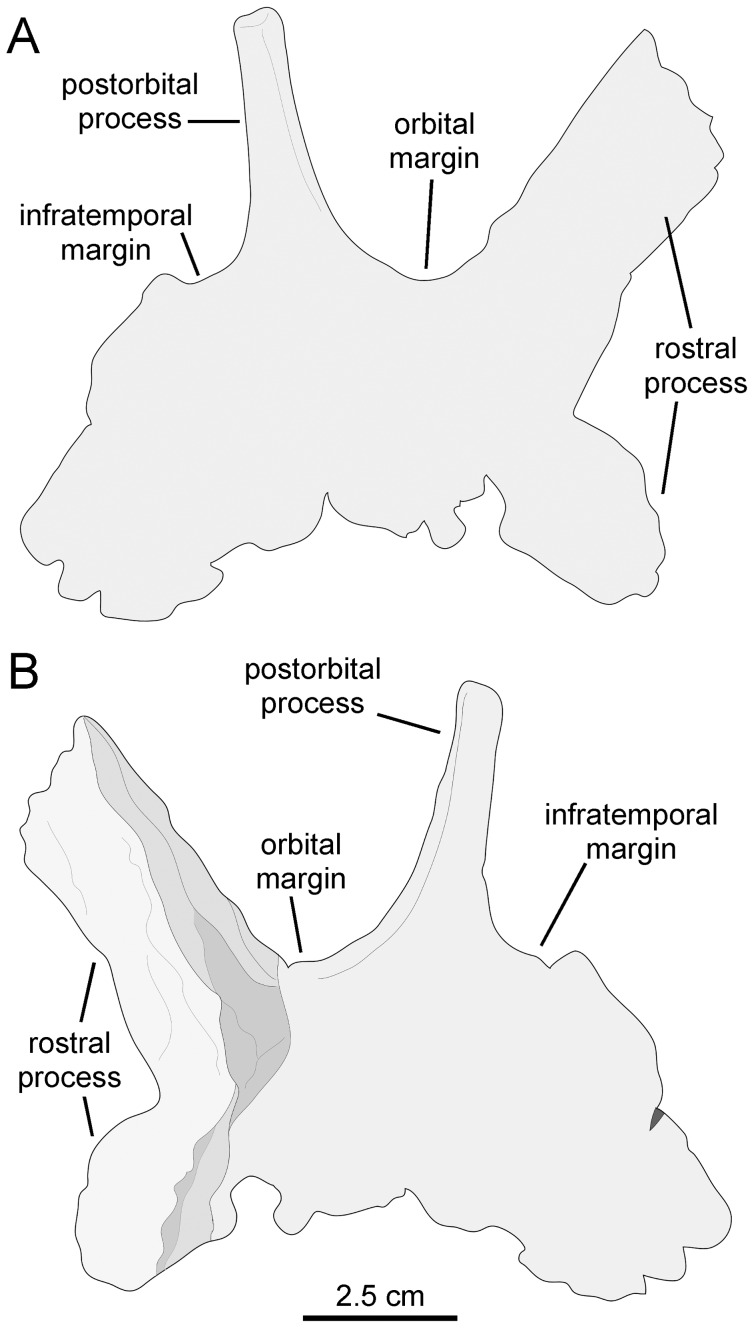
Indeterminate lambeosaurine jugal Blasi 5. A. Partial right jugal (MPZ 2007/1885) in lateral view. B. Medial view of same. Drawn from Cruzado-Caballero et al. ([9]: fig. 2).

Cruzado-Caballero et al ([9]: figs. 2.3 and 2.6) referred a partial jugal, MPZ 2007/1885 (Fig. 18), to Hadrosaurinae (equivalent to the clade Saurolophinae to the exclusion of *Hadrosaurus foulkii*, following [2]). According to these authors, this jugal belonged to a saurolophine hadrosaurid on the basis of an asymmetrical rostral process that is dorsoventrally expanded along the maxillary and lacrimal articular facets, a broad orbital constriction, and a vertical postorbital process. The asymmetry of the rostral jugal process in saurolophines occurs when the caudoventral spur lies ventral to the level of the caudodorsal corner of the lacrimal process. This asymmetry typically characterizes saurolophines, except Brachylophosaurini [2]. However, the rostral process of MPZ 2007/1885 is too incomplete to ascertain its geometry: the central rostral region is missing, and the caudodorsal margin of the lacrimal process and caudoventral spur are incompletely preserved (Fig. 18). The proportionately great dorsoventral depth of the orbital constriction in the Blasi 5 jugal does not characterize saurolophine hadrosaurids. Indeed, a proportionately deep orbital constriction occurs in some saurolophines (e.g., *Saurolophus osborni*, CMN 8796; *Prosaurolophus maximus*, CMN 2870 and 2777) and some lambeosaurines (e.g., *Amurosaurus riabinini*, AEHM 1/112, and notably *Sahaliyania elunchunorum* [57]: fig. 4A). Finally, if we consider as reference a long axis of the jugal that unites the ventral-most points of the orbital and infratemporal margins, nearly vertically oriented postorbital processes are found in lambeosaurines (e.g., cf. *Tsintaosaurus spinorhinus*, IVPP V830; *Olorotitan ararhensis* [6]: fig. 4; *Hypacrosaurus altispinus*, CMN 8673) and saurolophines (e.g., *Naashoibitosaurus ostromi*, NMMNH P16106; *Gryposaurus notabilis*, ROM 873). Intraspecifically, the orientation of the postorbital process of the jugal may show substantial variation, at least in some species. Such is the case of the saurolophine *Maiasaura peeblesorum*: the process of TCMI (The Children’s Museum of Indianapolis, Indianapolis, USA) 2001.89.2 is strongly inclined caudally, forming a 36-degree angle with the long axis of the jugal; in contrast, this angle is as high as 71° in ROM 44770. Therefore, MPZ 2007/1885 lacks characters allowing referral of the specimen to Saurolophinae. Interestingly, however, the preserved orbital and infratemporal margins in the Blasi 5 jugal allow assessing that its length/width proportions are comparable to that of the rostrocaudally short jugals of lambeosaurines like *B. canudoi* and cf. *T. spinorhinus* (IVPP V830), which also show relatively deep orbital constrictions. This suggests that MPZ 2007/1885 is more likely the jugal of an indeterminate lambeosaurine hadrosaurid.

### Moror Locality

**Figure 19:**
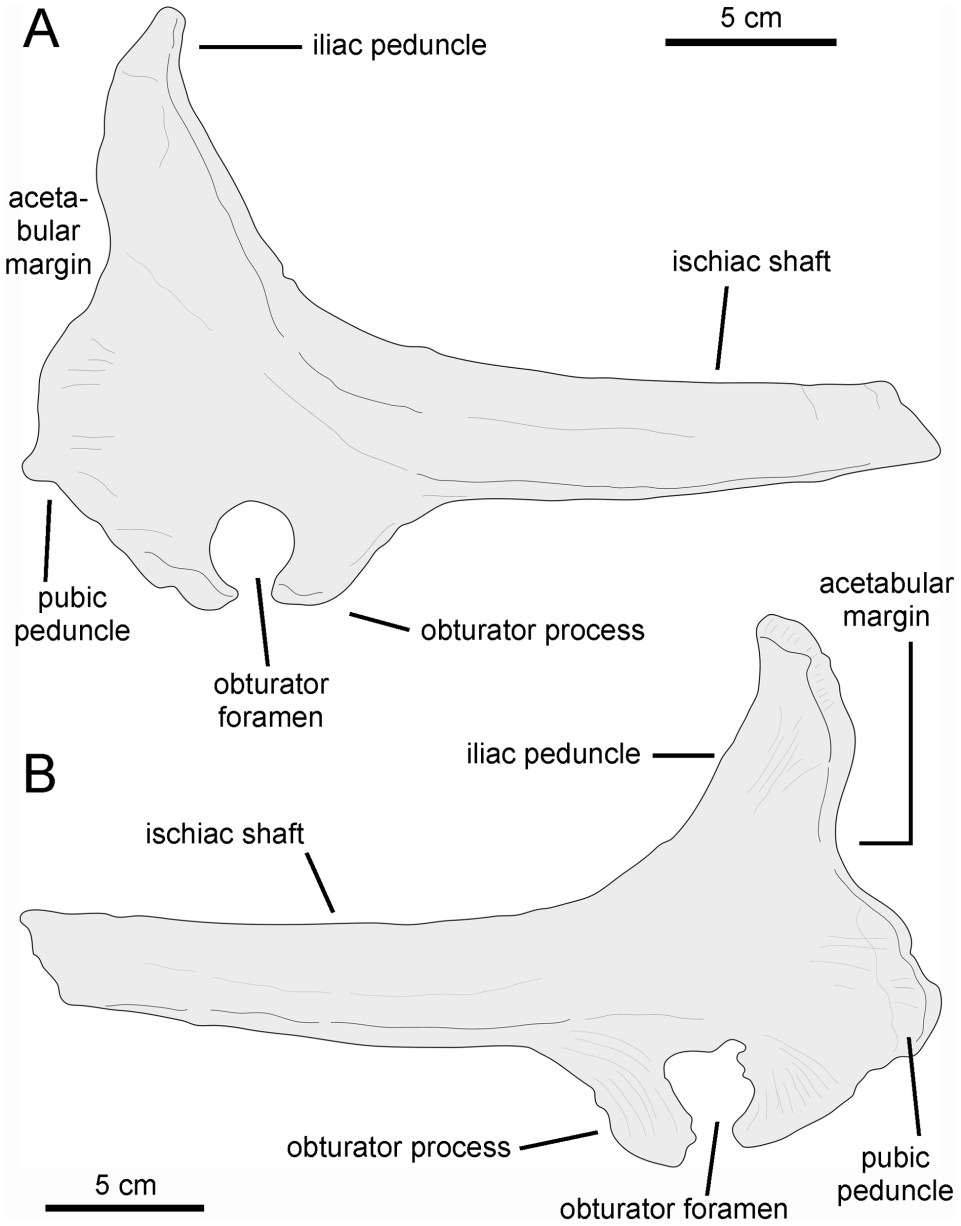
Indeterminate lambeosaurine ischia from Moror . A. Left ischium (IPFUB unnumbered specimen) in lateral view. B. Right ischium (IPFUB unnumbered specimen) in lateral view. Drawings based on the photographs in Brinkmann ([76]: fig. 2 and 3).

Brinkmann [76] described and figured two ischia (Fig. 19) found near the village of Moror (misspelled as Moró in the literature, e.g. [77]) just west to the Noguera Pallaresa River, south of the town of Tremp (south-central Tremp Syncline, Lleida province, northeastern Spain; Fig. 1). According to the indications given by Brinkmann ([76]: fig. 1) and data from the geological map of Catalonia (Llimiana Sheet [31]), the fossils were found in the Maastrichtian ‘grey unit’ of the Tremp Formation. Although Brinkmann [76] recognized the hadrosaurian nature of these ischia, he was uncertain regarding their affinities within the clade. Later, Casanovas et al. [77] found the morphology of these ischia consistent with that of lambeosaurines because “they are massively constructed and bear conspicuous peduncles, as is the case in *Hypacrosaurus* and *Parasaurolophus*” (Casanovas et al. [77]: p. 280). The ischia consist of two nearly complete proximal regions with the proximal segments of their shafts (one left and one right, IPFUB [Institut für Paläontologie, Freie Universität Berlin, Germany] unnumbered specimens), probably belonging to the same individual [76]. In both ischia, the iliac process is complete and shows a caudally recurved dorsal margin, with its ‘thumb-like’ lateral profile diagnostic of lambeosaurines [2], [56] that supports Casanovas et al. [77] referral.

### Les Llaus Locality

**Figure 20:**
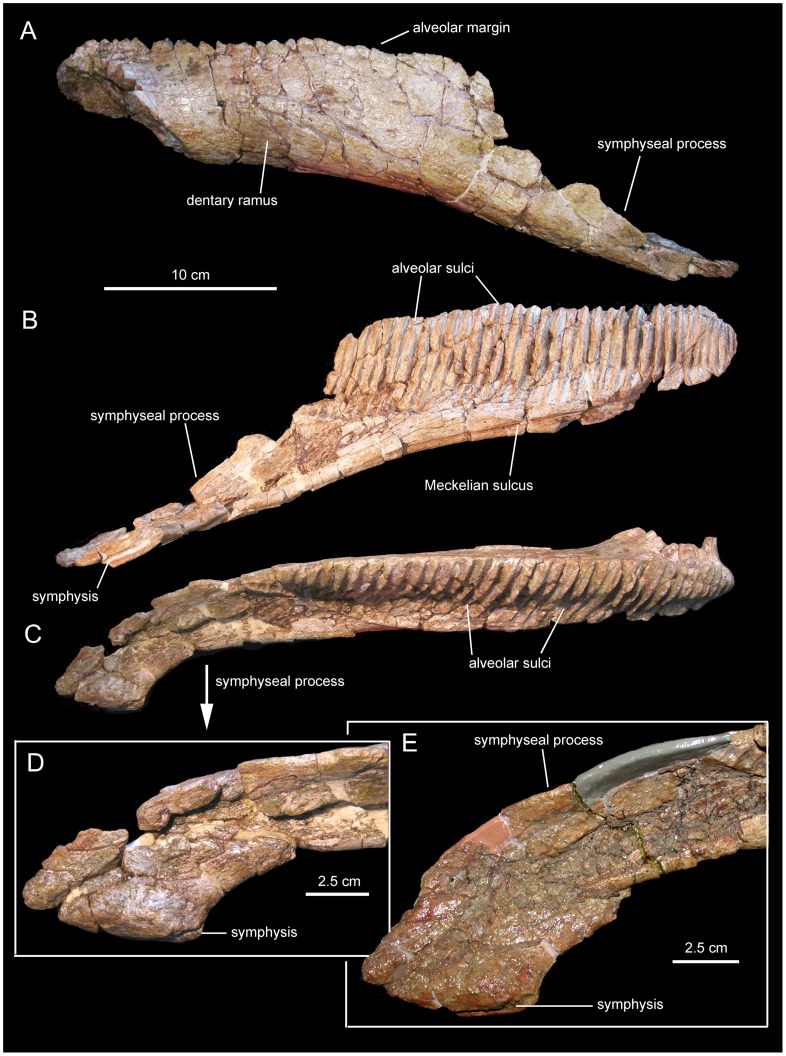
Indeterminate lambeosaurine dentary, IPS 29920. A. Lateral view. B. Medial view. C. Dorsal view. D. Detail of the symphyseal process in dorsal view, as it is currently prepared. E. Detail of the symphyseal process in dorsal view, showing its artificial greater lingual elongation caused by the former (pre-2008) preparation of the specimen.

Casanovas et al. [77] referred to *Pararhabdodon isonensis* IPS 29920 (formerly IPS SRA 27), an edentulous partial dentary (Fig. 20) collected at Les Llaus locality [78], [79]. This locality lies north of the town of Sant Romà d’Abella, Lleida Province, in the northeastern Tremp Syncline, northeastern Spain (Fig. 1). The upper Maastrichtian strata that yielded IPS 29920 are in the upper section of the ‘lower red unit’ of the Tremp Formation (Fig. 2).

Subsequently, Prieto-Márquez et al. [80] described and removed IPS 29920 from the hypodygm of *P. isonensis,* on the basis of: 1) no dentary is known from the holotype and referred materials of *P. isonensis* that would allow comparison with IPS 29920; and 2) IPS 29920 was found 750 m horizontally and over 9 m stratigraphically (actually, over 50 m according to more recent data in Riera et al. [24]; Fig. 2) from the type and referred materials of *P. isonensis*, which prevented any spatial association between the Les Llaus dentary and the Sant Romà d’Abella materials. Prieto-Márquez et al. [80] went on to consider IPS 29920 the holotype of a new genus and species of hadrosaurid, *Koutalisaurus kohlerorum*, based on the extensive lingual projection of the symphyseal process (distance from symphysis to lateral surface of dentary being three times that of the mediolateral width of the bone).

Later, Prieto-Márquez and Wagner [47] reported a comparably extensive lingual projection of the symphyseal process in the dentary of the lambeosaurine *Tsintaosaurus spinorhinus* from the Campanian Jingangkou Formation of eastern China. The occurrence of this symphyseal character in both *Koutalisaurus kohlerorum* and *T. spinorhinus* left *K. kohlerorum* without its only autapomorphy, thus becoming a nomen dubium. Furthermore, the presence in *T. spinorhinus* of a maxilla with elevated jugal joint and dentary with lingually elongate symphyseal process, and the recognition that such elevated type of jugal articular surface of the maxilla was only shared by *P. isonensis*, was used by Prieto-Márquez and Wagner [47] as the basis for referring the Les Llaus dentary back to *P. isonensis*. In their view, the co-ocurrence of such unique maxillary and dentary characters in two temporally (early Campanian *T. spinorhinus*, late Maastrichtian *P. isonensis*) and geographically (eastern Asia *T. spinorhinus*, western Europe *P. isonensis*) distant set of lambeosaurine specimens was more parsimoniously explained as each set representing one species in each continental area.

Recently, however, further preparation of IPS 29920 has revealed that much of the apparent lingual elongation of its symphseal process resulted from the addition of infilling material during the early preparation of the specimen in the late 1990s (Fig. 20E). As currently prepared, after removal of all extraneous material to the bone, the Les Llaus dentary shows a symphyseal process that barely extends lingually twice the width of the alveolar chamber of the bone (Fig. 20D), a condition present in other lambeosaurines like species of *Corythosaurus* and *Amurosaurus riabinini* ([50]: table C.9). A similar situation concerns the apparently symphyseal process of the *Tsintaosaurus spinorhinus* dentaries: much of the extent of this process in the available dentaries of this species appear to have been reconstructed ([44]: text.fig. 12). These revised observations on the lingual extension of the symphyseal processes in IPS 29920 and *T. spinorhinus* (e.g., IVPP V723) invalidates the use of this character as a synapomorphy for these taxa and leaves no basis for the referral of Les Llaus dentary to *P. isonensis*. At this point, all that can be concluded regarding the affinities of IPS 29920 is that it represents an indeterminate lambeosaurine based on the combined presence of strong ventral deflection (i.e., 33° angle between the dorsal alveolar and ventral margins) of the symphyseal process and the fact that this deflection originates near the mid-length of the dental battery [2].

### Euroda Nord Locality

**Figure 21:**
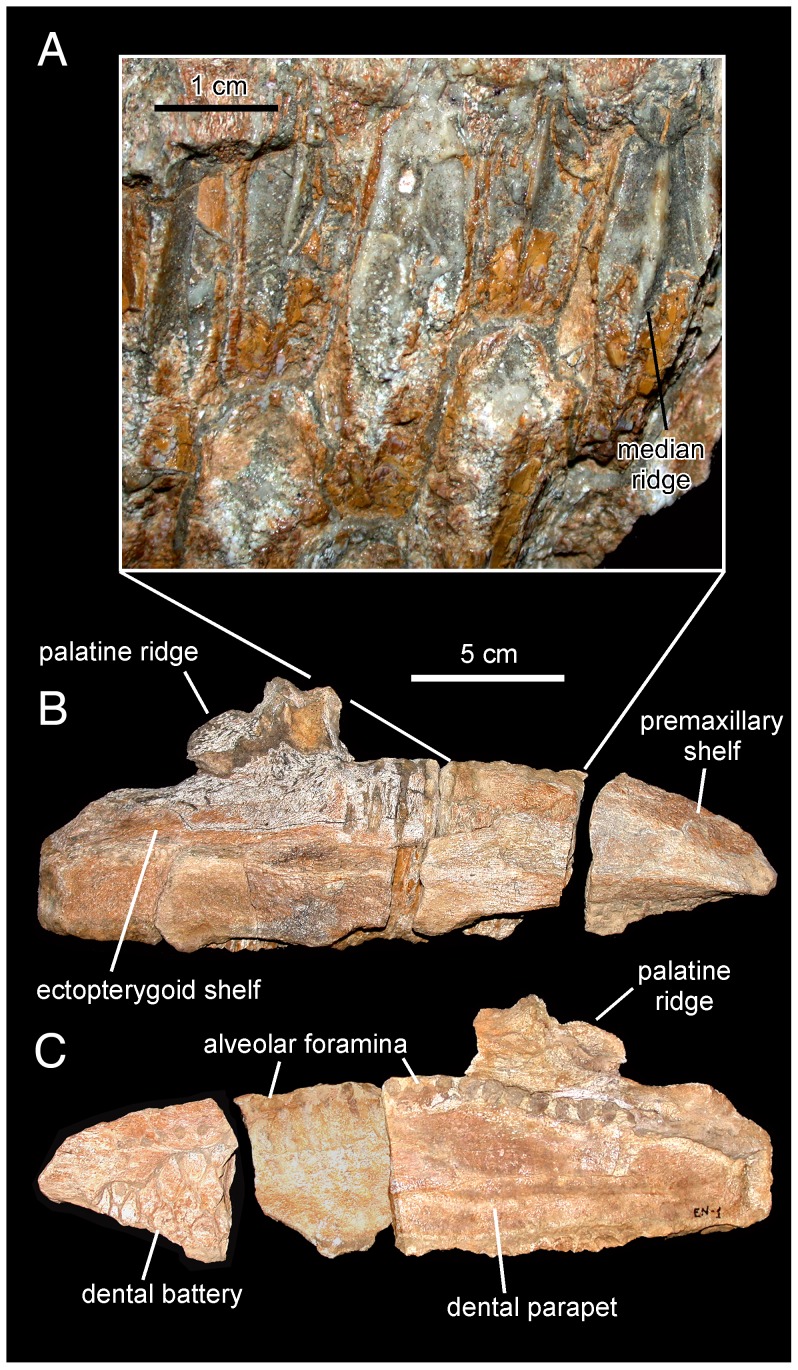
Indeterminate hadrosaurid maxilla from Euroda Nord. A. Maxillary tooth crowns (MCD 5090) in labial view. B. Lateral view of the maxilla. C. Medial view of same.

Recently, Prieto-Márquez et al. [81] and Gaete et al. [82] referred an isolated maxilla (MCD 5090; Fig. 21) collected at the Euroda Nord site to Lambeosaurinae. Euroda Nord is located in the northeastern Tremp Syncline, Lleida province, northeastern Spain (Fig. 1); stratigraphically, it occurs in the upper part of the ‘lower red unit’ of the Tremp Formation [24] (Fig. 2). However, re-examination of MCD 5090, a heavily weathered, partially preserved 240 mm long right maxilla, revealed no lambeosaurine synapomorphies. What remains of this bone is most of the main body and part of the palatine process. Except for the latter partial process, all the structures above the level of the row of special foramina are missing. Additionally, a short section of the rostral third of the maxilla is also missing, as well as the caudal border of the bone and the pterygoid process. This maxilla is mediolaterally narrow, with a flat medial surface. The rostral end is triangular and wedges rostrally to a truncated tip, indicating that the rostral-most end of the bone is not preserved. There is a prominent, nearly horizontal lateral ridge that separates the premaxillary shelf above from the lateral surface of the maxilla below. This shelf lacks the concave relief typical of other hadrosaurids, but this may be an artifact of poor preservation, as the bone surface is so weathered that the spongiosa is visible. Likewise, it is not possible to know whether the maxilla had a rostromedial process, since nothing is preserved dorsal to the level of the special foramina. Given the proportions of the bone, we estimate that the ectopterygoid shelf comprises more than 35% of the total length of the maxilla, as in Hadrosauridae and the non-hadrosaurid hadrosauroids *Telmatosaurus transsylvanicus* and *Lophorhothon atopus* [2]. The shelf is horizontally oriented, a character only found within Hadrosauridae [2]. The preserved part of the palatine process is mediolaterally compressed subrectangular in lateral view, with an irregularly shaped dorsal margin. The maxilla contains a minimum number of 28 tooth positions. Part of the rostral third of the dental battery is exposed in lateral view, showing several tooth crowns. There are at least four teeth arranged vertically per alveolus. Tooth crowns are lanceolate, with a height/width ratio of 2.85 to 3.1. No denticles are observed on their margins and there is a single median carina, which, in some crowns, is sinuous. Mesially in the dental battery, the long axes of the tooth crowns are slightly tilted caudoventrally. Given these anatomical observations, the maxilla from Euroda Nord is referred to an indeterminate hadrosaurid.

### Basturs Poble Locality

**Figure 22:**
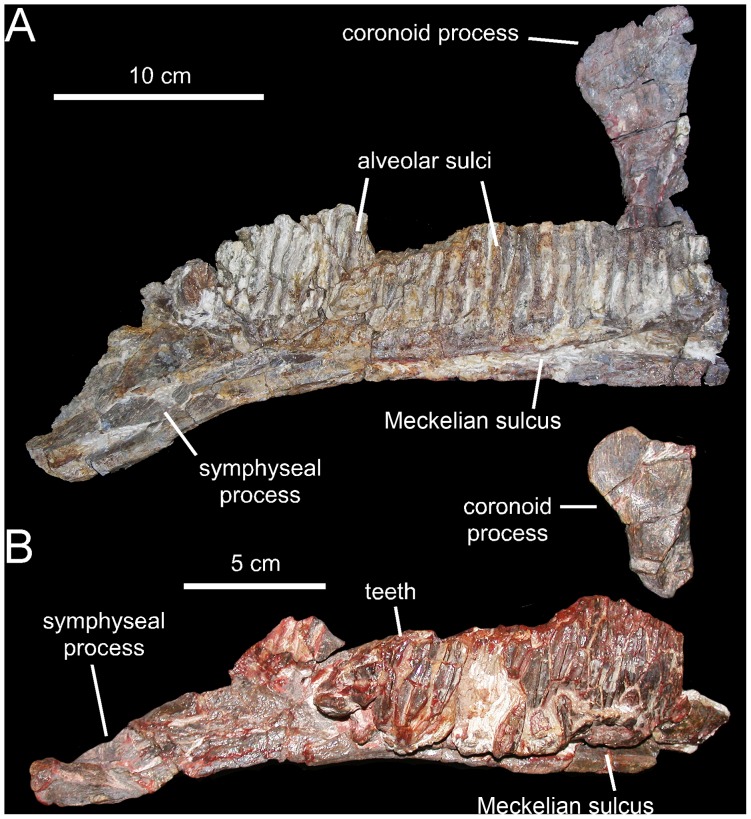
Indeterminate lambeosaurine elements collected at the Basturs Poble bonebed. A. Right dentary (MCD 5007) in medial view. B. Right dentary (MCD 4743) in medial view.

The Basturs Poble fossil site is located in the northeastern Tremp Syncline, Lleida province, northeastern Spain (Fig. 1), consists of a bonebed containing a multi-individual assemblage of disarticulated cranial (Fig. 22), appendicular, and axial hadrosaurid elements, and rarer crocodyliform bones [81], [82]. It occurs in the middle-lower part of the ‘lower red unit’ of the Tremp Formation, 195 m below the lateral equivalents of the Vallcebre limestone [24], [29] (Fig. 2). Prieto-Márquez et al. [81] referred these materials to Lambeosaurinae on the basis of a jugal that is relatively short rostrocaudally and shows truncated expanded rostral process, and a rostrocaudally short maxilla with proportionately wide tall dorsal process. Because the detailed study of the Basturs Poble specimens is currently underway, nothing more can be said at this juncture regarding the anatomy and affinities of these animals.

### Le Bexen Locality

Laurent et al. [15] provisionally referred an edentulous dental battery fragment (MDE-Fo1–11) collected at the upper Maastrichtian Le Bexen locality to *Pararhabdodon* sp. Le Bexen is located in the Les Corbières Orientales, in Aude Department, southern France (Fig. 1). Judging from the published images ([15]: fig. 3 and [17]: pl. 27), MDE-Fo1–11 appears to belong to a dentary, in agreement with Prieto-Márquez et al. [80]. This identification is based on the fact that the morphology of the fossil is consistent with that of the area immediately rostral to and ventral to the coronoid process of the dentary, where the bone widens abruptly mediolaterally. MDE-Fo1–11 may be referred to Hadrosauridae or to a closely related outgroup taxon to Hadrosauridae (*Telmatosaurus transsylvanicus* shows relatively narrow alveolar sulci), based on the presence of the narrow (i.e., interalveolar ridges not wider than 25% of the breadth of individual alveolus) alveolar sulci [2]. Laurent et al. [15] also referred provisionally to *Pararhabdodon* sp. a humerus found in the same locality ([15]: fig. 8). However, because there are no diagnostic characters in the humerus of *Pararhabdodon*, the Le Bexen humerus cannot be referred to this genus.

### Ausseing Locality

**Figure 23:**
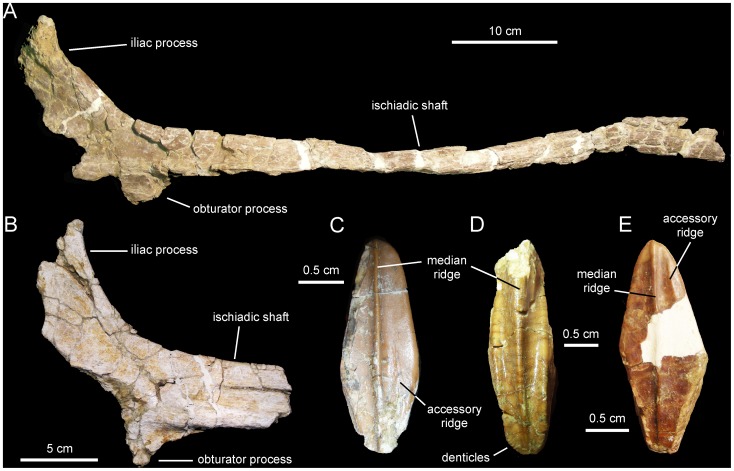
Indeterminate lambeosaurine materials collected at the Ausseing, Cassagnau 1, and Tricouté 1 localities. A. Left ischium (MDE-Aus-185) in lateral view from Ausseing. B. Proximal region of left ischium (MDE-Cas1–200) in lateral view from Cassagnau 1. C. Dentary tooth crown (MDE-Cas1–11) in lingual view from Cassagnau 1. D. Maxillary tooth crown (MDE-Cas1–03) in labial view from Cassagnau 1. E. Dentary tooth crown (MDE-Ma1–01) in lingual view from Tricouté 1.

This site is located near the village of Ausseing, Petites Pyrénées, Haute-Garonne Department, southern France (Fig. 1), and occurs in the middle of the Lestaillats Marls Formation (upper Maastrichtian) (Fig. 2). Laurent [17] referred to Hadrosauridae MDE-Aus-185, a 680 mm long left ischum (Fig. 23A). The bone preserves the iliac process and most of a slender shaft missing its distal end. Notably, the dorsal margin of the iliac process is caudally recurved and shows a ‘thumb-like’ lateral profile. This condition is diagnostic of lambeosaurines [2], [56] and allows referral of MDE-Aus-185 to this clade of hadrosaurids.

### Cassagnau 1 Locality

Cassagnau 1 is found near the town of Marygnac-Lespeyres, in the Petites-Pyrénées, Haute-Garonne Department, southern France (Fig. 1). It occurs in the middle section of the upper Maastrichtian Auzas Marls Formation (Fig. 2). The hadrosaurid bones recovered from this locality include a fragmentary ischium, four tooth crowns, and four manual phalanges. Two features in the ischiadic and dental elements indicate that these elements are referable to Lambeosaurinae (Fig. 23B–D). Thus, the pelvic fragment, MDE-Cas1–200, consists of part of the proximal region of a left ischium preserving the iliac and obturator processes (Fig. 23B). Its caudally recurved dorsal margin, with its ‘thumb-like’ lateral profile, is diagnostic of lambeosaurines [2], [56]. Dentary tooth crowns, such as MDE-Cas1–11, are lanceolate in lingual view and have a height/width ratio of 3.1. Marginal denticles are greatly reduced papillae, slightly more prominent along the apical edge of the teeth. Notably, the teeth show one faint accessory ridge accompanying the prominent median carina (Fig. 23C), a condition typically present in lambeosaurines [1]. Teeth with a thin secondary ridge have also been found in the Tricouté 1 (MCD-Ma1-01; Fig. 23E) and 2 (MCD-Ma2-01; Laurent 2003:plate 30) localities. The only recovered maxillary tooth crown from Cassagnau 1, MDE-Cas1-03 (Fig. 23D), is further elongated and displays a similar denticulation pattern, with larger papillae present along the apical margin of the crown. Only a median prominent ridge is present in this maxillary crown.

### Cassagnau 2 Locality

**Figure 24:**
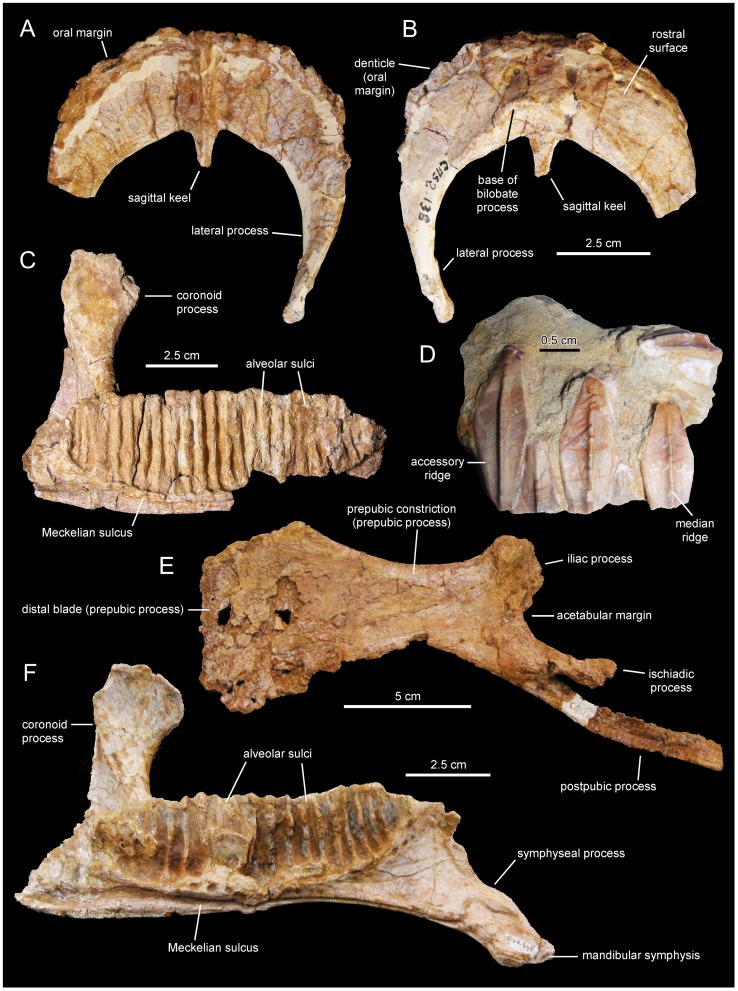
Indeterminate lambeosaurine materials collected at the Cassagnau 2 locality. A. Predentary (MDE-Cas2–138) in dorsal view. B. Ventral view of MDE-Cas2–138. C. Partial left dentary (MDE-Cas2–02) in medial view. D. Dentary tooth crowns (MDE-Cas2–11) in lingual view. E. Left pubis (MDE-Cas2-01) in lateral view. F. Left dentary (MDE-Cas2–248) in medial view.

Laurent [17] referred to Hadrosauridae a few but relatively complete well preserved cranial and postcranial elements (Fig. 24 and Table 5) collected from Cassagnau 2. This locality is found near Cassagnau 1 (Fig. 1). Cassagnau 2 occurs in the middle section of the upper Maastrichtian Auzas Marls Formation (Fig. 2). The bone sample includes a predentary, two left dentaries, various dentary teeth, several cervical vertebrae, a left pubis, a partial right humerus, two femora, a tibia, two fibulae, metatarsals II, III and IV, two pedal phalanges, and a caudal centrum. The postcranial remains were found articulated and belong to a single individual; one dentary (MDE-Cas2–248) was associated to the predentary (MDE-Cas2–138) [16], [17]. All bones belong to relatively small individuals (Table 5). Here we are mainly concerned with those skeletal elements that show diagnostic information relevant to the affinities of the Cassagnau 2 hadrosaurids.

**Table 5:** Selected measurements (in mm) of the Cassagnau 2 lambeosaurines.

Element	Measurement
Predentary (MDE-Cas2-138), length from oral margin to caudal end of right lateral process	55
Predentary (MDE-Cas2-138), maximum width across proximal region of lateral processes	58
Predentary (MDE-Cas2-138), maximum dorsoventral width of rostral surface	22
Dentary (MDE-Cas2-02), length from caudalmost extreme to rostralmost alveolar sulcus	116
Dentary (MDE-Cas2-02), height from ventral margin to apex of coronoid process	83
Dentary (MDE-Cas2-02), height of alveolar sulci at mid-length of dental battery	33
Dentary (MDE-Cas2-248), length from caudal margin of coronoid process to symphysis (measured parallel to dorsal margin of alveolar sulci)	133
Dentary (MDE-Cas2-248), dental battery length	96
Dentary (MDE-Cas2-248), height from ventral margin to apex of coronoid process	72
Dentary (MDE-Cas2-248), height of alveolar sulci at mid-length of dental battery	31
Pubis (MDE-Cas2-01), length from distal end of postpubic process to rostral margin of prepubic process	209
Pubis (MDE-Cas2-01), length from caudal margin of iliac process to rostral margin of prepubic process	136
Pubis (MDE-Cas2-01), depth of distal blade of prepubic process (measured perpendicular to dorsal and ventral margins)	64
Pubis (MDE-Cas2-01), minimum depth of proximal constriction of prepunic process (estimated, incomplete ventral margin)	32

To our knowledge, MDE-Cas2–138 is the only hadrosaurid predentary known so far in the European fossil record. The element is horseshoe-shaped and nearly complete, missing most of the left lateral process (Fig. 24A and B). The rostral surface faces rostroventrally, forming a 40° angle with the long axis of the lateral process. The thin oral margin contained five denticles at each side of the sagittal plane of the element. The best-preserved denticle is subtriangular, with rounded edges. On the lingual surface of the rostral body of the predentary, a prominent sagittal keel projects caudally beyond the ventral margin of that surface. The bilobate process is broken off from the median region of the caudoventral margin of the rostral surface of the bone. The lateral process thins abruptly distally and becomes mediolaterally compressed while gently curving caudomedially. The distal end of the right lateral process is missing, along with most of the narrow lateral shelf.

The two available dentaries (MDE-Cas2-02 and 248; Fig. 24C and F, respectively) lack all teeth. MDE-Cas2–02 preserves 24 alveolai, whereas MDE-Cas2–248 shows 21. Although eroded in the more complete MDE-Cas2–248 dentary, enough is preserved of the proximal edentulous margin to show that it is shorter than 20% of the length of the dental battery, as in *Arenysaurus ardevoli* and *Blasisaurus canudoi* (see above). However, in contrast to the gently deflected symphyseal processes of the Blasi taxa, that of MDE-Cas2–248 (excluding the abrupt bending of symphysis proper) is more steeply oriented rostroventrally to form a 26° angle with the ventral margin of the dentary ramus. The coronoid process is twice as tall as the dentary ramus and slightly inclined rostrally, its caudal border forming a 78° angle with the dorsal alveolar margin (measured in lateral view).

Dentary tooth crowns (MDE-Cas2–11; Fig. 24D) are lanceolate in lingual view, with a height/width ratio of 2.84. The enameled lingual side displays a large median ridge; in the most complete teeth, there is also a faint shorter accessory ridge (Fig. 23D). Marginal denticles are extremely shallow papillae that are slightly more conspicuous near the apical end of the crown.

The pubis (MDE-Cas2-01) is practically complete (Fig. 24E). The acetabular region is composed of a tetrahedral iliac process dorsally and a finger-shaped ischiadic process ventrally. The subtriangular acetabular surface of the iliac process faces caudolaterally. The distal articular end of the ischiadic process is expanded substantially. Beneath the latter, a long mediolaterally compressed, tape-like postpubic process projects caudoventrally, being continuous with the ventral margin of the pubis. The prepubic process shows a narrow proximal constriction and a greatly expanded distal blade. The dorsal and ventral margins of the blade are parallel and rostroventrally oriented. The distal prepubic blade is relatively short craniocaudally, so that the concave dorsal margin of the proximal constriction is about twice as long as the blade. The distal blade is slightly deeper that the width of the acetabular margin (from the dorsal border of the iliac process to the distal tip of the ischiadic process) and twice as deep as the minimum breadth of the proximal constriction.

The above description of the morphology of the prepubic process conforms to that present in lambeosaurine hadrosaurids [2], [50], [56]. Specifically, the proportionately short subsquared or subtrapezoidal outline, with more prominent rostroventral corner, of the distal prepubic blade of the prepubic process is only found among lambeosaurines, such as *Parasaurolophus cyrtocristatus* (e.g., FMNH P27393), *Corythosaurus casuarius* (e.g., AMNH 5240), and *Magnapaulia laticaudus* (e.g., LACM 20874). Although incomplete in *Arenysaurus ardevoli* (MPZ 2007/707 [66]: p. 212), the prepubic process of the Blasi lambeosaurine shares with that of Cassagnau 2 a proportionately short but greatly expanded distal prepubic blade. Therefore, the Cassagnau 2 material is referred here to Lambeosaurinae on the basis of the prepubic morphology and the presence of accessory ridges in at least some dentary teeth. The reduced proximal edentulous margin of the dentaries might be an indication of the juvenile nature of the specimens when combined with their relatively small size (Table 5), as the relative length of that margin increases during ontogeny among hadrosaurids; the majority of lambeosaurine taxa include adult individuals with proximal edentulous margins longer than 20% and nearly up to 40% of the length of the dental battery [2], [50].

### Phylogenetic Relationships of the European Lambeosaurinae

**Figure 25:**
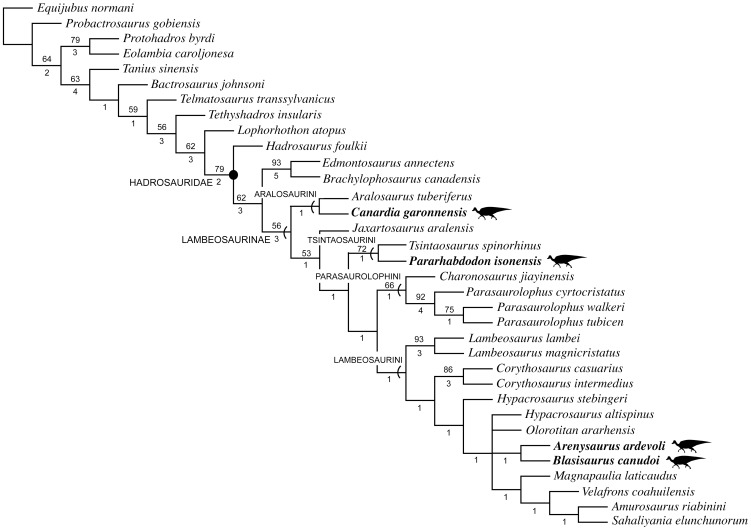
Phylogenetic relationships of lambeosaurine taxa from the European Archipelago. Consensus tree of the five most parsimonious trees resulting from maximum parsimony analysis. The numbers above the branches represent bootstrap frequencies, whereas those below are decay indices (Bremer support).

A maximum parsimony analysis was conducted for inferring the phylogenetic position of the four lambeosaurine species known from the European Archipelago. The analysis returned five equally most parsimonious trees of 588 steps each (CI = 0.61, RI = 0.75), a score found in 9,960 of the 10,000 replicates (Fig. 25). European taxa fell in three different clades within Lambeosaurinae. Thus, *Canardia garonnensis* was recovered as the sister taxon to *Aralosaurus tuberiferus*, together integrating the tribe Aralosaurini. Both taxa are unambiguously united by having maxillae with an angle between the dorsal margin of the rostroventral process and the rostral segment of the tooth row greater than 25° and up to 35° (convergent in *Parasaurolophus walkeri* and *P. tubicen*); rostrocaudally broad laterally exposed surface of the rostrodorsal region of the maxilla, adjacent and rostral to the jugal articular surface; and rostrodorsal margin bearing a prominent subrectangular flange that rises vertically above the rostroventral process.

This study recovered Prieto-Márquez and Wagner’s [47] sister taxon relationship integrated by *Pararhabdodon isonensis* and *Tsintaosaurus spinorhinus*. This relatively basal clade of lambeosaurines is supported by two unambiguous synapomorphies in the maxilla: elevation of the articular facet for the jugal, such that the ventral-most extent and ventral jugal tubercle lie above the level of the ectopterygoid ridge; and presence of an acute embayment extending ventral to the ventral jugal tubercle between the jugal facet and the ectopterygoid shelf.

The Blasi species, *Arenysaurus ardevoli* and *Blasisaurus canudoi*, appear deeply nested within Lambeosaurinae (Fig. 25). These species form a sister taxon relationship unambiguously supported by three synapomorphies of the dentary: relatively short proximal edentulous slope, such that the ratio between the length of this slope and the distance between the rostralmost tooth position and the caudal margin of the coronoid process is less than 0.2 (convergent in *Velafrons coahuilensis*); angle of deflection of the ventral margin of the dentary up to 20°; and caudal end of the dental battery being flush with the caudal margin of the coronoid process. Inclusion of the two Blasi species within the lambeosaurin clade consisting of the last common ancestor of *Hypacrosaurus altispinus*, *Amurosaurus riabinini*, and all its descendants, is supported by one ambiguous synapomorphy: absent or very poorly developed ventral transverse caudal ridge between the basipterygoid processes of the basisphenoid (scoring of this character unknown in *Olorotitan ararhensis*). At a more inclusive level, the *Arenysaurus*-*Blasisaurus* clade is unambiguously positioned within Lambeosaurini on the basis of a nasal articulation surface of the frontal shaped into a rostroventrally-sloping platform; bifurcation of the rostromedial margin of the frontals at the sagittal plane of the skull roof; and moderate length of the precotyloid process of the squamosal, being between 0.95 and 1.25 times the width of the quadrate cotylus. These synapomorphies cannot be observed in the available materials of *B. canudoi*; however, because of the sister taxon relationship of the latter with *A. ardevoli*, those characters were optimized for the entire Blasi clade.

The phylogenetic position of the Blasi lambeosaurines recovered in the present analysis differs from that of previous studies [11], [12], [66]. In particular, *Arenysaurus ardevoli* [11] and the *A. ardevoli*-*Blasisaurus canudoi* clade [12] were positioned outside the clade consisting of parasaurolophins and lambeosaurins. More recently, however, Cruzado-Caballero [66] recovered the Blasi taxa as members of Parasaurolophini (but see below). The topological differences between the phylogenies proposed by those authors and the one herein presented stem from the use of character matrices containing substantially different information. Specifically, the parsimony analyses of Pereda-Suberbiola et al. [11] used only 57 characters (208 less than in our analysis); notably, the character matrix used by these authors lacked postcranial characters, whereas that used in our analysis includes 86 characters representing the entire hadrosaurid postcranial skeleton. Cruzado-Caballero et al. [12] included more anatomical information in their 138-character matrix, yet it represented only nearly half of the total number of characters used here. Cruzado-Caballero [66] performed two analyses, one using the data matrix of Reisz and Evans [64] and another using the data set of Sues and Averianov [83]. Both matrices consist also of only a fraction of the morphological characters used in the present analysis and, therefore, it is hardly surprising that the analyses based on those matrices resulted in different topologies from that shown in Fig. 25.

In the analysis of Cruzado-Caballero ([66]: figs. 5.19 and 5.20), *A. ardevoli* and *B. canudoi* formed a clade with *Parasaurolophus* spp. Inclusion of the Blasi species within Parasaurolophini was solely supported by equilateral cranial ascending process of the astragalus and expanded distal end of the fibula. However, the fibula and astragalus are not preserved in *A. ardevoli* and *B. canudoi*. Characters supporting the close relationship of the Blasi taxa with *Parasaurolophus* spp. included: broad distal condyles of the humerus; relatively deep short prepubic constriction that becomes expanded at the base of the proximal end of the prepubic process; and postorbital with short deep squamosal ramus, indicative of a narrow dorsal margin of the infratemporal fenestra. Notwithstanding the heavily eroded distal humeral region of *A. ardevoli* ([11]: fig. 5), there is much intrataxonomic variation in the breadth of the distal condyles of the hadrosaurid humerus [50], so that we do not consider this character as phylogenetically informative. A relatively deep short proximal constriction of the prepubic process is certainly found in *P. walkeri* (e.g., ROM 768) and *P. cyrtocristatus* (e.g., FMNH P27393) (no pubis is known for *P. tubicen*) but also in *Hypacrosaurus altispinus* (e.g., CMN 8501), *H. stebingeri* (e.g., MOR 549), and *Magnapaulia laticaudus* [46]. Ventral expansion of the prepubic process occurs in all lambeosaurines [2]. Furthermore, the ventral region of the prepubic process of *A. ardevoli* is too incompletely preserved to ascertain the extent and geometry of the ventral expansion of that process. We agree, however, in that *A. ardevoli* possesses a deep rostrocaudally abbreviated squamosal ramus of the postorbital, with concomitant shortening of the dorsal margin of the infratemporal fenestra, which acquires an arcuate lateral contour. This condition is certainly shared by *P. walkeri* and *P. tubicen*, but not by *P. cyrtocristatus*. Indeed, *P. cyrtocristatus* shows a relatively longer squamosal process and a straight, subrectangular dorsal margin of the infratemporal fenestra, more similar to that seen in lambeosaurin lambeosaurines like *Corythosaurus* spp., *Lambeosaurus* spp., or *Hypacrosaurus* spp. It is worth noting that the basal lambeosaurine *Jaxartosaurus aralensis* also possesses a deep short squamosal process of the postorbital [84]. In our analysis, this condition of the postorbital represents an unambiguous synapomorphy of *P. tubicen* and *P. walkeri*, and appears as independently derived in *J. aralensis* and *A. ardevoli*. Therefore, given the available anatomical data for the Blasi lambeosaurines, our study does not support the presence of parasaurolophins in the Ibero-Armorican island of the European Archipelago.

### Historical Biogeography of the European Lambeosaurinae

A global sea level rise that began during Albian times and culminated during the late Cenomanian-early Turonian [85] created an archipelago of islands between the Afroarabian plate and the emergent part of the Fennosarmatian craton (the northern European land of Dalla Vecchia [14]). During the Late Cretaceous, the convergent movements of the Afroarabian and Eurasian plates, caused by the opening of the southern Atlantic, and the consequent collision of microplates existing in between, caused local tectonic uplift and the formation of emergent areas [86]–[90].

The European Archipelago [7], [11], [91] consisted of the Anglo, Ibero-Armorican, Renish-Bohemian, Adriatic, Australpine, and Transylvanian ( = Tisia–Dacia Island or Haţeg) islands, among others. Different authors have variably reconstructed the number and area of those islands. For example, Camoin et al. [92] depicted a unique large Ibero-Armorican-Renish-Bohemian island, while Philip et al. [93] set the Ibero-Armorican apart from the Renish-Bohemian island. According to Le Loeuff [94], the area of Ibero-Armorican island, which harbored the lambeosaurine taxa described and discussed in this study, ranged between 600,000 and 1,500,000 km^2^, thus being at least as large as Madagascar (587,000 km^2^). Likewise, the identity of the Transylvanian island (i.e., the paleogeographical setting of the Haţeg Basin and its Late Cretaceous vertebrate faunas) varies according to the reconstructions proposed by various authors [7], [91], [95]–[97].

In this paleogeographical context, the late Maastrichtian European hadrosaurian fauna is characterized by lambeosaurines (*Arenysaurus ardevoli*, *Blasisaurus canudoi*, *Pararhabdodon isonensis*, and *Canardia garonnensis* from the Ibero-Armorican island; this paper) and non-hadrosaurid hadrosauroids (indeterminate forms represented by the Fontllonga [53], [55] and La Solana dentaries [54], [55]). Yet, to this date, no pre-Maastrichtian remains unambiguously referable to Lambeosaurinae or Hadrosauridae have been recorded in Europe [33].

In North America, lambeosaurines are common and diverse in upper Campanian strata (*Lambeosaurus* spp., *Corythosaurus* spp., *Parasaurolophus* spp., *Hypacrosaurus stebingeri*, *Velafrons coahuilensis*, *Magnapaulia laticaudus* [46], [65], [98]. In the Maastrichtian, North American lambeosaurines are solely represented by *Hypacrosaurus altispinus*, which might range from the lower to the lower upper part of the stage [49], [69]. Indeed, the most common dinosaur in the rich paleontological record of the continental uppermost Maastrichtian of North America (Scollard, Frenchman, Lance, and Hell Creek formations) is the saurolophine *Edmontosaurus annectens* [99]. In contrast, lambeosaurines are diverse and relatively common in the upper Maastrichtian of eastern Asia (*Amurosaurus riabinini*, *Charonosaurus jiayinensis*, *Olorotitan arharensis*, and *Sahaliyania elunchunorum* [6], [57], [60], [62]. In addition, non-hadrosaurid hadrosauroids like *Bactrosaurus johnsoni* [51] and *Gilmoreosaurus mongoliensis* [100] lived in Central Asia during the late Campanian-Maastrichtian, possibly in latest Campanian-early Maastrichtian times ([101] but see Averianov and Sues [102] for a different dating), being absent in the late Maastrichtian. Thus, the taxonomic composition of the European hadrosaurian fauna is more similar to the coeval fauna from Asia than that of North America [33].

**Figure 26:**
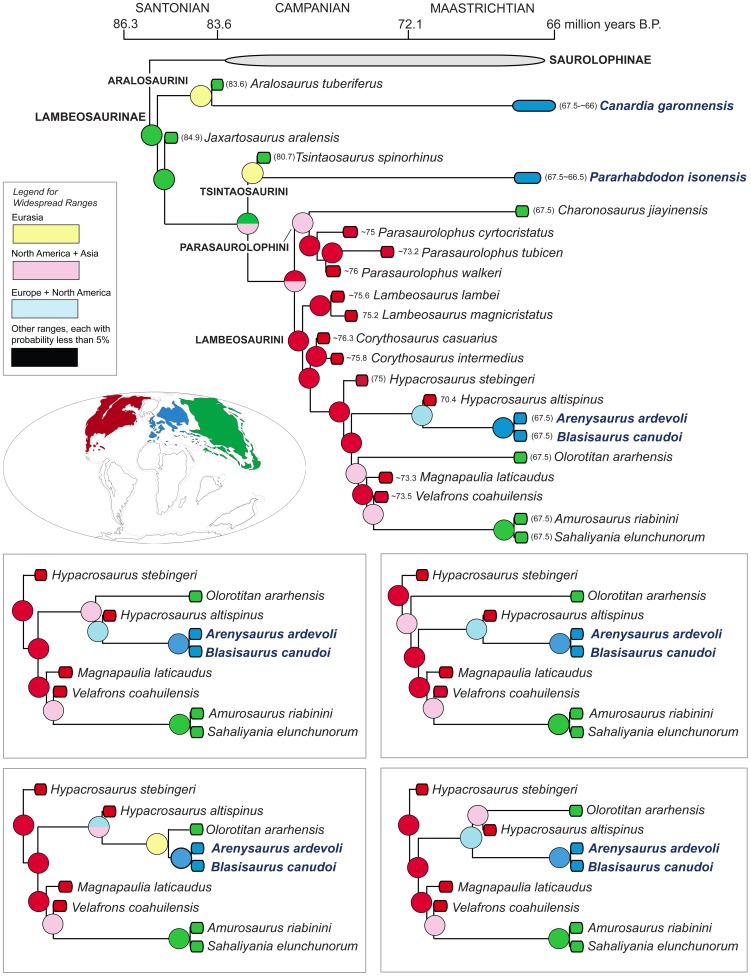
Time-calibrated phylogram of Lambeosaurinae based on the phylogenetic hypothesis shown in Fig. **25.** Each of the five most parsimonious trees resulting from parsimony analysis is shown. However, because those trees differ only in the relationships of the *Hypacrosaurus-Amurosaurus* clade, we only show the complete topology of one of the threes and include below only the topologies of that subclade for the additional four most parsimonious trees. The circles at each node represent the relative probabilities for the ancestral areas inferred using Statistical Dispersal-Vicariance Analysis (S-DIVA [104]), implemented in RASP 2.0b [127]. The numbers to the left of the taxon names are datings in millions of years. When absolute dating estimates are not available but only subages (e.g., late Maastrichtian), the absolute dating of the taxon is approximated as the mid-point of the available range (numbers between brackets). The literature sources for each taxon’s geochronological range are as follows: *Amurosaurus riabinini* (late Maastrichtian range for the Udurchukan Formation [64]); *Aralosaurus tuberiferus* (late Santonian-early Campanian range for the Bostobe Formation [43]); *Arenysaurus ardevoli* and *Blasisaurus canudoi* (late Maastrichtian; Fig. 2); *Charonosaurus jiayinensis* (late Maastrichtian range for the Yuliangze Formation [60]); *Canardia garonnensis* (late Maastrichtian up to near the K-Pg boundary; Fig. 2); *Corythosaurus* spp., *Lambeosaurus* spp., and *Parasaurolophus walkeri* [98]; *Hypacrosaurus altispinus* [49]; *H. stebingeri* (‘middle’ to late Campanian range for the upper section of the Two Medicine Formation [1]); *Magnapaulia laticaudus* [131]; *Jaxartosaurus aralensis* (Santonian range for the Syuksyuk Formation [132]); *Olorotitan arharensis* (late Maastrichtian range for the Udurchukan Formation [6]); *Pararhabdodon isonensis* (late to latest Maastrichtian; Fig. 2); *Parasaurolophus tubicen* and *P. cyrtocristatus* [133]; *Sahaliyania elunchunorum* (late Maastrichtian range for the Yuliangze Formation according to Godefroit et al. 2008); *Tsintaosaurus spinorhinus* (early Campanian range for the Jingangkou Formaton [134]); and *Velafrons coahuilensis* [135]. Datings for age boundaries are from Walker et al. [136].

Our Statistical Dispersal-Vicariance Analysis (or S-DIVA [103], [104]; Fig. 26) inferred a Eurasian ancestral range for the most recent common ancestors of aralosaurins and tsintaosaurins. This would imply that the divergences of *Canardia garonnensis* from *Aralosaurus tuberiferus* and that of *Pararhabdodon isonensis* from *Tsintaosaurus spinorhinus* represent vicariant events occurring no later than the early Campanian (Fig. 26). Vicariance was previously suggested by Casanovas et al [77] to explain the occurrence of *P. isonensis*. Likewise, a widespread ancestor was also inferred for the most exclusive clade containing *Hypacrosaurus altispinus* and the Blasi lambeosaurins, *Arenysaurus ardevoli* and *Blasisaurus canudoi*. However, the labile position of *Olorotitan ararhensis* within Lambeosaurini leads to two different ancestral range reconstructions for that clade. Specifically, when *O. ararhensis* appears as sister taxon to the Blasi taxa, their most recent common ancestor was reconstructed as living in Eurasia; in all other cases, that ancestor was inferred to have been widespread in North America and the European Archipelago. Yet, in all cases, the presence of the Blasi lambeosaurins in the Ibero-Armorican island would represent another case of vicariance that took place during the Maastrichtian (Fig. 26).

**Figure 27:**
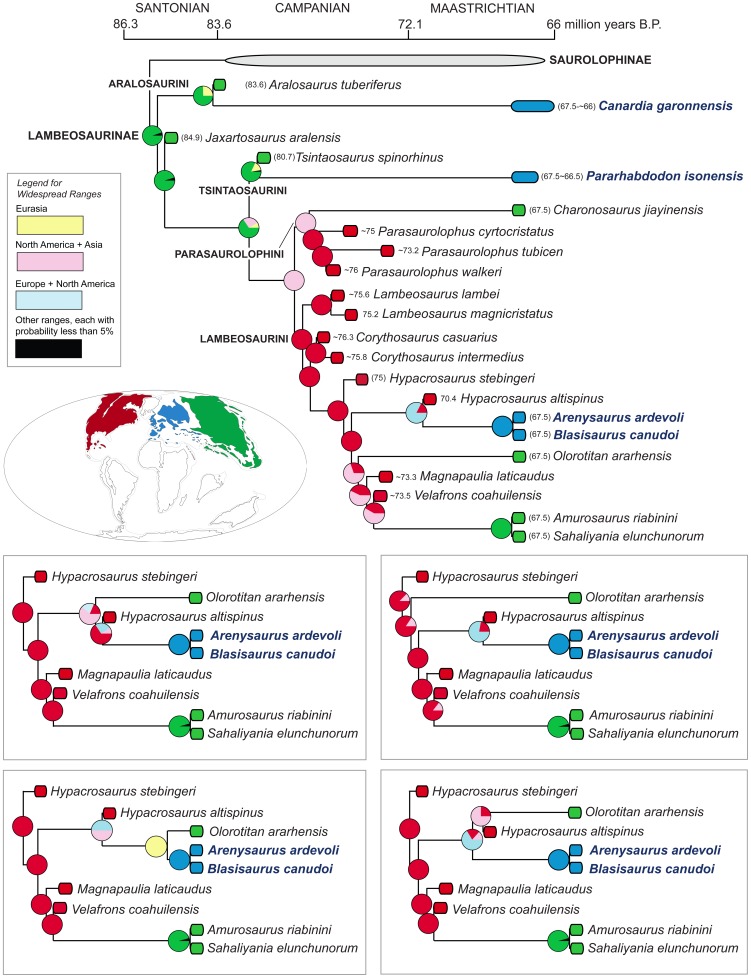
Time-calibrated phylogram of Lambeosaurinae based on the phylogenetic hypothesis shown in Fig. 25. The circles at each node represent the relative probabilities for the ancestral areas inferred using the Bayesian Binary MCMC method (BBM) of Yu et al. [105], implemented in RASP 2.0b [127].

**Figure 28:**
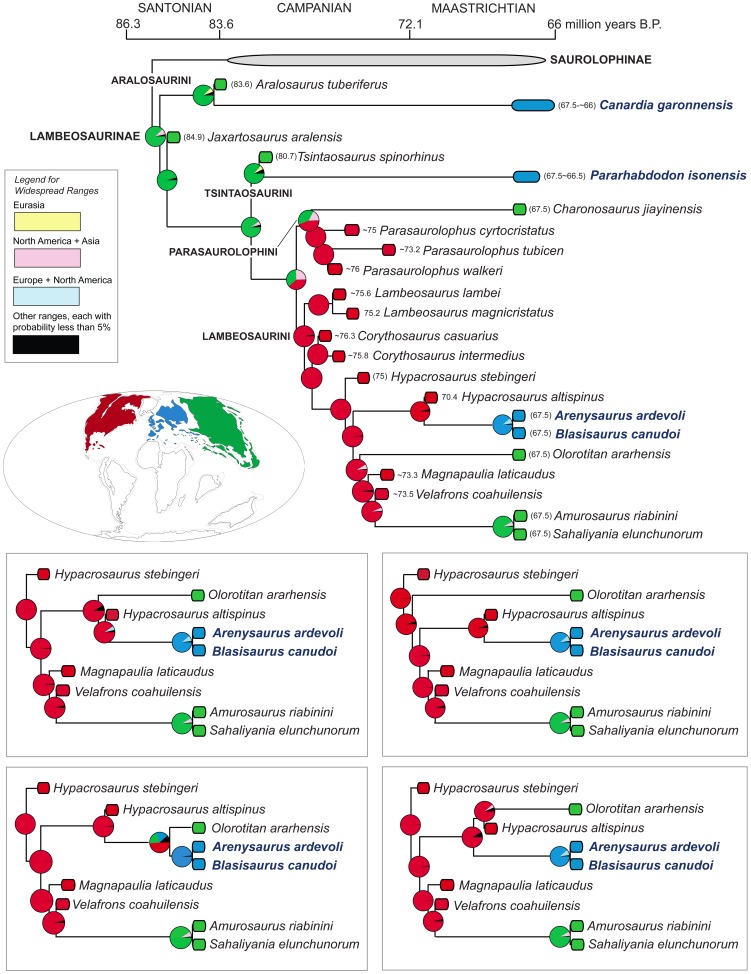
Time-calibrated phylogram of Lambeosaurinae based on the phylogenetic hypothesis shown in Fig. 25. The circles at each node represent the relative probabilities for the ancestral areas inferred via Dispersal Cladogenesis Extinction analysis (DEC [129]), implemented in RASP 2.0b [127].

The above scenarios stand in contrast with the results from both the Bayesian Binary MCMC (or BBM [105]; Fig. 27) and Dispersal Extinction Cladogenesis (or DEC [106]; Fig. 28) analyses. Both techniques inferred Asia as the most likely ancestral range for aralosaurins and tsintaosaurins. According to this reconstruction, the occurrences of *Canardia garonnensis* and *Pararhabdodon isonensis* would be the result of dispersal events from Asia to the western European Archipelago. More uncertain is the biogeographical history of the most exclusive clade of lambeosaurins including the two Blasi species and their common sister taxon, complicated, again, by the labile phylogenetic position of *Olorotitan ararhensis*. On one hand, the results of the BBM analysis indicate North America as the most probable ancestral area of that clade in all five most parsimonious trees depicting lambeosaurine relationships (Fig. 27). Following this reconstruction, *Arenysaurus ardevoli* and *Blasisaurus canudoi* would have reached the Ibero-Armorican island via dispersal events from North America sometime during the Maastrichtian (Fig. 27). On the other hand, the results from the DEC analysis vary substantially depending on the position of *O. ararhensis*, showing either Eurasia, North America plus the European Archipelago, or solely North America, as the most likely ancestral ranges for that exclusive clade containing the Blasi lambeosaurins (Fig. 28). These inferences allow for several biogeographical scenarios for the Blasi taxa occurring sometime during the Maastrichtian, from vicariance leading to the splitting of Asian or North American from European ranges to a dispersal event from North America to the European Archipelago (Fig. 28).

The vicariant scenarios suggested by the S-DIVA results are at odds with the current fossil record of European lambeosaurines. As indicated above, no lambeosaurine fossils have been positively identified in pre-upper Maastrichtian strata, despite remains of hadrosauroids being reported from upper Campanian-Maastrichtian deposits in Belgium, the Netherlands, Germany, Slovenia, Italy, Bulgaria, Romania, and Ukraine [7], [8], [33], [107]. Lambeosaurines are also absent in the upper Campanian-lower Maastrichtian terrestrial vertebrate localities of the Iberian Peninsula like Beira Litoral in Portugal [108] and Laño [109], [110], Korres y Apellaniz [111], Quintanilla del Coco [112], Armuña [113], Cubilla [114], Lo Hueco [115], Sacedón [116], and Chera [117] in Spain. They are also unrecorded in the upper Campanian-lower Maastrichtian of southern France [17], [29]. The only putative evidence of late Campanian hadrosaurids in Europe is a single tooth from the Laño locality [109]; Santonian-upper Campanian European vertebrate localities are devoid of hadrosaurid remains, although these sites preserve rhabdodontid iguanodontians [8], [72]. Therefore, the ancestral ranges inferred via the BBM and DEC analyses and corresponding dispersal scenarios are more consistent with the known record of lambeosaurines in Europe. Aralosaurins and tsintaosaurins appear to have reached the Ibero-Armorican island at the end of the early Maastrichtian or during the late Maastrichtian. Biogeographical scenarios involving dispersal events for lambeosaurines from Asia to the European Archipelago have been previously proposed by various authors [11], [12], [47], [66], although their middle to late Campanian timing of those dispersals is earlier than our estimate based on the earliest fossil occurrence of these hadrosaurids in European strata. The Ibero-Armorican Island constituted a refugium for aralosaurin and tsintaosaurin lambeosaurines during the late Maastrichtian.

## Methods

Permission was granted to the authors for accessing the collections at the MCD (Isona, Spain), MDE (Espéraza, France), MPZ (Zaragoza, Spain), and IPS (Sabadell, Spain) in order to examine the lambeosaurine materials housed at these institutions. None of the specimens were purchased, donated, or loaned; the specimens were observed in situ at those institutions.

### Phylogenetic Inference

A maximum parsimony analysis was conducted for inferring the phylogenetic position within Lambeosaurinae of all the European taxa known so far referable to this clade of hadrosaurids. The analysis included 34 operational taxonomic units. Of these, nine species are outgroup taxa to Hadrosauridae. The remaining taxa consisted of three non-lambeosaurine hadrosaurids (with *Edmontosaurus annectens* and *Brachylophosaurus canadensis* as representatives of Saurolophinae) and 22 lambeosaurine species. The data set consisted 265 equally weighted morphological characters (179 cranial and 86 postcranial; see Supporting Information S1 and S2). All characters, except 1, 4, 16, and 19, were left unordered, following the recommendation of Prieto-Márquez [2]. The search for the optimal tree(s) was conducted in PAUP version 4.0b10 [118]. A heuristic search of 10,000 replicates using random additional sequences was performed, followed by branch swapping by tree-bisection-reconnection (TBR [119], holding ten trees per replicate. Bremer support [120] was assessed by computing decay indices [121] using MacClade version 4.0 [122] and PAUP [118]. Bootstrap proportions [123] were also calculated using PAUP, setting the analysis to 5,000 replicates using heuristic searches, in which each search was conducted using random additional sequences with branch-swapping by subtree pruning and regrafting (SPR) and 25 replicates.

### Reconstruction of Ancestral Ranges

The biogeographical analyses rested upon consideration of the phylogenetic hypothesis of lambeosaurine interrelationships presented in this study. Three general areas, where currently known lambeosaurine species have been recorded, were considered: Europe (or European Archipelago in Late Cretaceous times), North America, and Asia. Ancestral ranges for the clades recovered in the lambeosaurine phylogenies presented here were quantitatively inferred via event-based methods of cladistic biogeography. These techniques integrate phylogenetic information with optimality criteria [124]. Because the techniques implemented here for ancestral range reconstruction require a fully bifurcating phylogeny, we applied them to each of the five most parsimoniuous trees resulting from the parsimony analysis. This option was preferred instead of other alternatives for obtaining an entirely resolved phylogeny, such as pruning taxa from the consensus tree or using a maximum agreement subtree. This is because the latter options allow for obtaining a fully bifurcating phylogemy to the expense of deleting taxa and its associated recorded geographical areas; the omission of taxa may seriously affect the biogeographical results by creating spurious ancestral range reconstructions. Here, we compared the results of three event-based techniques.

The fist method used is Statistical Dispersal-Vicariance Analysis (S-DIVA). Dispersal-Vicariance Analysis was originally developed by Ronquist [103]. This technique uses a model in which vicariance, sympatric speciation, dispersal, and extinction events are given different costs that are inversely related to the likelihood of occurrence of these events. Specifically, vicariance (speciation due to emergence of a dispersal barrier) and duplication (speciation within the same area) have a cost of zero, whereas dispersal and extinction events have a cost of one per each area unit added or deleted, respectively, from the distribution [103]. DIVA uses parsimony as optimality criterion and searches for the reconstruction that minimizes the number of dispersal-extinction events (or cost) required to explain the geographical distribution of terminal taxa [103]. A modification of DIVA, S-DIVA integrates the methods of Nylander et al. [125] and Harris and Xiang [126] in order to compute statistical support for the reconstruction of each ancestral range [104]. In S-DIVA, the frequencies of each ancestral range for a given node of the phylogeny are averaged over all trees, so that each alternative ancestral range at a node is weighted by the frequency of occurrence of the node [104]. The method was implemented in the program RASP 2.0b [105], [127].

A second technique of ancestral range reconstruction implemented in this study is Bayesian Binary MCMC analysis (BBM [105]; see Supporting Information S3). This technique was also implemented in RASP 2.0b [105], [127]. BBM infers ancestral ranges statistically using the full hierarchical Bayesian approach of Ronquist [128], in our case using a Jukes Cantor model with fixed state frequencies and equal among-site rate variation. We set the analysis to 1,000,000 cycles for the Markov Chain Monte Carlo (MCMC) computation, running 100 chains for each MCMC cycle, and setting the number of sample frequencies to 100; the first 100 samples were discarded.

Finally, we implemented the Dispersal Extinction Cladogenesis (DEC) method [106] (see Supporting Information S4). DEC is based on a likelihood framework that models the dispersal and local extinction of lineages as stochastic events through time [106], [129]. Monte Carlo techniques allow estimation of the probabilities of ancestral ranges for each branch of the tree. Thus, DEC evaluates the likelihood of the observed occurrence of taxa given information of their phylogenetic relationships and the paleogeographic and geologic history of the areas. This approach considers all possible scenarios of subdivision and inheritance of ancestral areas, including sister lineages inheriting nonidentical areas [106], [129]. Likelihood values are provided for each internal node of the tree, as well as a global likelihood for the entire phylogeny. DEC was implemented in the program RASP 2.0b [127]. No restrictions were placed on the various possible dispersals among areas and various ranges achievable by the taxa under study.

Implementation of DEC required branch length calibration of the various lineages in the lambeosaurine phylogenies. We applied the strict calibration technique, which uses point estimates of the age of each taxon and the topology of the tree to temporally calibrate the phylogeny, including minimum length ghost lineages. Resulting zero length branches (i.e., those with a duration that cannot be calculated by direct ghost lineage extension) were given a length of 0.1, a value that is much smaller than the minimum possible difference between two taxa of different ages [130], chosen because it has little to no impact in the calibration relative to other branches.

### Nomenclatural Acts

The electronic edition of this article conforms to the requirements of the amended International Code of Zoological Nomenclature, and hence the new names contained herein are available under that Code from the electronic edition of this article. This published work and the nomenclatural acts it contains have been registered in ZooBank, the online registration system for the ICZN. The ZooBank LSIDs (Life Science Identifiers) can be resolved and the associated information viewed through any standard web browser by appending the LSID to the prefix “http://zoobank.org/”. The LSID for this publication is: urn:lsid:zoobank.org:pub:5EEBBFFF-2B86-401B-B308-FC8F5604D74C. The electronic edition of this work was published in a journal with an ISSN, and has been archived and is available from the following digital repositories: PubMed Central and LOCKSS.

## Supporting Information

Information S1 **Characters used in the maximum parsimony analysis for inferring the phylogenetic relationships of the lambeosaurine taxa and specimens from the European archipelago.** Internet links is some of the characters correspond to the illustration and documentation of character states in Morphbank, an online repository for biological images.
(DOC)

Information S2 **Character state codings of 265 morphological characters for the 34 hadrosauroid taxa used to infer the phylogenetic interrelationships of lambeosaurine hadrosaurids.**
(NEX)

Information S3 **Results of the Bayesian-Binary MCMC analysis** **[105]** **performed on each of the five most parsimonious trees resulting from maximum parsimony analysis of lambeosaurine relationships.** Numbers represent probability proportions of inferred ancestral areas. Node numbers correspond to those in the phylograms included below.
(PDF)

Information S4 **Results of the Dispersal-Extinction-Cladogenesis analysis** **[106]**, **[129]** **performed on each of the five most parsimonious trees resulting from maximum parsimony analysis of lambeosaurine relationships.** Numbers represent probability proportions of inferred ancestral areas. Node numbers correspond to those in the phylograms included below.
(PDF)
