# Supplementary material for: Diversity, Relationships, and Biogeography of the Lambeosaurine Dinosaurs from the European Archipelago, with Description of the New Aralosaurin Canardia garonnensis
Source: PLoS One. 2013 Jul 26;8(7):e69835. doi: 10.1371/journal.pone.0069835 (PMC3724916; doi:10.1371/journal.pone.0069835)
Supplement: Information S1 — Characters used in the maximum parsimony analysis for inferring the phylogenetic relationships of the lambeosaurine taxa and specimens from the European archipelago. Internet links is some of the characters correspond to the illustration and documentation of character states in Morphbank, an online repository for biological images. (DOC) [file pone.0069835.s001.doc]

Supporting Information S1

Characters used in the maximum parsimony analysis for inferring the phylogenetic relationships of the lambeosaurine taxa and specimens from the European archipelago. Internet links in some of the characters correspond to the illustration and documentation of character states in Morphbank, an online repository for biological images.

Dental Characters

1. Maximum number of tooth positions in the dentary dental battery (DTTH1, <http://www.morphbank.net/Show/?id=460667>; modified from Horner et al., 2004, character 1): 30 or less (sample mean of 22 alveolar positions) (0); 31 to 42 (sample mean of 37 alveolar positions) (1); more than 42 (sample mean of 49 alveolar positions) (2).
2. Minimum number of teeth per alveoli arranged dorsoventrally at mid length of the dental battery (DTTH3, <http://www.morphbank.net/Show/?id=461202>; modified from Horner et al., 2004, character 2): two (0); three (1); four; (2) five or more (3).
3. Maximum number of functional teeth exposed on the dentary occlusal plane (DTTH4, <http://www.morphbank.net/Show/?id=461203>; modified from Horner et al., 2004, character 3): one (0); one functional tooth rostrally and caudally, and up to two teeth at and approaching the middle of the dental battery (1); three functional teeth throughout most of the dental battery, gradually decreasing to two near the rostral and caudal ends of the dentary (2).
4. Height/width ratio of the dentary tooth crowns in lingual aspect (DTTH5, <http://www.morphbank.net/Show/?id=461204>; Prieto-Márquez, 2010a, character 4): ratio up to 1.95 (sample mean ratio of 1.6) (0); ratio from 1.95 to 2.7 (sample mean ratio of 2.4) (1); ratio from 2.8 to 3.3 (sample mean ratio of 3.0) (2); ratio greater than 3.3 (sample mean ratio of 3.7) (3).
5. Maximum number of ridges on the enameled lingual side of dentary tooth crowns (DTTH6, <http://www.morphbank.net/Show/?id=461205>; modified Horner et al., 2004, character 6): presence of a primary major ridge extending from the ventral to the dorsal end of the crown, a rostral and slightly shorter secondary ridge and several (three or more) subdisidary, faintly developed and short tertiary ridges (0); presence of primary, secondary, and one or two tertiary ridges (1); presence of a primary ridge and one or two faint and shorter ridges (2); loss of all but the primary ridge (3).
6. Dentary tooth crowns, position of the primary ridge (DTTH7, <http://www.morphbank.net/Show/?id=461207>; modified from You et al., 2003, character 39): well offset caudally from the midline (0); median for most teeth, although some teeth within the same dental battery may display a slight caudal offset of the primary ridge (1).
7. Overall morphology of the dentary marginal denticles (DTTH10, <http://www.morphbank.net/Show/?id=461210>; Norman, 2002, character 31): wedge to tongue-shaped (0); curved and mammillated asymmetrical ledge (1); absent or very reduced to small papillae along the apical half of the dorsal half of the crown (2).
8. Denticle size (DTTH12, <http://www.morphbank.net/Show/?id=461212>; Prieto-Márquez, 2010a, character 11): the denticles of both mesial and distal margins are equal in size (0); the mesial margin has larger denticles than the distal one (1).
9. Imbrication of dentary tooth crowns (DTTH13, <http://www.morphbank.net/Show/?id=461212>; Prieto-Márquez, 2010a, character 12): absent (0); present, the mesial margin overlaps the distal one of the adjacent crown (1).
10. Maximum number of tooth positions in the maxillary dental battery (MXTH1, <http://www.morphbank.net/Show/?id=461215>; modified from Horner et al., 2004, character 1): up to 32 tooth positions (sample mean of 23 teeth) (0); from 33 to 44 tooth positions (sample mean of 40 teeth) (1); 45 or more tooth positions (sample mean of 49 teeth) (2).
11. Increase in the number of tooth positions in the maxilla relative to the dentary (MXTH3, <http://www.morphbank.net/Show/?id=461217>; modified from Wagner, 2001): absent (0); present, maxillary dental battery with 5 to 20% more tooth positions than the dentary one (1).
12. Maximum number of functional teeth per alveolus in the maxillary occlusal plane (MXTH4, <http://www.morphbank.net/Show/?id=461218>; modified from Horner et al., 2004, character 3): one (0); one tooth for most of the dental battery, with the sporadic presence of a second tooth forming the occlusal plane (1); two functional teeth throughout most of the dental battery length, gradually changing to one near the rostral and caudal ends of the maxilla (2).
13. Maximum number of ridges on the enameled labial side of maxillary tooth crowns (MXTH5, <http://www.morphbank.net/Show/?id=461219>; Horner et al., 2004 character 7): presence of a primary major ridge and three or more much fainter ridges (0); loss of all but the primary ridge in all or, at least, most of the crowns (in the latter situation a few crowns show a fainter secondary ridge) (1).
14. Maxillary tooth crowns, position of the primary ridge (MXTH6, <http://www.morphbank.net/Show/?id=461220> and <http://www.morphbank.net/Show/?id=461221>; modified from You et al., 2003 character 36): the dental battery contains a mixture of teeth with primary ridge positioned caudally and teeth with the ridge at the center of the crown (0); the majority of teeth in the dental battery have a primary ridge positioned at the midline of the crown (1).
15. Overall morphology of the maxillary marginal denticles (MXTH8, <http://www.morphbank.net/Show/?id=461223>; Norman, 2002, character 30): wedge to tongue-shaped (0); curved and mammillated asymmetrical ledge (1); absent or reduced to small papillae along the apical half of the dorsal half of the crown (2).

Mandibular Characters

1. Predentary. Ratio between the predentary maximum mediolateral width and the maximum rostrocaudal length along the lateral process (PDT1, <http://www.morphbank.net/Show/?id=461224>; modified from Horner et al., 2004, character 13): up to 1.75 (0); more than 1.75 (1).
2. Predentary. Orientation of the rostral surface relative to the dorsal margin of the lateral process (PDT3, <http://www.morphbank.net/Show/?id=461226>; modified from Horner et al., 2004, character 14): angle of 75º or greater (sample mean angle of 81º) (0); angle between 56º and 74º (sample mean angle of 66º) (1); angle between 40º and 55º (sample mean angle of 47º) (2); angle of 40º or less, gently rounded rostral surface (sample mean angle of 34º) (3).
3. Predentary. Shape of the denticles of the predentary oral margin (PDT4, <http://www.morphbank.net/Show/?id=461227>; modified from Horner et al., 2004, character 13): triangular and pointed (0); subrectangular to rectangular (1).
4. Predentary. Number of predentary denticles in adult individuals lateral to the median denticle (not included in the count) (PDT6, <http://www.morphbank.net/Show/?id=461228>, <http://www.morphbank.net/Show/?id=461229>, and <http://www.morphbank.net/Show/?id=461230>; Prieto-Márquez, 2010a, character 27): maximum of five (0); six or more (1).
5. Predentary. Extension of the predentary denticulate margin (PDT7, <http://www.morphbank.net/Show/?id=461228>, <http://www.morphbank.net/Show/?id=461229>, and <http://www.morphbank.net/Show/?id=461230>; Prieto-Márquez, 2010a, character 28): denticles extending into the lateral process (0); denticles limited to the rostral margin (1).
6. Predentary. Morphology of the predentary rostrolateral corner (PDT8, <http://www.morphbank.net/Show/?id=461228>, <http://www.morphbank.net/Show/?id=461229>, and <http://www.morphbank.net/Show/?id=461230>; modified from Horner et al., 2004, character 13): gently rounded and continuous with the lateral process, giving the predentary an arcuate dorsal profile (0); subsquared rostrolateral corner (1); subsquared, very broad and rostrolaterally projected (2).
7. Predentary. Development of a lateral shelf on the dorsal side of the predentary lateral process (PDT9, <http://www.morphbank.net/Show/?id=460672>, <http://www.morphbank.net/Show/?id=460673>, and <http://www.morphbank.net/Show/?id=460674>; modified from Wagner, 2001): absence of shelf, presence of a rostrocaudally short and shallow groove limited to the distal region of the lateral process, bounded by a tall lateral wall (0); short and shallow shelf limited to the laterocaudal region of the lateral process (1); short and well-incised shelf that is wider near the rostrolateral corner of the predentary (2); shelf extremely narrow mediolaterally and very long rostrocaudally (3); shelf rostrocaudally long, deeply incised and mediolaterally broad, forming half of the mediolateral breadth of the lateral process and becoming wider distally (4).
8. Predentary. Ridge on the dorsal lingual, keel-shaped process (PDT11, <http://www.morphbank.net/Show/?id=461232>; Prieto-Márquez, 2010a, character 31): the process lacks a prominent median ridge on the lingual side of the rostral region of the predentary, and, if present, the former forms and projects caudally from the caudal margin of the predentary rostral region (0); the process has a well-developed ridge on the lingual surface of the rostral segment of the predentary, from which the former extends further caudally to lie dorsal to the dentary symphysis (1).
9. Predentary. Ventral median process, degree of indentation of the split of the process into two distinct lobes (PDT13, <http://www.morphbank.net/Show/?id=461234>; Prieto-Márquez, 2010a, character 32): short indentation and deep undivided portion, the splitting originates at a distance from the predentary ventral margin that equals approximately half of the mediolateral width of the ventral process (0); long indentation and shallow undivided portion, the splitting originates at a distance from the predentary ventral margin that is less than the mediolateral width of the process (1).
10. Dentary. Ratio between the length of the proximal edentulous slope of the dentary and the distance between the rostralmost tooth position and the caudal margin of the coronoid process (DT1, modified from Prieto-Márquez, 2010a, character 33): less than 0.20 (0); ratio between 0.20 and less than 0.40 (1); 0.40 to 0.60 (2); greater than 0.60 (3).
11. Dentary. Angle of deflection of the rostral ventral margin of the dentary (DT4, modified from Prieto-Márquez, 2010a, character 36): angle up to 20º (0); angle greater than 20º (1).
12. Dentary. Location of the origination of the ventral deflection the dentary (measured as the ratio between the distance from the caudal margin of the coronoid process to the inflexion point of the ventral margin and the distance from the caudal margin the coronoid process to the rostralmost alveolus) (DT5, <http://www.morphbank.net/Show/?id=461239>; Prieto-Márquez, 2010a, character 37): the deflection occurs near the rostral end of the dentary, ratio greater than 0.78 (sample mean ratio of 0.87) (0); ratio between 0.66 and 0.78 (sample mean ratio of 0.72) (1); deflection originating near the middle of the dental battery, ratio of 0.65 or less (sample mean ratio of 0.59) (2).
13. Dentary. Orientation of the dentary symphysis (measured as the angle formed by this surface and the lateral side of the rostral half of the dentary) (DT7, <http://www.morphbank.net/Show/?id=461241>; Prieto-Márquez, 2010a, character 39): angle greater than 15º (sample mean angle of 23º) (0); angle up to 15º (sample mean angle of 10º) (1).
14. Dentary. Medial or lateral profile of the dorsal margin of the rostral edentulous region of the dentary for articulation with the predentary (DT9, <http://www.morphbank.net/Show/?id=461243>; Prieto-Márquez, 20¡10a, character 40): ranging from having a very subtle concavity (almost straight) to straight or even displaying a subtle convexity (0); having a well-pronounced concavity (1).
15. Dentary. Orientation of the long axis of the coronoid process relative to the dorsal margin of the alveolar sulci of the dental battery (DT11; modified from Godefroit et al., 2008, character 39): coronoid process subvertical or caudally inclined (0); process rostrally inclined (1).
16. Dentary. Morphology of the apex of the coronoid process (in adults) (DT12, <http://www.morphbank.net/Show/?id=461246>; modified from Horner et al., 2004, character 17): slightly expanded rostrocaudally, with very limited development of rostral and caudal expansions resulting in an apex that is taller than wider (0); well developed expansion of both the caudal and, especially, the rostral margins (1).
17. Dentary. Caudodorsal margin of the coronoid process projected dorsally into a sharp point (DT13, <http://www.morphbank.net/Show/?id=461246>; Prieto-Márquez, 2010a, character 44): absent (0); present (1).
18. Dentary. Thick and dorsoventrally elongated ridge on the medial side of the coronoid process, located near the caudal margin of the process (DT14, <http://www.morphbank.net/Show/?id=461247>; Prieto-Márquez, 2010a, character 45): absent, presence of fine striations (0); present, the ridge forms the rostral boundary of a depressed facet for attachment of the rostrodorsal process of the surangular; coarse striations present rostral to the ridge (1).
19. Dentary. Lateral expansion of caudal region of the dentary, ventral to the base of the coronoid process (measured as the angle between the lateral surface of the dentary and that of the region caudoventral to the coronoid process) (DT15, <http://www.morphbank.net/Show/?id=461248>; Prieto-Márquez, 2010a, character 46): the lateral side of the dentary is only slightly expanded laterally ventral to the coronoid process, with an angle greater than 165º (sample mean angle of 171º) (0); well developed expansion of the lateral side of the dentary ventral to the coronoid process, with an angle up to 165º (sample mean angle of 154º) (1).
20. Dentary. Orientation of the longitudinal axis of the dentary occlusal plane relative to the lateral side of the bone (as seen dorsally and caudal to the edentulous region) (DT16, <http://www.morphbank.net/Show/?id=461249>; Prieto-Márquez, 2010a, character 47): diagonal axis, directed rostrolaterally and forming approximately 15º with the lateral side of the dentary (0); axis parallel to the lateral side of the dentary (1).
21. Dentary. Lingual arching of the occlusal plane (DT17, <http://www.morphbank.net/Show/?id=461250>; Horner et al., 2004, character 12): present, lingually convex occlusal plane (0); absent, rostrocaudally straight occlusal plane (1).
22. Dentary. Caudal extension of the dental battery (DT18, <http://www.morphbank.net/Show/?id=461251>; modified from Horner et al., 2004, character 10): the caudal end of the dental battery is found rostral to the caudal margin of the coronoid process (0); the caudal end of the dental battery is found flush with the caudal margin of the coronoid process (1); the caudal end of the dental battery is found caudal to the caudal margin of the coronoid process (2).
23. Dentary. Separation between the dentary tooth row and the coronoid process (DT19,

<http://www.morphbank.net/Show/?id=461252>; Norman, 2002, character 26): the coronoid process is laterally offset (but nearly in contact) with the tooth row, lacking a platform in-between the tooth row and the base of the process (0); the coronoid process is laterally offset relative to the tooth row, with the presence of a concave platform or, in some cases, a laterodorsal concave slope separating the base of the process from the dental battery (1).

1. Surangular. Morphology of the rostrodorsal process of the surangular (SA1, <http://www.morphbank.net/Show/?id=461253>; modified from Wagner, 2001): rostrocaudally thick process, slightly reduced in thickess rostrally, extensively exposed in lateral view (0); rostrocaudally reduced in thickness, strap-like and wedging dorsally into a thin sliver that becomes concealed in lateral view by the dorsal half of the caudal margin of the coronoid process (1).
2. Surangular foramen (SA2, <http://www.morphbank.net/Show/?id=461254>; Norman, 2002, character, character 27): present (0); absent (1).
3. Surangular. Accessory foramen located rostrodorsal to the main surangular foramen (SA3, <http://www.morphbank.net/Show/?id=461255>; Kobayashi and Azuma, 2003, character 15): present (0); absent (1).
4. Surangular. Orientation of the convex side of the lateral lap and the lateroventral surface of the main body of the surangular (SA4, <http://www.morphbank.net/Show/?id=461256>; Prieto-Márquez, 2010a, character 54): facing more laterally than ventrally (0); facing more ventrally than laterally (1).
5. Surangular. Lateral curvature of the caudal process of the surangular (SA5, <http://www.morphbank.net/Show/?id=461257>; Prieto-Márquez, 2010a, character 55): absent, process nearly straight rostrocaudally (0); present, process laterally recurved (1).
6. Angular. Position of the angular in the mandible (ANG, <http://www.morphbank.net/Show/?id=461259>; Weishampel, Norman and Grigorescu, 1993, character 26): positioned ventrally and slightly medially, exposed in lateral view (0); positioned medially, not exposed in lateral view (1).
7. Prearticular bone (PRAR, <http://www.morphbank.net/Show/?id=461261>; Prieto-Márquez, 2010a, character 59): absent (0); present (1).

Facial Characters

1. Premaxilla. Mediolateral expansion of the premaxillary oral margin (measured as the ratio between the maximum mediolateral width of the premaxilla and the minimum width at the narrowest point or post-oral constriction) (PMX1, <http://www.morphbank.net/Show/?id=461264>; modified from Horner et al., 2004, character 22): relatively narrow, ratio less than 1.65 (mean ratio of 1.45) (0); ratio between 1.65 and 2 (mean ratio of 1.84) (1); very wide, with a ratio greater than 2 (mean ratio of 2.22) (2).
2. Premaxilla. Position of the premaxillary oral margin relative to the occlusal plane of the dentition (PMX2, <http://www.morphbank.net/Show/?id=461265>; modified from Norman, 2002): premaxillary margin slightly ventrally offset from occlusal plane (approximately, the dorsoventral distance between the occlusal plane and the level of the premaxillary oral margin is less than the mean depth of the dentary) (0); very strongly deflected ventrally (approximately, the dorsoventral distance between the occlusal plane and the level of the premaxillary oral margin is equal to or larger than the mean depth of the dentary) (1).
3. Premaxilla. Degree of expansion and folding of the oral margin of the premaxilla (modified from Horner et al., 2004, character 22): moderately expanded border, dorsoventrally thicker towards the parasagittal plane of the snout, and slightly deflected ventrally (PMX3, <http://www.morphbank.net/Show/?id=461266>) (0); moderately expanded border, becoming thinner towards the parasagittal plane of the snout (<http://www.morphbank.net/Show/?id=461267>) (1); folded caudodorsally into a thin recurved margin (<http://www.morphbank.net/Show/?id=461268>) (2); ventrally deflected and dorsoventrally expanded, forming a very broad ‘lip-like’ margin (<http://www.morphbank.net/Show/?id=461269>) (3).
4. Premaxilla. Premaxillary oral margin with a ‘double layer’ morphology consisting of an external denticle-bearing layer and an internal layer of thickened bone set back slightly from the oral margin and separated from the denticular layer by a deep sulcus bearing vascular foramina (PMX5, <http://www.morphbank.net/Show/?id=461271>; Horner et al., 2004, character 25): absent (0); present (1).
5. Premaxilla. Circumnarial depression including a premaxillary rostral fossa set rostral to the circumnarial fossa proper (PMX8, <http://www.morphbank.net/Show/?id=461274>; Horner et al., 2004, character 26): absent (0); present, separated from circumnarial depression by a rostrocaudally wide ridge (1).
6. Premaxilla. Accessory rostral fossa located lateral to the rostral fossa and rostrolateral to the circumnarial fossa, parallel to the lateral border of the oral margin (PMX9, <http://www.morphbank.net/Show/?id=461275>): absent (0); present (1).
7. Premaxilla. Premaxillary foramen located rostrally and ventrolateral to the rostral margin of the narial foramen (PMX6, <http://www.morphbank.net/Show/?id=461272>; Horner et al., 2004, character 23): absent (0); present (1).
8. Premaxilla. Premaxillary accessory foramen entering rostrally through the rostral fossa, located rostral to the premaxillary foramen (PMX7, <http://www.morphbank.net/Show/?id=461273>; Horner et al., 2004, character 24): absent (0); present, empties into a common chamber with the premaxillary foramen (1).
9. Premaxilla. Elongation of premaxillary medial process (PMX10, <http://www.morphbank.net/Show/?id=461276>; modified from Horner et al., 2004, characters 27): the premaxillary medial process does not meet the lateral process caudally (0); elongate medial process that extends caudally to meet the lateral process, forming the caudal margin of the external bony naris (1).
10. Premaxilla. Vertical groove on the lateral process of the premaxilla, located rostral to the medial process of the maxilla and extending ventrally from a small opening between the two premaxillary processes; the groove is bounded rostrally by a triangular ventral projection of the lateral process of the premaxilla (PMX11, <http://www.morphbank.net/Show/?id=461277>; Evans and Reisz, 2007, character 5): absent (0); present (1).
11. Premaxilla. Elongation of the lateral process of the premaxilla (in adults) (PMX12, <http://www.morphbank.net/Show/?id=461278>; modified from Suzuki, Weishampel and Minoura, 2004, character 4): relatively short, the lateral process extends caudodorsally to end dorsal to the lacrimal or medidodorsal to the rostral end of the prefrontal (0); long, the lateral process extends to end medial to the dorsal region of the prefrontal (1); very long, the lateral process extends caudodorsal to the prefrontal (2).
12. Premaxilla. Morphology of the caudal region of the lateral process of the adult premaxilla: mediolaterally compressed and triangular (PMX13, <http://www.morphbank.net/Show/?id=461279>; Prieto-Márquez, 2010a, character 71) (0); dorsoventrally broad and directed caudally or caudally and slightly dorsally (<http://www.morphbank.net/Show/?id=461280>) (1); triangular and dorsoventrally expanded, laterally convex lobe, directed rostrodorsally (<http://www.morphbank.net/Show/?id=461281>) (2).
13. Premaxilla. Premaxillary medial process has an accessory rostroventral flange that overlaps the lateral surface of the nasal in the rostral region of a supracranial crest (PMX14, <http://www.morphbank.net/Show/?id=461282>); Evans and Reisz, 2007, character 18): absent (0); present (1).
14. Premaxilla. Laterodorsal profile of the caudodorsal and caudoventral margins of the external bony naris (PMX15, <http://www.morphbank.net/Show/?id=461283> and <http://www.morphbank.net/Show/?id=461285>; modified from Evans and Resiz, 2007, character 4): subrectangular to subellipsoidal (0); triangular and very long (length/width ratio greater than 2.85), caudal constriction gradually closing caudodorsally (1); triangular and moderately long (length/width ratio greater than 2.85), caudal constriction gradually closing caudodorsally (length/width ratio between 1.85 and 2.85) (2); lacriform (length/width ratio less than 1.85), caudal constriction occurs abruptly and is primarily composed of a lateroventral expansion of the medial premaxillary process (3); lacriform (length/width ratio less than 1.85), caudal constriction occurs abruptly and is primarily composed of a dorsal expansion of the lateral process of the premaxilla (4).
15. Premaxilla. Dorsolateral flange at approximately mid-length of the mediolaterally compressed lateral process of the premaxilla (PMX16, <http://www.morphbank.net/Show/?id=461286>; Gates and Sampson, 2007): absent (0); present (1).
16. Premaxilla. One or more foramina on the rostromedial surface of the premaxilla (PMX17; Prieto-Márquez, 2010b, character 287): absent (0); present (1).
17. Premaxilla. Lateral profile of the dorsal margin of the rostral region of the rostrum (PMX18; Prieto-Márquez and Wagner, in press, character 287): convex (0); straight to gently concave (1); strongly concave (2).
18. Premaxilla. Contour of the rostrolateral region of the thin everted oral margin (PMX19; Prieto-Márquez and Wagner, in press, character 289): broad and arcuate (0); subangular (1).
19. Premaxilla. Orientation of the medial process relative to the lateral process around the narial foramen (PMX20, new): subparallel (0); processes slightly converging caudally (1).
20. Nasal. Location of the nasal bone and nasal cavity in the adult skull (NS1, <http://www.morphbank.net/Show/?id=461287>; modified from Horner et al., 2004, character 33 and partially from Evans and Reisz, 2007, character 7): the nasal extends from the rostral region of the skull roof to the rostrodorsal region of the snout with the nasal cavity rostromedial to the orbit (0); nasal retracted caudal to the rostrum and occupying a supracranial position in the skull, with the ventral region of the nasal meeting the prefrontal rostral to the orbit, resulting in a crest that extends supraorbitally (1); retracted caudal to the rostrum and occupying a supracranial position in the skull, with the ventral region of the nasal meeting the prefrontal caudal to the rostral margin of the orbit, resulting in a convoluted narial passage and hollow crest that extend supraorbitally (2).
21. Nasal. Curvature of the caudodorsal region of the nasal (NS2, <http://www.morphbank.net/Show/?id=461288>; modified from Wagner, 2001 and partially from Evans and Reisz, 2007, character 7): absent, nasal straight caudodorsally (0); present, nasal rotated and folded caudodorsally (1).
22. Nasal. Morphology of the rostral end of the nasal dorsal process at the contact with the medial process of the premaxilla (NS3, <http://www.morphbank.net/Show/?id=461289> and <http://www.morphbank.net/Show/?id=461290>; states 3 and 4 modified from Evans and Reisz, 2007, character 17): long and wedge-shaped rostral process, gradually decreasing in width rostrally to a sharp point (0); hook-like process, it becomes abruptly deep near its rostral end and then wedges rostrally to a rostroventrally directed (1); long and subrectangular process, with slightly rounded corners (2); small rostral process of the nasal fits along the ventral edge of the premaxilla, the latter briefly overlapping the nasal (3); the nasal bifurcates to meet the premaxilla in a W-shaped interfingering suture, a long and finger-like process of the nasal has an extensive overlapping joint with the medial process of the premaxilla; and additional, more caudally located shorter process of the nasal abuts the premaxilla (4).
23. Nasal. Morphology of the nasal contact with the caudodorsal region of the medial premaxillary process at the caudal margin of the narial foramen (NS4, <http://www.morphbank.net/Show/?id=461291>; Prieto-Márquez, 2010a, character 78): the nasal forms a subrectangular flange exposed dorsal to the premaxillary caudoventral process (0); the nasal forms a large hook-like rostroventral process, exposed dorsal to the premaxillary caudoventral process (1); the nasal forms a greatly shortened and dorsoventrally narrow hook-like rostroventral process, exposed dorsal to the premaxillary caudoventral process (2).
24. Nasal. Location of the rostral end of the dorsal process of the nasal relative to the rostral margin of the narial foramen (NS5, <http://www.morphbank.net/Show/?id=461292>; Prieto-Márquez, 2010a, character 79): the rostral end of the dorsal process of the nasal does not reach the rostral margin of the narial foramen (0); the rostral end of the rostrodorsal process of the nasal reaches the rostral margin of the narial foramen (1).
25. Nasal. Caudoventral region of nasal, in hollow supracranial crest, ventrally recurved and hook-shaped, with a rostral process that inserts under the caudoventral process of the premaxilla (NS6, <http://www.morphbank.net/Show/?id=461293>; Prieto-Márquez, 2010a, character 80): absent (0); present (1).
26. Nasal. Caudal end of the nasals forming a pair of finger-like process on top of the frontals and centered around the sagittal plane of the skull roof (NS7, <http://www.morphbank.net/Show/?id=461294>; Gates and Sampson, 2007, character 65, in part): absent (0); present (1).
27. Nasal. Caudal end of nasals forming a pair of processes that insert between the frontals at the sagittal plane of the skull roof (NS8, <http://www.morphbank.net/Show/?id=461295>; Gates and Sampson, 2007, character 65, in part): absent (0); present (1).
28. Nasal. Mediolateral breath of caudal nasal processes that insert in between the frontals at sagittal plane of the skull (N10; new): processes broad and converging caudally in width, forming a V-shaped dorsal outline between the frontals (0); processes greatly compressed mediolaterally and finger-like (1).
29. Nasal. Position of the summit of nasal arch crest relative to the caudodorsal margin of the narial foramen (NS9, <http://www.morphbank.net/Show/?id=461296>; Prieto-Márquez, 2010, character 83): summit located dorsal to the caudal margin of the narial foramen (0); summit located caudodorsal to the caudal margin of the narial foramen (1).
30. Maxilla. Rostrodorsal process that is medially offset from the body of the maxilla and extends also medial to the caudoventral process of premaxilla to form part of medial floor of external naris (MX1, <http://www.morphbank.net/Show/?id=461297>; partially after Wagner, 2001 and Horner et al., 2004, character 42): absent, the rostral end of the maxilla forms a ventrally sloping rostrodorsal shelf that underlies the premaxilla (0); present (1).
31. Maxilla. Lateral exposure of the rostrodorsal process (MX2, <http://www.morphbank.net/Show/?id=461298>; modified from Gates and Sampson, 2007, character 45): not exposed or only the distal tip exposed through the narial foramen in lateral view (0); large segment of the process exposed through the narial foramen in lateral view (1).
32. Maxilla. Angle between the dorsal margin of the rostroventral process or shelf of the maxilla and the rostral segment of the tooth row (MX4; modified from Prieto-Márquez, 2010a, character 87): rostrodorsal region of the maxilla subconical in shape, dorsoventrally narrow, forming an angle of 25º or less with the rostral tooth row (0); dorsoventrally thicker, forming an angle greater then 25º and up to 35º with the rostral tooth row (1); rostroventral process dipping steeply ventrally, forming an angle greater than 35º with the tooth row, rostral region of the maxilla appearing dorsally ‘swollen’ and rostrocaudally compressed (2).
33. Maxilla. Morphology of the laterally exposed surface of the rostrodorsal region of the maxilla, adjacent and rostral to the jugal articular surface (MX5; modified from Prieto-Márquez, 2010a, character 88): subarcuate profile (0); tall and sharply triangular to finger-shaped (1); subtrapezoid to subrectangular, with horizontal dorsal margin under lacrimal (2).
34. Maxilla. Rostrocaudal extension of the laterally exposed surface of the rostrodorsal region of the maxilla (MX21): moderate to extensive lateral exposure under lacrimal (0); greatly reduced rostrocaudally to a very narrow surface (1).
35. Maxilla, rostrodorsal margin bearing a prominent subrectangular flange that rises vertically above the rostroventral process (MX19): absent (0); present (1).
36. Maxilla. Position of the dorsal process and the dorsal margin of the dorsolateral promontory of the maxilla (expressed as the ratio between the distance from its summit to the rostral end of the maxilla and the rostrocaudal length of the element) (MX7, <http://www.morphbank.net/Show/?id=461303>; Prieto-Márquez, 2010a, character 90): caudally located dorsolateral promontory (with a ratio greater than 0.57; mean of 0.64), base of dorsal process positioned within the caudal third of the maxilla (0); centrally located dorsolateral promontory (with a ratio between 0.47 and 0.57; mean of 0.51), base of dorsal process positioned slightly caudal to the mid-length of the maxilla (1); dorsolateral promontory located slightly rostral to the mid-length of the maxilla (with a ratio between 0.35 and 0.46; mean of 0.42), base of dorsal process centered around the mid-length of the bone (2); the base of dorsal process and dorsolateral promontory located rostral to the mid-length of the maxilla, with a ratio less than 0.35 (mean of 0.28) for the relative position of the rostrodorsal promontory (3).
37. Maxilla. Morphology of the apex of the dorsal process of the maxilla (MX8, <http://www.morphbank.net/Show/?id=461304>; modified from Horner et al., 2004, character 48): subtriangular, not dorsoventrally taller than it is rostrocaudally wide (0); dorsoventrally taller than it is wide, with a peaked and caudally inclined apex (1).
38. Maxilla. Morphology of the articulation surface for the jugal (MX9, <http://www.morphbank.net/Show/?id=461305>; modified from Prieto-Márquez, 2010a, character 92): protruding lateral to the caudal third of the maxilla as a mediolaterally compressed finger-like process directed caudolaterally, separated a short distance from the lateral side of the element (0); consisting on a promontory located dorsal and rostral to the ectopterygoid shelf, bearing a concave and subtriangular, dorsolaterally-facing joint surface for the jugal, wit (<http://www.morphbank.net/Show/?id=461306>) (1); subtriangular joint surface for the jugal that is more laterally than dorsally-facing, adjacent and slightly dorsal to the proximal end of the lateral ridge of the ectopterygoid shelf (<http://www.morphbank.net/Show/?id=461307>) (2); dorsally elevated jugal joint such that ectopterygoid ridge is discontinuous with, and ends below, articular surface for jugal (<http://www.morphbank.net/Show/?id=461308>) (3).
39. Maxilla. Acute embayment betweem jugal facet and ectopterygoid shelf, caused by continuation of ectopterygoid ridge into ascending border that curves dorsocaudally: absent (0); present (1).
40. Maxilla. Arrangement of maxillary foramina ventral and rostral to the jugal articulation (excluding large rostrodorsal or rostrolateral foramen): positioned rostrocaudally and scattered throughout the lateral side of the maxilla (MX10, <http://www.morphbank.net/Show/?id=461309>; Prieto-Márquez, 2010a, character 93) (0); forming either a row or cluster that is oriented rostrodorsally (<http://www.morphbank.net/Show/?id=461310>, <http://www.morphbank.net/Show/?id=461311>, and <http://www.morphbank.net/Show/?id=461312>) (1).
41. Maxilla. Number of maxillary foramina ventral and rostral to the jugal articulation (excluding large rostrodorsal or rostrolateral foramen) (MX11, <http://www.morphbank.net/Show/?id=461313>; Prieto-Márquez, 2010a, character 94): seven or more (0); six or less (1).
42. Maxilla. Large rostral maxillary foramen (MX12, <http://www.morphbank.net/Show/?id=461314> and <http://www.morphbank.net/Show/?id=461315>; Evans and Reisz, 2007, character 22 modified after Horner et al., 2004, character 44): opening on the rostrolateral body of the maxilla, within the rostral half of the rostrodorsal margin of the element and exposed in lateral view (0); opening on the rostrolateral body of the maxilla, within the dorsal half of the rostrodorsal margin of the element and exposed in lateral view (1); opening on the dorsal surface of the maxilla along the maxilla-premaxilla contact, not exposed laterally (2).
43. Maxilla-lacrimal contact (MX13, <http://www.morphbank.net/Show/?id=461316> and <http://www.morphbank.net/Show/?id=461317>; Evans and Reisz, 2007, character 23): present externally (0); largely covered externally by the jugal-premaxilla contact (1).
44. Maxilla. Length of the ectopterygoid shelf relative to the total rostrocaudal length of the alveolar margin of the maxilla (MX14, <http://www.morphbank.net/Show/?id=461318>; partially after You et al., 2003, character 12): ratio between the length ectopterygoid shelf and the length of the rostrocaudal alveolar margin up to 0.25 (mean ratio of 0.20) (0); ratio greater than 0.25 and up to 0.35 (mean ratio of 0.30) (1); ratio greater than 0.35 (mean ratio of 0.45) (2).
45. Maxilla. Slope of the ectopterygoid shelf, measured as the angle between this and the rostrocaudal axis of the caudal segment of the tooth row (MX15, <http://www.morphbank.net/Show/?id=461319>; Prieto-Márquez, 2010a, character 98): steeply inclined caudoventrally, with an angle greater than 21º (mean angle of 29º) (0); shelf inclined with an angle greater than 10 and up to 21º (mean angle of 15º) (1); slightly inclined shelf, with an angle greater than 4º and up to 10º (mean angle of 8º) (2); horizontal shelf, with an angle up to 4º (3).
46. Maxilla. Morphology of the lateral emargination of the ectopterygoid shelf (MX16, <http://www.morphbank.net/Show/?id=461320>; modified from Godefroit, Zan and Jin, 2000, character 14): dorsoventrally thin ridge (0); faint or dorsoventrally thin rostrally, then abruptly becoming dorsoventrally thick along the caudal segment of the margin (1); dorsoventrally thick continuous ridge, gradually thicker caudally than rostrally (2).
47. Maxilla. Position of the central region of the arcuate row of special foramina on the medial side of the maxilla (MX17, <http://www.morphbank.net/Show/?id=461321>; Prieto-Márquez, 2010a, character 100): ventral to or at the level of the mid-dorsoventral depth of the maxilla (0); dorsal to the mid-dorsoventral depth of the maxilla (1).
48. Lacrimal. General morphology of the adult lacrimal in lateral view (LC1, <http://www.morphbank.net/Show/?id=461325>; Prieto-Márquez, 2010a, character 101): triangular and rostrocaudally elongated, with a rostral process that is rostrally (and slightly ventrally) directed (0); triangular, rostrocaudally abbreviated with a relatively shorter and thinner rostral process (1).
49. Lacrimal. Ventral margin of the lacrimal with a prominent convexity rostral to the jugal notch (LC2, <http://www.morphbank.net/Show/?id=461326>; Prieto-Márquez, 2010a, character 102): absent (0); present (1).
50. Jugal. Rostral apex of the rostral process of the jugal (J1, <http://www.morphbank.net/Show/?id=461327> and <http://www.morphbank.net/Show/?id=461328>; Prieto-Márquez, 2010a, character 103): present, wedge-shaped, elongated and sharply pointed, positioned at mid distance along the dorsoventral depth of the rostral process (0); present, wedge-shaped, pointed and less elongated than in (0), positioned within the dorsal half of the rostral process of the jugal; the dorsal magin of the apex forms a steeper angle with the horizontal than in state (0) (1); greatly reduced to a blunt convexity (2); reduced to a short process, only slightly thinner rostrally and ending abruptly (3); absent, straight nearly vertical rostral margin (4); absent, tongue-shaped rostral margin (5).
51. Jugal. Dorsoventral expansion of the caudodorsal margin of the rostral process of the jugal (J2, <http://www.morphbank.net/Show/?id=461329>; Weishampel et al., 1993, character 15): dorsoventrally narrow, rostrodorsally directed and forming little of the rostroventral margin of the orbital rim (0); dorsoventrally deep (about 60-90% as deep as the rostral jugal constriction), dorsally or slightly recurved caudodorsally, forming the rostroventral corner of the orbital rim (1).
52. Jugal. Morphology of the triangular caudoventral margin of the rostral process of the jugal (J3, <http://www.morphbank.net/Show/?id=461330>; Prieto-Márquez, 2010a, character 105): shallow and rostrocaudally wide prominence (wider than deep) (0); ventrally pointed, approximately as deep as or slightly deeper as its proximal end is wide (1); ventrally projected triangular narrow process, at least twice as deep as it is wide, sharply pointed and often recurved caudally (2).
53. Jugal. Location of the caudoventral apex of the rostral process relative to the caudodorsal articulation with the lacrimal (with longitudinal axis of the rostral process oriented horizontally) (J4, <http://www.morphbank.net/Show/?id=461333>; Prieto-Márquez, 2010a, character 106): apex located caudoventral to the caudal margin of the lacrimal process (0); apex located ventral to the caudal margin of the lacrimal process (1).
54. Jugal. Orientation of the medial articular surface of the rostral process of the jugal (J5, <http://www.morphbank.net/Show/?id=461332>; Prieto-Márquez, 2010a, character 107): facing medioventrally, the articular surface forms a deep concavity bounded dorsally and caudally by a laterally offset rim (0); facing medially, the articular surface is bounded only caudally by a rim of bone (1).
55. Jugal. Ventral expansion of the caudoventral jugal flange (measured as the ratio between the dorsoventral depth of the flange and the minimum depth of the caudal constriction of the jugal) (J8, <http://www.morphbank.net/Show/?id=461336>; modified from Wagner, 2001): slightly expanded flange, ratio of 1.36 or less (mean ratio of 1.29) (0); moderately expanded flange, ratio greater than 1.36 and up to 1.55 (mean ratio of 1.44) (1); greatly expanded flange, ratio greater than 1.55 (mean ratio of 1.68) (2).
56. Jugal. Lateral profile of the quadratojugal flange (J9, <http://www.morphbank.net/Show/?id=461337> and <http://www.morphbank.net/Show/?id=461338>; Prieto-Márquez, 2010a, character 111) subconical, dorsoventrally tall and rostrocaudally narrow, with a nearly vertical caudal margin (0); auricular in shape, with subparallel concave to nearly straight dorsal and convex ventral margins that converge dorsally into a short subconical point (1); fan-like, with dorsal and ventral margins that are subparallel and diverge caudodorsally; dorsal and ventral margins can be straight or slightly bowed dorsally (2); auricular in shape, with subparallel concave to nearly straight dorsal and convex ventral margins that converge dorsally into a recurved or dorsally directed tall subconical extension (this state is similar to (1), but the dorsal region of the flange is rostrocaudally narrower and taller) (3).
57. Jugal. Morphology of the ventral margin located between the caudoventral and quadratojugal flanges (J10, <http://www.morphbank.net/Show/?id=461339>; modified from Weishampel et al., 1993, character 18): relatively short and shallow concavity (0); relatively wide and well-pronounced concavity (1).
58. Jugal. Relative depth of the caudal and rostral constrictions (in adults) (rostral constriction region located between the rostral and postorbital processes; caudal constriction region located between the postorbital process and the caudoventral flange) (J11, <http://www.morphbank.net/Show/?id=461340>; Prieto-Márquez, 2010a, character 113): deeper rostral constriction, ratio of the depth of the caudal constriction relative to the rostral of 1 or less (mean ratio of 0.93) (0); deeper caudal constriction, with a ratio greater than 1 and less than 1.35 (mean ratio of 1.18, resulting from merging two K-means clusters with mean ratios of 1.13 and 1.24) (1); extremely deep caudal constriction, with a ratio greater than 1.35 (mean ratio of 1.43) (2).
59. Jugal. Relative width and lateral profiles of the orbital and infratemporal margins of the jugal (J13, <http://www.morphbank.net/Show/?id=461342>; Prieto-Márquez, 2010a, character 115): wider orbital margin and relatively constricted ventral margin of the infratemporal fenestra (0); orbital and infratemporal margins are nearly equally wide (1); wider infratemporal margin (2).
60. Quadrate. Degree of curvature of the caudal margin of the quadrate (Q1, <http://www.morphbank.net/Show/?id=461343>; Prieto-Márquez, 2010a, character 116): the caudal margin of the dorsal half or third of the quadrate displays a slight curvature relative to the ventral half of the element, with an angle of 150º or greater (mean angle of 161º) (0); the caudal margin of the dorsal half or third of the quadrate is strongly curved caudally relative to the ventral half of the element, with an angle less than 150º (mean angle of 143º) (1).
61. Quadrate. Position of the quadratojugal (paraquadrate) notch along the dorsoventral length of the quadrate (measured as the ratio between the distance from the mid-length of the notch to the qudadrate head and the dorsoventral length of the element) (Q2, <http://www.morphbank.net/Show/?id=461344>; Prieto-Márquez, 2010a, character 117): the mid point of the notch is located near the mid-length of the quadrate, ratio less than 0.60 (mean ratio of 0.54) (0); the mid point of the notch is located ventral to the mid-length of the quadrate, ratio of 0.60 or greater (mean ratio of 0.64) (1).
62. Quadrate. Orientation of the dorsal margin of the quadratojugal notch of the quadrate (measured as the angle between this and the caudal margin of the element) (Q3, <http://www.morphbank.net/Show/?id=461345>; Prieto-Márquez, 2010a, character 118): angle greater than 45º (mean angle of 52º) (0); angle up to 45º (mean angle of 28º) (1).
63. Quadrate. Morphology of the lateral profile of the quadratojugal notch of the quadrate (Q4, <http://www.morphbank.net/Show/?id=461346>; Prieto-Márquez, 2010a, character 119): subcircular, with a ventral half of the notch that is recurved and has a horizontal rostral segment (0); wide arcuate and asymmetrical, with the ventral half of the notch having a short horizontal rostral segment (1); wide arcuate and symmetrical, the ventral half of the notch being rostroventrally-directed, nearly straight as it is the dorsal half (2).
64. Quadrate. Development of the squamosal buttress on the caudal margin of the dorsal end of the quadrate (Q5, <http://www.morphbank.net/Show/?id=461347>; Prieto-Márquez, 2010a, character 120): absent or poorly developed as a gentle convexity (0); present, the buttress is a sharp protuberance hanging from the caudal side of the dorsal fourth of the quadrate, near the head of the element (1).
65. Quadrate. Morphology of ventral surface of the quadrate (Q6, <http://www.morphbank.net/Show/?id=461349> and <http://www.morphbank.net/Show/?id=461348>; Weishampel et al., 1993, character 22): mediolaterally broad and rostrocaudally compressed, lateral condyle slightly larger than the medial one (mean ratio between the rostrocaudal width of the lateral condyle and the mediolateral width of the ventral end of the quadrate of 0.59); the ventral surface of the lateral condyle is only slightly offset ventrally relative to the ventral surface of the medial condyle (0); subtriangular in ventral view, lateral condyle rostrocaudally expanded and much larger than the medial one (mean ratio between the rostrocaudal width of the lateral condyle and the mediolateral width of the ventral end of the quadrate of 0.90); the ventral surface of the lateral condyle is well offset ventrally relative to the ventral surface of the medial condyle (1).
66. Prefrontal. Dorsomedial margin of the prefrontal developed into a caudodorsally-oriented crest (PF1, <http://www.morphbank.net/Show/?id=461350> and <http://www.morphbank.net/Show/?id=461351>; Godefroit, Alifanov and Bolotsky, 2004, character 16): absent (0); present, not extending caudal to the prefrontal-frontal articulation (1); present, the crest extends caudally over the dorsal surface of the frontal and above the prefrontal-postorbital articulation in lateral view in adults (2).
67. Prefrontal. Lateral profile of the rostrodorsal margin of the prefrontal (PF2, <http://www.morphbank.net/Show/?id=461352>; modified from Horner et al., 2004, character 50): subarcuate to smoothly curved, the rostral margin is rostroventrally oriented and forming an obtuse angle with the dorsal orbital margin (0); rostromedially broad with subsquared rostrodorsal corner (1).
68. Prefrontal. Deep fossa on the ventral surface of the rostrodorsal corner of the orbit, rostrodorsal orbital margin being squared and slightly projected rostrodorsally (PF7; after Campione and Evans, 2011): absent (0); present (1).
69. Prefrontal. Mediolateral breadth of the exposed rostroventral region of the prefrontal (PF3, <http://www.morphbank.net/Show/?id=461353>; partially after Horner et al., 2004, character 50): the rostroventral region is mediolaterally expanded (0); the exposed rostroventral region is mediolaterally compressed and narrow (1).
70. Prefrontal. Inclusion of the prefrontal in the circumnarial fossa (PF4, <http://www.morphbank.net/Show/?id=461354> and <http://www.morphbank.net/Show/?id=461355>; Wagner, 2001): absent (0); present (1).
71. Prefrontal. Exposure of the prefrontal-nasal contact in lateral and/or dorsal view (PF6, <http://www.morphbank.net/Show/?id=461357>; modified from Wagner, 2001): contact totally exposed in lateral and/or dorsal view (0); contact visible in lateral view along the caudal and half of the dorsal margin of the prefrontal (1); contact visible in lateral view only along the caudal region of the prefrontal in adults, due to the invasion of the premaxilla along the medial side of the prefrontal (2).
72. Postorbital. Dorsal promontorium on the rostral process of the postorbital (PO1, <http://www.morphbank.net/Show/?id=461359> and <http://www.morphbank.net/Show/?id=461358>; Godefroit et al., 2004, character 17): absent, the dorsal surface of the postorbital above the jugal process is horizontal or slightly concave (0); present in adult specimens, the articular margin for the prefrontal is elevated and the dorsal surface of the postorbital above the jugal process is deeply depressed (1).
73. Postorbital. Rostrocaudal constriction of the dorsal region of the infratemporal fenestra (PO2, <http://www.morphbank.net/Show/?id=461360>; modified from Evans and Reisz, 2007, character 36): absent, caudal (squamosal) process of the postorbital elongate over the infratemporal fenestra (broad and subrectangular dorsal region of the fenestra) (0); present and caused by the presence of a nearly straight and oblique caudoventral margin of the caudodorsal region of the postorbital (dorsal region of infratemporal fenestra typically subtriangular) (1); present and caused by rostrocaudal shortening of the caudal process of the postorbital (dorsal region of infratemporal fenestra typically oval) (2).
74. Postorbital. Morphology of the central body of the postorbital (PO3, <http://www.morphbank.net/Show/?id=461361>; Prieto-Márquez, 2010a, character 130): triangular, rostrocaudally broad, expanded rostroventrally to form a straight and obliquely oriented caudodorsal orbital margin (0); triangular, with a caudodorsal orbital margin that ranges in lateral profile from semicircular to subsquared (1); rostrocaudally expanded, rostrally excavated and and bulging laterally (‘inflated’), containing a hollow inner cavity (in adults) (2).
75. Postorbital. Morphology of the caudal end of the caudal process of the postorbital at its articulation with the squamosal (PO5, <http://www.morphbank.net/Show/?id=461363>; Evans and Reisz, 2007, character 35): oblong or wedge-shaped (0); bifid (1).
76. Postorbital. Caudal extension of the caudal ramus of the postorbital that overlaps the laterodorsal surface of the squamosal (PO6, <http://www.morphbank.net/Show/?id=461364>; modified from Godefroit et al., 2000): the caudal end of the postorbital caudal ramus extends to a point rostral to the quadrate cotylus and does not overlap the latter (0); the caudal end of the postorbital caudal ramus extends caudodorsal to the precotyloid process and over as much as the rostral half of the quadrate cotylus (1); the caudal end of the postorbital caudal ramus completely overlaps the laterodorsal side of the squamosal quadrate cotylus (2).
77. Squamosal. Length of the precotyloid process of the squamosal (measured as the ratio of its length relative to the width of the quadrate cotylus) (SQ1, <http://www.morphbank.net/Show/?id=461365>; Prieto-Márquez, 2010a, character 134): very short precotyloid process, ratio less than 0.95 (mean ratio of 0.74) (0); moderately long precotyloid process, ratio between 0.95 and 1.25 (mean ratio of 1.13) (1); very long precotyloid process, ratio greater than 1.25 (mean ratio of 1.41) (2).
78. Squamosal. Dorsoventral expansion of the caudolateral surface of the squamosal (SQ2, <http://www.morphbank.net/Show/?id=461366>; Horner et al., 2004, character 64): unexpanded, shallowly exposed in caudal view (0); greatly expanded dorsomedially, forming a deep, near vertical, well-exposed face in caudal view (in adults) (1).
79. Squamosal. Separation of the squamosals at the occipital margin of the skull roof (SQ3, <http://www.morphbank.net/Show/?id=461367>; Horner et al., 2004, character 63): completely separated by the parietal (0); the squamosal approach the sagittal plane of the skull, separated by a narrow band of parietal (1); extensive intersquamosal joint present at the midline, parietal completely excluded from the sagittal plane of the skull at that particular spot (in adults) (2).
80. Squamosal. Rostromedial indenture of the medial ramus of the squamosal (SQ4, <http://www.morphbank.net/Show/?id=461368>; modified from Godefroit et al., 1998): absent, medial ramus of the squamosal extends medially, forming a subsquared caudolateral border of the skull roof (0); present, medial ramus of the squamosal curves rostromedially, so that the back of the skull appears to be deeply indented rostrally when viewed dorsally (1).

Neurocranial and Palatal Characters

1. Frontal. Bifurcation of the rostromedial margin of the frontals at the sagittal plane of the skull roof, leaving a V-shaped space in between (F1, <http://www.morphbank.net/Show/?id=461369>; Prieto-Márquez, 2010a, character 138): absent (0); present (1).
2. Frontal fontanelle, present at least at one stage during ontogeny (F2, <http://www.morphbank.net/Show/?id=461370>; Langston, 1960; Wagner, 2001): absent (0); present (1).
3. Frontal. Nasal articulation surface of the frontal shaped into a rostroventrally-sloping platform (F3, <http://www.morphbank.net/Show/?id=461371>; Godefroit et al., 2004, character 7, in part; Evans and Reisz, 2007, character 40, in part): absent (0); present (1).
4. Frontal. Nasal articulation surface of the frontal shaped into a dorsoventrally thickened, tongue-like platform that projects caudodorsally to overhang the parietal in adults (F4, <http://www.morphbank.net/Show/?id=461372>; Godefroit et al., 2004, character 7, in part; Evans and Reisz, 2007, character 40, in part, and character 41): absent (0); present (1).
5. Frontal. Median cleft separating the two striated tongues of the frontal platform (F5, <http://www.morphbank.net/Show/?id=461373>; Evans and Reisz, 2007, character 40, in part): absent (0); present (1).
6. Frontal. Triangular rostrolateral projection ending into narrow apex (F10; new character): absent (0); present (1).
7. Frontal. Exposure of the frontal at the dorsal margin of the orbit (F6; Horner et al., 2004, character 57): frontal completely excluded from the orbital margin by an extensive articulation between the prefrontal and postorbital (0); frontal exposed, forming part of the dorsal orbital margin in between the prefrontal and postorbital (1).
8. Frontal, upward doming dorsal to the braincase in subadult (and perhaps young adult) specimens (F7, <http://www.morphbank.net/Show/?id=461375>; modified after Horner et al., 2004, character 58): absent (0); present (1).
9. Frontal, length/width ratio of the ectocranial surface (F8, <http://www.morphbank.net/Show/?id=461376>; Evans and Reisz, 2007, character 42): relatively elongated ectocranial surface, with a ratio greater than 0.8 (0); relatively short ectocranial surface, with a ratio of 0.8 or less, but greater than 0.4 (1); greatly shortened ectocranial surface, with a ratio less than 0.4 (2).
10. Frontal. Morphology of the ventral annular ridge that defines the rostral extent of the cerebral fossa (F9, <http://www.morphbank.net/Show/?id=461377>; Evans and Resiz, 2007, character 43): long, low and gently rounded in medial view (0); sharp annular ridge (1).
11. Frontal platform extending caudodorsally to form a finger-shaped buttress under nasal crest (F11; Bell, 2011): absent (0); present (1).
12. Parietal. Maximum length/minimum width proportions of the adult parietal (PAR1, <http://www.morphbank.net/Show/?id=461378>; modified from Godefroit et al., 2004, character 18): very short, length/width ratio less than 1.40 (sample mean ratio of 1.19) (0); short, ratio between 1.40 and 2.35 (sample mean ratio of 1.98) (1); relatively long, ratio greater than 2.35 (sample mean ratio of 2.75) (2).
13. Parietal. Orientation of the parietal midline crest (PAR2, <http://www.morphbank.net/Show/?id=461380>; Horner et al., 2004; character 69; Evans and Resiz, 2007, character 44): straight and level with the skull roof or slightly down-warped along its length (0); the sagittal crest deepens caudally and is strongly down-warped (1).
14. Parietal. Rostral extension of the sagittal crest along the dorsal surface of the parietal (PAR4, <http://www.morphbank.net/Show/?id=461382>; Prieto-Márquez, 2010a, character 150): the sagittal crest extends along the entire length of the parietal and remains sharp and well defined at the rostral region, where it bifurcates along the craniomedial corner of the supratemporal fenestra (0); the sagittal crest extends along the entire length of the parietal but its sharpness fades away at the rostral region where the parietal is rostrocaudally shorter than it is wide (1); the sagittal crest only extends along the caudal half of the parietal and the rostral half of the dorsal surface of the parietal is flattened, lacking any ridge or mediolateral compression (2).
15. Basioccipital. Participation of the basioccipital in the ventral margin of the foramen magnum (BO1, <http://www.morphbank.net/Show/?id=461383>; Weishampel et al., 1993, character 24): absent, the exoccipital condyloids nearly or completely exclude the basioccipital from the ventral margin of the foramen magnum (0); present, the exoccipitals are separated at the sagittal plane of the braincase and allow the basioccipital to become part of the ventral margin of the foramen magnum (1).
16. Basioccipital. Length of basioccipital constriction (BO3, <http://www.morphbank.net/Show/?id=461385>; Godefroit, Zan and Jin, 2001): relatively long and well-developed (0); poorly developed and relatively short constriction (1).
17. Basisphenoid. Developement of the alar process of the basisphenoid (BS2, <http://www.morphbank.net/Show/?id=461387>; Prieto-Márquez, 2010a, character 155): moderately developed (0); very well developed, relatively large in size (1).
18. Basisphenoid. Ventral transverse caudal ridge between the basipterygoid processes of the basisphenoid (BS3, <http://www.morphbank.net/Show/?id=461388>; Gates and Sampson, 2007, character 78): absent or very poorly developed (0); present, sharply defined ridge (1).
19. Basisphenoid. Short median ventral process located between the basipterygoid processes of the basisphenoid (BS4, <http://www.morphbank.net/Show/?id=461389>; Gates and Sampson, 2007, character 79): absent (0); present, ventrally or rostroventrally inclined (1).
20. Basisphenoid. Development of the rostral constriction of the basisphenoid, caudal to the basipterygoid processes (measured as the ratio between the minimum mediolateral width of the rostral constriction and the maximum width of the basisphenoid across the spheno-occipital tubercles) (BS5, <http://www.morphbank.net/Show/?id=471320>; Prieto-Márquez, 2010a, character 158): very thick constriction, ratio less than 1.45 (sample mean ratio of 1.37) (0); moderately developed constriction, ratio between 1.45 and 1.90 (sample mean ratio of 1.72) (1); very thin constriction, ratio greater than 1.90 (sample mean ratio of 2.25) (2).
21. Laterosphenoid. Complete lateral osseous closure of the ophthalmic sulcus (V_1_) of the laterosphenoid (LS1, <http://www.morphbank.net/Show/?id=461391>; Evans and Reisz, 2007, character 51): absent (0); present (1).
22. Laterosphenoid. Extreme reduction of the length of the postorbital process of the laterosphenoid to 25% or less the length of the mediodorsal flange of this element (LS2, <http://www.morphbank.net/Show/?id=461392>; modified from Prieto-Márquez et al., 2006, character 76): absent (0); present (1).
23. Exoccipital. Caudal extension of the exoccipital-supraoccipital shelf above the foramen magnum (EX1, <http://www.morphbank.net/Show/?id=461395>; modified from Godefroit et al., 2004, character 24): very short rostrocaudal length, approximately less than half the diameter of the foramen magnum (0); moderately long, approximately more than half but less than the diameter of the foramen magnum (1); very long, substantially longer (often twice or more) than the diameter of the foramen magnum (2).
24. Pterygoid. Elevation of the proximodorsal region of the quadrate wing of the pterygoid (PLT1, <http://www.morphbank.net/Show/?id=461397>; Prieto-Márquez et al., 2006, character 72): absent (0); present (1).
25. Pterygoid. Ventral extension of the lamina located ventral to the central buttress of the pterygoid (PLT2, <http://www.morphbank.net/Show/?id=461398>; Prieto-Márquez et al., 2006, character 75): lamina of moderate size, a relatively large portion of the ventral quadrate process and the rostroventral process extends beyond the ventral margin of the lamina (0); extensive lamina, only a relatively small portion of the ventral quadrate process and the rostroventral process extends beyond the ventral margin of the lamina (1).
26. Ectopterygoid–jugal contact (PLT3, <http://www.morphbank.net/Show/?id=461399>; Godefroit et al., 2001, character 12): present, the ectopteryoid contacts the medial side of the jugal (0); absent, the jugal lacks an articular facet for the ectopterygoid (1).

Other Cranial Characters

1. Angle between the dorsal margin of the rostrum parallel to the long axis of the external naris and the maxillary tooth row (adults only) (RST2, <http://www.morphbank.net/Show/?id=461401>; Prieto-Márquez, 2010a, character 168): angle up to 30º (sample mean angle of 27º) (0); angle greater than 30º and up to 40º (sample mean angle of 34º) (1); angle greater than 40º (sample mean angle of 47º) (2).
2. Exposure of the nasal passage (NPS1, <http://www.morphbank.net/Show/?id=461402>; modified from Norman, 2002, character 5): present, nasal passage open and exposed on the lateral side of the rostrum (0); absent, nasal passage nearly or completely enclosed by bone and formation of internal cavities and passages (such as lateral diverticula and a common median chamber) (1). Distinction is made here between narial foramen (Wagner, 2001) and bony naris (Evans, 2006). Functionally, both structures formed the external naris. The iguanodontoidean narial foramen was regarded as homologous to the common median chamber of lambeosaurines (Hopson, 1975; Wagner, 2004). Because of caudodorsal migration of the external passage to become enclosed in a supracranial crest forming the common median chamber in lambeosaurines, what remained on the laterodorsal surface of the premaxilla (the bony naris) in the latter group was not regarded as structurally homologous to the narial foramen.
3. Lateral profile of the narial foramen (NPS2, <http://www.morphbank.net/Show/?id=461403> and <http://www.morphbank.net/Show/?id=461404>; Prieto-Márquez, 2010a, character 170): broad and subellipsoidal in lateral profile (0); narrow and subellipsoidal in lateral profile (1); extremely narrow, slit-like in lateral profile (2).
4. Degree of closure of the nasal passage on the lateral crest surface between the caudoventral process of the premaxilla and the nasal (NPS3, <http://www.morphbank.net/Show/?id=461405>; Evans, 2007, character 15): present, premaxilla-nasal fontanellae persist into late ontogenetic stages (0); absent, fontanellae completely closed in adults (1).
5. Ratio between the length of the narial foramen and the distance between the rostroventral corner of the premaxilla and the rostroventral margin of the prefrontal (NPS4, <http://www.morphbank.net/Show/?id=461406>; Prieto-Márquez, 2010a, character 172): very short narial foramen, ratio up to 0.40 (sample mean ratio of 0.32) (0); moderately long narial foramen, ratio greater than 0.40 but less than 0.60 (sample mean ratio of 0.49) (1); elongated narial foramen, ratio between 0.60 and 0.65 (sample mean ratio of 0.62) (2); extremely long narial foramen, ratio greater than 0.65 (sample mean ratio of 0.72) (3).
6. Nasal vestibule folded into an S-loop in the enclosed premaxillary passages rostral to the dorsal process of the maxilla (Weishampel, 1981; Evans, 2007, character 8): absent (Weishampel, 1981, Fig. 6) (0); present (Weishampel, 1981, Figs. 4-5) (1).
7. Location of the lateral diverticulum relative to the common median chamber (Weishampel, 1981): lateral to the common median chamber (Weishampel, 1981, Fig. 4 and 6) (0); rostral to the common median chamber (Weishampel, 1981, Fig. 10) (1); caudodorsal to the common median chamber (Weishampel, 1981, Fig. 8) (2).
8. Communication between the external bony naris, the lateral diverticulum and the common median chamber (Evans, 2006): a tubular premaxillary passage extends caudodorsally from the bony naris to the lateral diverticulum, that is then connected to the common median chamber (Weishampel, 1981, Figs. 4-6) (0); a tubular premaxillary passage connects directly the bony naris to the common median chamber (Weishampel, 1981, Fig. 7-8) (1).
9. Caudal extent of the nasal passage dorsal and/or caudal to the orbit (NPS8, <http://www.morphbank.net/Show/?id=461407>; modified from Evans, 2007, character 9): absent, nasal passage restricted to the antorbital region of the skull (0); present, but not extending caudal to the occiput, with a nasal vestibule that flanks a common median chamber (1); present, nasal vestibule extending caudodorsal to the occipital region of the skull (2).
10. Composition of the caudal margin of the functional external naris (NPS9, <http://www.morphbank.net/Show/?id=461408>; modified from Horner et al., 2004, character 29): formed by the nasal dorsally and the premaxilla ventrally (0); formed entirely by the nasal (1); formed entirely by the premaxilla (2).
11. Circumnarial fossa on the lateral surface of the facial region of the skull (CMN1, <http://www.morphbank.net/Show/?id=461409>; Wagner, 2001; Horner et al., 2004, character 31): absent, circumnarial structure entirely enclosed (0); present (1).
12. Caudodorsal extension of circumnarial fossa (homologous to the lateral diverticulum inside hollow supracranial crests) (CMN2, <http://www.morphbank.net/Show/?id=461411> and <http://www.morphbank.net/Show/?id=461412>; Hopson, 1975; Wagner, 2001; 2004; modified from Horner et al., 2004, characters 32): the fossa does not reach the caudal margin of the narial foramen and, thus, lacks a caudal margin (0); the fossa extends as far as to surround the caudal margin of the narial foramen, but does not reach the orbit (1); the fossa extends as far as the rostrodorsal region of the orbit (2); the fossa extends beyond the orbit, caudodorsal to its caudal margin (3).
13. Degree of excavation of the caudal region of the circumnarial fossa (CMN3, <http://www.morphbank.net/Show/?id=461410>; Horner et al., 2004, character 32): lightly incised (0); deeply incised (1); invaginated (2).
14. Elevation of the skull roof dorsal to the ancestral lateral profile (i.e., presence of supracranial crest) (CRS1, <http://www.morphbank.net/Show/?id=461413>; Wagner, 2004): absent (0); present (1).
15. Composition of the supracranial crest (or the homologous region of the skull from which the crest forms) (excluding supporting elements) (CRS2, <http://www.morphbank.net/Show/?id=461414>; modified from Horner et al., 2004, character 40): composed exclusively of the nasals (0); primarily composed of the nasals and frontals (1); primarily composed of the nasals and premaxillae (2).
16. Relative contribution of the nasal and premaxilla in the formation of hollow supracranial crests (CRS3, <http://www.morphbank.net/Show/?id=461415>; Wagner, 2001 and modified in part from Evans and Reisz, 2007, character 11): the nasals constitute half or a larger portion of the crest in the form of a caudal plate-like surface (0); the nasals form a smaller portion of the crest relative to the surrounding premaxillae (1).
17. Supracranial crest shape (CRS4, <http://www.morphbank.net/Show/?id=461416>, <http://www.morphbank.net/Show/?id=461417>, <http://www.morphbank.net/Show/?id=461418>, and <http://www.morphbank.net/Show/?id=461419>; modified from Horner et al., 2004, character 36): dome-like broad and low protuberance (0); mediolaterally compressed arcuate protuberance, rostral or, in adults, dorsal to the level to the orbits (1); paddle-like and caudally (as well as slightly dorsally) directed solid blade of bone (2); mediolaterally narrow and paddle-like, extending caudal to the occiput (3); rostrally excavated and rostrally-facing protuberance, rostrodorsal to the orbit (4); nasal fold that rises dorsally or caudodorsally to form a laterally excavated promontory, with a caudal region that rests over the frontals (5); raised into a large vertical fan, formed by a solid plate-like extension of the premaxilla (‘cockscomb’) above the nasal passages in the rostral region of the crest (6); long and tubular, caudodorsally directed beyond the occiput and slightly arched (7).
18. Hollow crest-snout angle along the dorsal margin of the premaxilla in lateral view (in adults) (CRS5, <http://www.morphbank.net/Show/?id=461420>; Evans, 2007, character 13): absent, the lateral profile of the snout is continuous with the lateral profile of the dorsal premaxillary margin of the crest (0); facial profile shallowly concave in lateral view, angle greater than 140º (1); angle between 110º and 140º (2); crest procumbent and rostrally inclined, angle less than 110º (3).
19. Caudal extension of the hook-like nasal process on the caudoventral region of helmet-shaped hollow supracranial crests (CRS6, <http://www.morphbank.net/Show/?id=461421>; Prieto-Márquez, 2010a, character 186): rostral to or at the level of the caudal margin of the occiput (0); extended caudal to the level of the caudal margin of the occiput (1).
20. Palpebral (supraorbital) bone (PLP, <http://www.morphbank.net/Show/?id=461422>; Norman, 2002, character 13): absent (0); present (1).
21. Length/width proportions of the orbit (ORB, <http://www.morphbank.net/Show/?id=461424>; Wagner, 2001): nearly circular, approximately as wide as it is deep (0); elongated, dorsoventrally deeper than it is wide (1).
22. Presence of a gap (paraquadratic foramen) between the quadratojugal and the jugal (PQF, <http://www.morphbank.net/Show/?id=461425>; Prieto-Márquez, 2010a, character 189): absent (0); present (1).
23. Size of the infratemporal fenestra relative to that of the orbit (ITF1, <http://www.morphbank.net/Show/?id=461426>; modified from Gates and Sampson, 2007): infratemporal fenestra both rostrocaudally wider and dorsoventrally deeper than the orbit (0); infratemporal fenestra rostrocaudally narrower than or approximately as wide as the orbit (1).
24. Shape and rostrocaudal width of the dorsal margin of the infratemporal fenestra relative to that of the dorsal orbital margin (ITF2, <http://www.morphbank.net/Show/?id=461427> and <http://www.morphbank.net/Show/?id=461428>; Prieto-Márquez, 2010a, character 191): subrectangular, with a dorsal infratemporal margin that is approximately as wide as the ventral margin (0); subtriangular, with a dorsal infratemporal margin that is narrower than the ventral margin (1).
25. Location of the dorsal margin of the infratemporal fenestra relative to the dorsal margin of the orbit (ITF3, <http://www.morphbank.net/Show/?id=461564>; modified from Gates and Sampson, 2007, character 86): the dorsal margin of the infratemporal fenestra lies approximately at the same level than the dorsal margin of the orbit and the caudal region of the skull roof is subhorizontal or slightly sloping rostroventrally relative to the frontal plane (0); the dorsal margin of the infratemporal fenestra is substantially more dorsally located than the dorsal margin of the orbit and the caudal region of the skull roof is rostroventrally inclined relative to the frontal plane (1); the dorsal margin of the infratemporal fenestra lies slightly or substantially below the level of the dorsal margin of the orbit and the caudal region of the skull roof is subhorizontal or slightly sloping caudoventrally relative to the frontal plane (2).
26. Morphology of the dorsal outline of the supratemporal fenestra (STF, <http://www.morphbank.net/Show/?id=461565>; Prieto-Márquez, 2010a, character 193): subrectangular, with the long axis directed rostrally (0); oval, with the long axis directed rostrolaterally (1); oval and wider mediolaterally than rostrocaudally (2).
27. Depth of the skull (ratio between the skull height along caudal margin of quadrate and distance from rostral predentary tip to the level of the caudal margin of quadrate) (SK1; modified from You et al., 2003, character 1): ratio less than 0.70 (0); relatively deep skull, ratio of 0.70 or greater (1).
28. Maximum transverse width of the cranium in dorsal view across the postorbitals relative to the width across the quadrate cotylus of the squamosals (SK2, <http://www.morphbank.net/Show/?id=461699>; modified from Horner et al., 2004, character 67): the skull is more than 25% wider across the postorbitals (sample mean ratio of 0.35) (0); the skull is up to 25% wider across the postorbitals (sample mean ratio of 0.14) (1).

Vertebral Characters

1. Cervical vertebrae. Morphology of the dorsal flange of the axis (CRV1, <http://www.morphbank.net/Show/?id=461701>; modified from Campione, Evans and Cuthbertson, 2007): dorsally convex flange extending beyond or to the level of the cranialmost region of the postzygapophyses (0); presence of shor­­t cranial flange separated from the postzygapophyseal region by a prominent embayment (1).
2. Cervical vertebrae. Development of the postzygapophyseal proceses of cranial and middle cervical vertebrae (CRV2, <http://www.morphbank.net/Show/?id=461702>; modified from Horner et al., 2004, character 74): relatively low and relatively short, less than three times the rostrocaudal breadth of the neural arch (0); relatively high and relatively long, three times or more longer than the breadth of the neural arch (1).
3. Number of cervical vertebrae (Horner et al., 2004, character 72): 11 or less (0); 12 or more (1).
4. Height of the neural spine relative to that of the centrum of the tallest caudal dorsal or sacral vertebrae (in adults) (DRS1, <http://www.morphbank.net/Show/?id=461703>; modified from Norman, 2002, character 41): short neural spine, ratio up to 2.10 (mean ratio of 1.79) (0); ratio greater than 2.10 and up to 3.25 (mean ratio of 2.57) (1); very long neural spine, ratio greater than 3.25 (mean ratio of 3.97) (2).
5. Slightly elongated neural spines in the cranial dorsal vertebrae, forming a ‘wither-like’ region above the pectoral girdle (DRS2, <http://www.morphbank.net/Show/?id=461704>; Wagner, 2001): absent (0); present (1).
6. Minimum count of co-ossified vertebrae in the sacral region (including single dorsal and caudal contributions (Godefroit et al., 2000, character 27): seven or fewer (0); eight or more (1).
7. Chevron length relative to the length of the neural spines in the caudal vertebrae of the proximal half of the tail (CDL, <http://www.morphbank.net/Show/?id=461705>; Wagner, 2001): chevrons shorter or nearly as long as the neural spines (0); chevrons longer than the neural spines (1).

Pectoral and Forelimb Characters

1. Sternum. Length of the ‘handle-like’ caudolateral process of the sternal relative to that of the rostromedial plate (excluding the caudoventral process) (ST, <http://www.morphbank.net/Show/?id=461706>; modified from Prieto-Márquez et al., 2006, character 100): caudolateral process slightly shorter or as long as the rostromedial plate (0); caudolateral process longer than the rostromedial plate (1).
2. Coracoid size relative to the length of the scapula (COR1, <http://www.morphbank.net/Show/?id=461707>; Horner et al., 2004, character 77): relatively large coracoid, ratio between rostrocaudal length of coracoid and length of scapula of approximately 0.2 (0); coracoid reduced in size relative to the scapula (1).
3. Coracoid. Ratio between the length of the lateral margin of the facet for the scapular articulation and the length of the lateral margin of the glenoid (COR2, <http://www.morphbank.net/Show/?id=461708>; Prieto-Márquez, 2010a, character 206): longer scapular facet, with a ratio greater than 1.30 (sample mean ratio of 1.48) (0); slightly longer scapular facet, ratio greater than 1 and up to 1.30 (sample mean ratio of 1.14) (1); glenoid longer than the scapular facet, with a ratio up to 1 (sample mean ratio of 0.75) (2).
4. Coracoid. Angle between the lateral margins of the facet for scapular articulation and the glenoid (COR3, <http://www.morphbank.net/Show/?id=461709>; Prieto-Márquez, 2010a, character 207): angle greater than 115º (sample mean angle of 124º) (0); angle up to 115º (sample mean angle of 102º) (1).
5. Coracoid. Morphology of the rostromedial margin of the coracoid (COR4, <http://www.morphbank.net/Show/?id=461710>; Horner et al., 2004, character 78): convex or straight, associated to a moderate development and slight projection biceps tubercle (0); concave, associated to a relatively large and lateroventrally-projected biceps tubercle (1).
6. Coracoid. Development of the ‘hook-like’ ventral process of the coracoid, measured as the ratio between the dorsoventral depth and the breadth of the process (COR5, <http://www.morphbank.net/Show/?id=461711>; modified from Godefroit et al., 2000, character 25): relatively short, ratio less than 0.65 (sample mean ratio of 0.55) (0); ratio between 0.65 to 0.80 (sample mean ratio of 0.71) (1); long process, nearly as deep as it is wide, with a ratio greater than 0.80 (sample mean ratio of 0.96) (2).
7. Coracoid. Curvature of the ventral ‘hook-like’ process of the coracoid (COR6, <http://www.morphbank.net/Show/?id=461712>; Godefroit et al., 2000, character 25): ventrally directed (0); recurved, so that the process is caudoventrally directed (1).
8. Scapula. Lateral profile of the dorsal margin of the scapula (SCP1, <http://www.morphbank.net/Show/?id=461713>; modified from Sereno, 1986): rostrocaudally straight from the cranial margin of the coracoid facet to the distal end of the blade (0); curved, dorsally convex, curvature originating at the level of the dorsal margin of the pseudoacromion process and is most pronounced over the dorsoventral constriction (1).
9. Scapular length, ratio between the rostrocaudal length of the scapula (from the cranial end of the pseudoacromion process to the distal margin of the blade) and the dorsoventral depth of the cranial end (from the cranial end of the pseudoacromion process to the ventral apex of the glenoidal facet) (SCP2, <http://www.morphbank.net/Show/?id=461714>; Prieto-Márquez, 2010a, character 212): relatively short scapula, ratio up to 4 (sample mean ratio of 3.54) (0); relatively long scapula, ratio greater than 4 (sample mean ratio of 4.64) (1).
10. Dorsoventral expansion of the distal region of the scapular blade (measured as a ratio between the depth of the distal end of the blade and the depth of the proximal region) (SCP4, <http://www.morphbank.net/Show/?id=461716>; Prieto-Márquez, 2010a, character 213): ratio less than 1 (sample mean ratio of 0.80) (0); ratio of 1 or greater (sample mean ratio of 1.15) (1).
11. Scapula. Proximal constriction (scapular ‘neck’), ratio between the dorsoventral width of the proximal constriction and the dorsoventral depth of the cranial end of the scapula (SCP5, <http://www.morphbank.net/Show/?id=461717>; Prieto-Márquez, 2010a, character 214): narrow ‘neck’, ratio up to 0.60 (sample mean ratio of 0.53) (0); relatively broad ‘neck’, ratio greater than 0.60 (sample mean ratio of 0.68) (1).
12. Scapula. Morphology and orientation of the pseudoacromion process (SCP6, <http://www.morphbank.net/Show/?id=461718>; modified from Horner et al., 2004, character 80): recurved, so that the cranial region is dorsally or rostrodorsally directed (0); horizontal, occasionally with minor and subtle dorsal or ventral curvatures, so that the cranial region is rostrally or mostly rostrally directed (1).
13. Scapula. Degree of curvature of the dorsally oriented pseudoacromion process of the scapula (SCP7, <http://www.morphbank.net/Show/?id=461718>; modified from Horner et al., 2004, character 80): strongly recurved, so that the cranial region of the process is dorsally oriented (0); slightly recurved, with concave lateral profile of the dorsal margin, so that the cranial region of the process is rostrodorsally oriented (1).
14. Scapula. Cranial extension of the rostrodorsal region of the scapula (bearing the coracoid facet), measured as a ratio between the distance from the coracoid joint and the cranial end of the pseudoacromion process and the height between this and the ventral apex of the glenoidal facet (SCP8, <http://www.morphbank.net/Show/?id=461719>; Prieto-Márquez, 2010a, character 217): short rostrodorsal region, ratio less than 0.45 (sample mean ratio of 0.35) (0); long rostrodorsal region, ratio of 0.45 or greater (sample mean ratio of 0.53) (1).
15. Scapula. Development of the deltoid ridge (SCP9, <http://www.morphbank.net/Show/?id=461720>; Prieto-Márquez, 2010a, character 218): dorsoventrally narrow convexity limited to the proximal region of the scapula, near the pseudoacromion process from which it develops, with a poorly demarcated ventral margin (0); dorsoventrally deep and rostrocaudally long, with a well demarcated ventral margin (1).
16. Humerus. Length of the deltopectoral crest of the humerus (measured as the ratio between the proximodistal length of the crest and the proximodistal length of the humerus (HM1, <http://www.morphbank.net/Show/?id=461721>; modified from Godefroit et al., 2000, character 26): proximodistally short crest, ratio less than 0.48 (sample mean ratio of 0.44) (0); ratio between 0.48 and 0.55 (sample mean ratio of 0.52) (1); very long crest, ratio greater than 0.55 (sample mean ratio of 0.59) (2).
17. Humerus. Lateroventral expansion of the deltopectoral crest of the humerus (measured as the ratio between the width of the humerus across the distal fourth of the deltopectoral crest and the width of the distal shaft at the point of maximum curvature) (HM2, <http://www.morphbank.net/Show/?id=461722>; modified from Horner et al., 2004): poorly expanded deltopectoral crest, ratio less than 1.65 (sample mean ratio of 1.53) (0); ratio between 1.65 and 1.90 (sample mean ratio of 1.76) (1); very expanded deltopectoral crest, ratio greater than 1.90 (sample mean ratio of 2) (2).
18. Humerus. Degree of angulation of the ventral margin of the deltopectoral crest (HM3, <http://www.morphbank.net/Show/?id=471338>; Weishampel et al., 1993, character 37): well-rounded (0); extending abruptly from the humeral shaft to give a distinct angular profile (1).
19. Humerus. Overall proportions of the humerus (measured as a ratio between the total length and the width of the lateral surface of the proximal end of the humerus) (HM4, <http://www.morphbank.net/Show/?id=461724>; modified from Weishampel et al. 1993, character 36): relatively short and stocky humerus, ratio less than 4.25 (mean ratio of 3.85) (0); ratio between 4.25 and 4.90 (sample mean ratio of 4.60) (1); relatively long and thin humerus, ratio greater than 4.90 (mean ratio of 5.4) (2).
20. Ulna. Length of the ulna relative to its dorsoventral thickness (measured at mid-shaft) (UL1, <http://www.morphbank.net/Show/?id=461725>; Prieto-Márquez, 2010a, character 223): ratio length/width less than 10 (0); ratio length/width equal or larger than 10 (1).
21. Ulnar length relative to humeral length (UL2, <http://www.morphbank.net/Show/?id=461726>; Norman, 2002, character 47): ulna shorter than or as long as the humerus (0); longer ulna, up to 20% longer than the humerus (1); longer ulna, being more than 20% longer than the humerus (2).
22. Composition of the carpus (MN1, <http://www.morphbank.net/Show/?id=461727>; adapted from Horner et al., 2004, character 86): presence of fused ulnare, radiale, intermedium and distal carpals (0); number of carpal bones reduced to a maximum of two unfused elements (1).
23. Manual digit I (Norman, 2002, character 49) (MN2, <http://www.morphbank.net/Show/?id=461728>): presence of metacarpal I and one ungual phalanx (0); entire digit I absent (1).
24. Elongation of the manus exemplified by elongation of metacarpals II through IV, measured as the ratio between the length of metacarpal III and the width of its mid-shaft (MN3, <http://www.morphbank.net/Show/?id=461729>; modified from Horner et al., 2004, character 84): relatively short and blocky, ratio up to 5 (sample mean ratio of 4.25) (0); relatively long and slender, ratio greater than 5 (sample mean ratio of 8.54) (1).
25. Elongation of metacarpal V, so that it is more than twice as long as it is proximally wide (MN4, <http://www.morphbank.net/Show/?id=461730>; Prieto-Márquez, 2010a, character 228): absent (0); present (1).
26. Length/width proportions of manual phalanx III1 (MN6, <http://www.morphbank.net/Show/?id=461732>; modified from Prieto-Márquez et al., 2006, character 114): proximodistally compressed, mediolaterally wider than it is long (0); slightly longer proximodistally than it is wide mediolaterally (1); very elongated, proximodistal length that is at least twice its mediolateral width at the middle of its longitudinal axis (2).
27. Shape of manual ungual II (MN7, <http://www.morphbank.net/Show/?id=461733>; Norman, 2002, character 53, in part): claw-like (0); hoof-like (1).
28. Proximodistal length of manual palanx II1 relative to that of II2 (MN8, <http://www.morphbank.net/Show/?id=461734>; modified from You et al., 2003, character 55): phalanx II1 less than three times longer than phalanx II2 (0); phalanx II1 three times or more longer than phalanx II2 (1).

Pelvic Characters

1. Ilium. Angle of ventral deflection of the preacetabular process of the ilium (IL1, <http://www.morphbank.net/Show/?id=461735>; modified from Suzuki et al., 2004, character 69): angle greater than 150º (sample mean angle of 162º) (0); angle of 150º or less (sample mean angle of 143º) (1).
2. Ilium. Dorsoventral depth of the proximal region of the preacetabular process (measured as a ratio between this and the dorsoventral distance between the pubic peduncle and the dorsal margin of the ilium) (IL3, <http://www.morphbank.net/Show/?id=461737>; Prieto-Márquez, 2010a, character 233): shallow, less than half the depth of the cranial central plate, ratio less than 0.50 (sample mean ratio of 0.42) (0); approximately as deep as the cranial central plate depth, ratio between 0.50 and 0.55 (sample mean ratio of 0.51) (1); deeper than half the depth of the cranial central plate, ratio greater than 0.55 (sample mean ratio of 0.62) (2).
3. Ilium. Dorsoventral depth of the central plate of the ilium (expressed as a ratio between this and the distance between the pubic peduncle and the caudodorsal prominence of the ischial peduncle) (IL4, <http://www.morphbank.net/Show/?id=461738>; Prieto-Márquez, 2010a, character 234): ratio of 0.80 or greater (sample mean ratio of 0.90) (0); ratio less than 0.80 (sample mean ratio of 0.71) (1).
4. Ilium. Position of the ventral-most margin of the supraacetabular process relative to the caudoventral margin of the lateral ridge of caudal protuberance of the ischial peduncle of the ilium (IL5, <http://www.morphbank.net/Show/?id=461739>; Brett-Surman and Wagner, 2007): apex located caudodorsally (0); apex located above caudoventral margin of protuberance (1); apex located rostrodorsally (2).
5. Ilium. Development of the lateroventral projection of the supraacetabular process of the ilium (IL6, <http://www.morphbank.net/Show/?id=461740>; modified from Horner et al., 2004, character 91): forms a longitudinal and continuous ‘swelling’ or reflected border along the dorsal margin of the central plate and the proximal region of the postacetabular process, with a depth up to 25% the depth of the ilium (0); projected lateroventrally at least 25% (but less than half) the depth of the ilium (1); projects lateroventrally between half and three quarters of the dorsoventral depth of the ilium (2); projects lateroventrally to overlap totally or at least half of the lateral ridge of the caudal prominence of the ischial peduncle (3).
6. Ilium. Rostrocaudal breadth of the supraacetabular process, measured as the ratio between the breadth of the process across its dorsal region and the rostrocaudal length of the central iliac blade from the caudal ischial peduncle to the pubic one (IL7, <http://www.morphbank.net/Show/?id=461741>; Prieto-Márquez, 2010a, character 237): rostrocaudally wider than the central plate of the ilium, ratio greater than 0.85 (sample mean ratio of 1.16) (0); rostrocaudally broad, ratio between 0.70 and 0.85 (sample mean of 0.73) (1); slightly broader than half the length of the central iliac blade, ratio between 0.55 and 0.69 (sample mean ratio of 0.62) (2); short, ratio less than 0.55 (sample mean ratio of 0.48) (3).
7. Ilium. Symmetry of the lateral profile of the supraacetabular process (IL8, <http://www.morphbank.net/Show/?id=461742>; Prieto-Márquez, 2010a, character 238): asymmetrical, with a caudally skewed lateral profile (0); symmetrical or with a slightly caudally skewed profile (1).
8. Ilium. Morphology of the lateroventral margin of the supraacetabular process (IL9, <http://www.morphbank.net/Show/?id=461743>; Prieto-Márquez, 2010a, character 239): rostrocaudally sinuous (0); widely arched (1); U or V-shaped (2); subrectangular, with a shallow notch that divides the ventral margin in two poorly demarcated lobes (3).
9. Ilium. Demarcation of the caudodorsal margin of the lateroventral rim of the supraacetabular process (IL10, Prieto-Márquez, 2010a, character 240): the caudodorsal margin is poorly defined and appears discontinuous with the dorsal margin of the proximal region of the postacetabular process due to the lack of a well demarcated caudodorsal ridge (<http://www.morphbank.net/Show/?id=461744>) (0); the caudodorsal margin is a well-defined ridge that is continuous with the dorsal margin of the proximal region of the postacetabular process (<http://www.morphbank.net/Show/?id=461799>) (1).
10. Ilium. Morphology of the pubic peduncle of the ilium (IL11, <http://www.morphbank.net/Show/?id=461745> and <http://www.morphbank.net/Show/?id=461746>; modified from Sereno, 1986; Horner et al., 2004; character 92): relatively large and dorsoventrally deep (longer than wide), subconical, with a proximal region that is only slightly rostrocaudally wider than the distal end of the process (0); relatively shorter (wider or as wide as long) and triangular, with a proximal region that is much rostrocaudally wider than the distal end (1).
11. Ilium. Morphology of the ischial peduncle of the ilium (IL12, <http://www.morphbank.net/Show/?id=461747> and <http://www.morphbank.net/Show/?id=461748>; modified from Godefroit et al., 2001, character 30): formed by a single and large, oval ventral protrusion (0); composed of a large and oval ventral protrusion and by a smaller, caudodorsally located prominence emerging from the caudodorsal ridge (1); formed by two protrusions of similar size, the caudal-most one located slightly caudodorsally (2).
12. Ilium. Ratio between the rostrocaudal length of the postacetabular process and the rostrocaudal length of the central plate of the ilium (IL13, <http://www.morphbank.net/Show/?id=461749>; Prieto-Márquez, 2010a, character 243): short postacetabular process, ratio up to 0.80 (sample mean ratio of 0.70) (0); postacetabular process nearly as long as the central plate, ratio greater than 0.80 but less than 1.1 (sample mean ratio of 0.90) (1); postacetabular process substantially longer than the central plate, ratio of 1.1 or greater (sample mean ratio of 1.23) (2).
13. Ilium. Brevis shelf at the base of the postacetabular process of the ilium (IL14, <http://www.morphbank.net/Show/?id=461750>; modified in part from Horner et al., 2004, character 93): absent (0); present (1).
14. Ilium. Medioventral ridge on the medial side of the postacetabular process, crossing the bone surface from the proximoventral to the caudodorsal margins and orientation of the brevis shelf (IL15, <http://www.morphbank.net/Show/?id=468875>; Prieto-Márquez, 2010a, character 245): absent or presence of a faint ridge (0); well-defined ridge bounding the medial margin of the brevis shelf; the latter faces medioventrally and in medial view appears restricted to the caudal region of the postacetabular process (1); well-defined ridge forming the medial margin of a medioventrally-facing shelf, with a postacetabular process that is progressively expanded mediolaterally towards the caudal end (2); well-developed, oblique and expanded flange forming the medial margin of an extensive brevis shelf that faces more ventrally than medially (3).
15. Ilium. Rostrocaudally-oriented median ridge on the laterodorsal surface of the postacetabular process (IL16, <http://www.morphbank.net/Show/?id=461752>; Prieto-Márquez, 2010a, character 246): absent (0); present (1).
16. Ilium. Geometry of the lateral profile of the postacetabular process of the ilium (IL17, <http://www.morphbank.net/Show/?id=461753> and <http://www.morphbank.net/Show/?id=461754>; modified from Horner et al., 2004, character 93): the ventral margin converges caudodorsally to meet the horizontal dorsal margin, forming a tapering caudal end and producing a triangular lateral profile of the process (0); dorsal and ventral margins parallel or slightly convergent, forming a distinct (rectangular or subcircular) caudal margin (1).
17. Ilium. Orientation of the dorsal margin of the postacetabular process relative to the acetabular margin (IL18, <http://www.morphbank.net/Show/?id=461755>; Prieto-Márquez, 2010a, character 248): horizontal dorsal margin, parallel or nearly parallel to the acetabular margin (0); caudodorsally oriented dorsal margin, rising dorsally relative to the acetabular margin (1).
18. Ilium. Position of the medial sacral ridge within the medial surface of the central plate of the ilium (IL19, <http://www.morphbank.net/Show/?id=461756>; Prieto-Márquez, 2010a, character 249): ridge well separated ventrally from the dorsal margin of the ilium, set between 50% and 30% the dorsoventral depth of the central plate (0); ridge located more dorsally, closer to the dorsal margin, within the dorsal third (less than 30% the depth) of the central plate (1).
19. Ilium. Lateral profile of the dorsal or laterodorsal margin of the ilium (IL21, <http://www.morphbank.net/Show/?id=461758>; modified from Horner et al., 2004, character 100): straight or slightly convex (0); distinctly depressed over the supraacetabular process and dorsally bowed over the proximal region of the preacetabular process (1).
20. Pubis. Orientation of the dorsoventral expansion of the prepubic process relative to the longitudinal axis of the proximal constriction of the process (PB1, <http://www.morphbank.net/Show/?id=461759>; Prieto-Márquez, 2010a, character 252): the dorsal region of the expansion is more expanded than the ventral region, so that distally the process is dorsally-directed (0); the ventral region is more expanded than the dorsal region, so that the distal expansion is ventrally-directed (1).
21. Pubis. Geometry of the dorsoventral expansion of the prepubic process of the pubis (in lateral or medial views) (PB2, <http://www.morphbank.net/Show/?id=461760>, <http://www.morphbank.net/Show/?id=461761>, and <http://www.morphbank.net/Show/?id=461762>; modified in part from Wagner, 2001): circular to oval expansion, extensive and convex ventral margin (0); subsquared distal dorsal margin, expansion dorsoventrally taller than rostroventrally long, very pronounced proximal dorsal concavity and nearly straight distal ventral margin (1); ellipsoidal, expansion rostrocaudally longer than dorsoventrally tall, well pronounced concavities of the dorsal and ventral proximal margins (2); oval expansion, dorsoventrally taller than rostrocaudally long, well pronounced concave profiles of dorsal and ventral proximal margins (3); rectangular, rostrocaudally longer than dorsoventrally tall, nearly straight profiles of the dorsal and ventral proximal margins (4).
22. Pubis. Depth of the dorsoventral expansion of the distal region of the prepubic process relative to the width of the acetabular margin of the pubis (PB3, <http://www.morphbank.net/Show/?id=461763>; Prieto-Márquez, 2010a, character 254): distal expansion as wide as or shallower than width of the acetabular margin (0); dorsoventral expansion deeper than the acetabular margin (1).
23. Pubis. Rostrocaudal length of the proximal constriction of the prepubic process of the pubis relative to length of the dorsoventral expansion (PB4, <http://www.morphbank.net/Show/?id=461764>; modified from Horner et al., 2004, character 96): constriction longer than the dorsoventral expansion, that is restricted to the distal region of the process (0); constriction and distal expansion have approximately the same length (1); constriction slightly shorter than the dorsoventral expansion, that begins at the proximal region of the process (2).
24. Pubis. Relative position of maximum concavity of the dorsal and ventral margins of the prepubic process of the pubis (PB5, <http://www.morphbank.net/Show/?id=461765>; Prieto-Márquez, 2010a, character 256): maximum ventral concavity achieved adjacent to the proximal region of the postpubic process, maximum dorsal concavity located further distally (0); maximum ventral concavity located ventral to or slightly caudal to the maximum dorsal concavity (1).
25. Pubis. Morphology of the acetabular margin, ventral to the lateral edge of the iliac peduncle (PB6, <http://www.morphbank.net/Show/?id=461766>; Prieto-Márquez, 2010a, character 257): the lateral margin of the iliac peduncle extends ventrally forming a prominent ridge that merges with the proximal region of the ischial peduncle (0); the lateral margin of the iliac peduncle progressively disappears ventrally into the lateral surface of the region adjacent to the acetabular margin (1).
26. Pubis, obturator foramen (PB7, <http://www.morphbank.net/Show/?id=461767>; modified from Horner et al., 2004, character 97): present, proximal postpubic ramus has a caudodorsally oriented short process that contacts totally or partially with the ischial peduncle to form the obturator foramen (0); absent, proximal postpubic ramus lacks a dorsocaudally oriented process (1).
27. Pubis. Length/width ratio of the ischial peduncle of the pubis (PB8, <http://www.morphbank.net/Show/?id=461768>; Prieto-Márquez, 2010a, character 259): very short ischial peduncle, ratio less than 1.85 (sample mean ratio of 1.5) (0); ratio ranging from 1.85 to less than 3 (sample mean ratio of 2.4) (1); very long, ratio of 3 or more (with a sample mean ratio of 4) (2).
28. Pubis. Lateroventral protuberance on the proximal region of the ischial peduncle of the pubis (PB9, <http://www.morphbank.net/Show/?id=461769>; Prieto-Márquez, 2010a, character 260): absent or very faintly developed (0); present (1).
29. Pubis. Depth/width proportions of the iliac peduncle of the pubis (PB10, <http://www.morphbank.net/Show/?id=461770>; Prieto-Márquez, 2010a, character 261): rostrocudally broader than dorsoventrally tall (0); taller dorsoventrally than broad rostrocaudally (1).
30. Pubis. Total length of the pubis, as the ratio between the rostrocaudal distance from the acetabular margin to the distal margin of the prepubic process and the distance from the dorsal margin of the iliac peduncle and the ventral margin of the proximal postpubic shaft (PB11, <http://www.morphbank.net/Show/?id=471324>; Prieto-Márquez, 2010a, character 262): short, ratio less than 2.70 (sample mean raio of 2.30) (0); long, ratio between 2.70 and 3 (sample mean ratio of 2.84) (1); very long, ratio greater than 3 (sample mean ratio of 3.53) (2).
31. Ischium. Development of a caudal curvature of the distal margin of the iliac peduncle of the ischium (IS1, <http://www.morphbank.net/Show/?id=461772> and <http://www.morphbank.net/Show/?id=461773>; Brett-Surman and Wagner, 2007): curvature absent, distal margin slightly rounded and, in some exemplars, slightly curved rostrally (0); presence of a very short and slight curvature in the caudodorsal corner (1); presence of a well developed curvature in the caudodorsal corner, so that the peduncle appears ‘thumb-like’ in lateral and medial profiles (2).
32. Ischium. Elongation of the iliac peduncle of the ischium (ratio between the proximodistal length and the rostrocaudal width of the distal margin) (IS3, <http://www.morphbank.net/Show/?id=461775>; Prieto-Márquez, 2010a, character 265): relatively short peduncle, ratio less than 1.5 (sample mean ratio of 1.27) (0); ratio between 1.5 and 2 (sample mean ratio of 1.78) (1); relatively long peduncle, ratio greater than 2 (sample mean ratio of 2.30) (2).
33. Ischium. Relative orientation of the acetabular and caudodorsal margins of the iliac peduncle of the ischium (IS4, <http://www.morphbank.net/Show/?id=461776>; Prieto-Márquez, 2010a, character 266: margins are either parallel or slightly convergent relative to each other (correlated with a greater expansion of the rostrodorsal corner of the peduncle) (0); margins become slightly to greatly divergent near the proximal region of the peduncle (1).
34. Ischium. Orientation of the rostrocaudal axis of the pubic peduncle (perpendicular to its articular margin) relative to the ischial shaft (IS5, <http://www.morphbank.net/Show/?id=461777>; Prieto-Márquez, 2010a, character 267): ventrally inclined, angle up to 130º (sample mean angle of 118º) (0); slightly inclined ventrally, angle greater than 130 and up to 170º (sample mean angle of 157º) (1); pubic peduncle parallel to the ischial shaft (2).
35. Ischium. Length/width proportions of the pubic peduncle of the ischium (IS6, <http://www.morphbank.net/Show/?id=461778>; Prieto-Márquez, 2010a, character 268): proximodistally longer than the distal articular surface is wide (0); approximately as long proximodistally as the distal articular surface is dorsoventrally wide (1); proximodistally shorter than the dorsoventral width of the distal articular surface (2).
36. Ischium. Relative position of the dorsal acetabular margin of the pubic peduncle (IS7, <http://www.morphbank.net/Show/?id=461779>; Prieto-Márquez, 2010a, character 269): ventral to or at the same level as the dorsal margin of the ischial shaft (0); peduncular margin set dorsal to the dorsal margin of the ischial shaft (1).
37. Ischium. Dorsoventral thickness of the mid-shaft of the ischium (measured as a ratio between this and the length of the entire shaft) (IS8, <http://www.morphbank.net/Show/?id=461780>; modified from Wagner, 2001): very thin shaft, up to 5% the length of the ischial shaft (sample mean of 4.6%)(0); thickness ranging from more than 5% and up to 7.5% the length of the ischial shaft (sample mean of 6.7%) (1); very thick shaft, thickness greater than 7.5% the length of the ischial shaft (sample mean of 8.4%) (2).
38. Ischium. Morphology of the distal region of the ischial shaft (IS9, <http://www.morphbank.net/Show/?id=461781> and <http://www.morphbank.net/Show/?id=462510>; modified from Godefroit et al., 2001, character 31): slightly expanded into a blunt end (0); ventrally expanded forming a large ‘foot’ or ‘boot-like’ process (1).
39. Ischium. Degree of ventral projection of the distal expansion of the ischium (expressed as the ratio between the length of the ischial shaft and the length of the distal ventral expansion) (IS10, <http://www.morphbank.net/Show/?id=461783>; Evans and Reisz, 2007, character 90): ratio less than 0.25 (sample mean ratio of 0.18) (0); ratio of 0.25 or greater (sample mean ratio of 0.28) (1).
40. Ischium. Morphology of the cranial margin of the ventral expansion of the distal ischial shaft (IS11, <http://www.morphbank.net/Show/?id=461784>; Prieto-Márquez, 2010a, character 273): slightly concave and directed caudoventrally to meet the caudal margin to nearly a point (0); strongly concave, recurved cranial margin (1).
41. Ischium. Orientation of the long axis of the distal ‘foot’ relative to the ischial shaft (IS12, <http://www.morphbank.net/Show/?id=461785>; Prieto-Márquez, 2010a, character 274): straight, ventrally directed (0); rostroventrally directed, the inclination starting at the dorsal margin of the ‘foot’ (1).

Hindlimb Characters

1. Femur. Degree of curvature of the distal half of the femoral shaft (FM1, (<http://www.morphbank.net/Show/?id=461786>; Norman, 2002, character 62): slightly curved caudomedially (0); absence of curvature, straight distal shaft (1).
2. Femur. Lateral profile of the caudoventral margin of the fourth trochanter of the femur (FM2, [http://www.morphbank.net/Show/?id=461787;](http://www.morphbank.net/Show/?id=461787) modified from Wagner, 2001): triangular and ending in a caudally, and slightly ventrally, directed point (0); smooth and arcuate (1).
3. Tibia. Extension of the cnemial crest of the tibia (TB, <http://www.morphbank.net/Show/?id=461788>; Godefroit et al., 2000, character 31): the cnemial crest is restricted to the proximal end of the tibia (0); cnemial crest further extended along the cranial surface of the proximal half of the diaphysis (1).
4. Astragalus. Development of the medial platform of the astragalus (AS, <http://www.morphbank.net/Show/?id=461790>; Prieto-Márquez, 2010a, character 279): it extends medially to completely underlie the medial malleolus of the tibia (0); short, wedges laterally underlying only part of the medial malleolus of the tibia (1).
5. Distal tarsals II and III (Horner et al., 2004, character 102): present (0); absent (1).
6. Metatarsal I (modified from Norman, 2002, character 66): absent (0); slender and splint-like element (1).
7. Length/width proportions of pedal phalanx II2 (PES3, <http://www.morphbank.net/Show/?id=461792>; Prieto-Márquez, 2010a, character 283): proximodistally shortened, being twice as wide mediolaterally as it is proximodistally long (0); subsquared, only slightly shorter proximodistally than it is wide mediolaterally (1).
8. Length/width proportions of the disc-shaped pedal phalanx III2-III3 (PES4, <http://www.morphbank.net/Show/?id=461793>; modified from Horner et al., 2004, character 104): up to three times (or less) wider than they are proximodistally long (0); more than three times wider than they are proximodistally long (1).
9. Morphology of the pedal unguals (PES6, [http://www.morphbank.net/Show/?id=461795;](http://www.morphbank.net/Show/?id=461035)

Norman, 2002, character 67): proximodistally elongated and arrow-shaped, with a bluntly truncated tip and prominent claw grooves (0); mediolaterally broad and proximodistally shortened, rounded shield or hoof-like shaped, with reduced or absent claw grooves (1).

1. Ridge on the plantar surface of pedal unguals (PES7, <http://www.morphbank.net/Show/?id=461796>; Prieto-Márquez, 2005, character 47): absent (0); present (1).

Literature Cited

Bell PR (2011) Redescription of the skull of *Saurolophus osborni* Brown 1912 (Ornithischia: Hadrosauridae). Cretaceous Research 32: 30–44.

Brett-Surman MK, Wagner JR (2007) Discussion of character analysis of the appendicular anatomy in Campanian and Maastrichtian North American hadrosaurids—variation and ontogeny. In: Carpenter K, ed. Horns and Beaks. Ceratopsian and Ornithopod Dinosaurs. Bloomington: Indiana University Press, 135–169.

Campione NE, Evams DC (2011) Cranial growth and variation in edmontosaurs (Dinosauria: Hadrosauridae): implications for latest Cretaceous megaherbivore diversity in North America. PLoS ONE 6(9): e25186. doi:10.1371/journal.pone.0025186.

Campione N, Evans DC, Cuthbertson R (2007) Anatomy of the atlas-axis complex of hadrosaurid dinosaurs. Journal of Vertebrate Paleontology 27: 55A.

Evans DC. 2006. Nasal cavity homologies and cranial crest function in lambeosaurine dinosaurs. Paleobiology 32:109–125.

Evans DC (2007) Ontogeny and evolution of lambeosaurine dinosaurs (Ornithischia: Hadrosauridae). PhD dissertation, University of Toronto, Toronto, Ontario, 497 pp.

Evans DC, Reisz RR (2007) Anatomy and relationships of *Lambeosaurus magnicristatus*, a crested hadrosaurid dinosaur (Ornithischia) from the Dinosaur Park Formation, Alberta. Journal of Vertebrate Paleontology 27: 373–393.

Gates TA, Sampson SD (2007) A new species of *Gryposaurus* (Dinosauria: Hadrosauridae) from the late Campanian Kaiparowits Formation, southern Utah, USA. Zoological Journal of the Linnean Society 151: 351–376.

Godefroit P, Zan S, Jin L (2000) *Charonosaurus* *jiayinensis* n.g., n.sp., a lambeosaurine dinosaur from the Late Cretaceous of northeastern China. Comptes Rendus de l’Academie des Sciences de Paris, Sciences de la Terre et des Planetes 330: 875–882.

Godefroit P, Zan S, Jin L (2001) The Maastrichtian (Late Cretaceous) lambeosaurine dinosaur *Charonosaurus jiayinensis* from north-eastern China. Bulletin de l’Institut Royal des Sciences Naturelles du Belgique 71: 119–168.

Godefroit P, Alifanov V, Bolotsky Y (2004) A re-appraisal of *Aralosaurus* *tuberiferus* (Dinosauria, Hadrosauridae) from the Late Cretaceous of Kazakhstan. Bulletin de l’Institut Royal des Sciences Naturelles du Belgique 74: 139–154.

Godefroit P, Shulin H, Tingxiang Y, Lauters P (2008) New hadrosaurid dinosaurs from the uppermost Cretaceous of northeastern China. Acta Palaeontologica Polonica 53: 47–74.

Hopson JA (1975) The evolution of cranial display structures in hadrosaurian dinosaurs. Paleobiology 1: 21–43.

Horner JR, Weishampel DB, Forster CA (2004) Hadrosauridae. In: Weishampel DB, Dodson P, Osmólska H, eds. The Dinosauria, Second Edition. Berkeley: University of California Press, 438–463.

Kobayashi Y, Azuma Y (2003) A new Iguanodontian (Dinosauria: Ornithopoda) from the lower Cretaceous Kitadani Formation of Fukui Prefecture, Japan. Journal of Vertebrate Paleontology 23: 166–175.

Langston WD (1960) The vertebrate fauna of the Selma Formation of Alabama. Part 6: the dinosaurs. Fieldiana: Geology Memoirs 3: 313–363.

Norman DB (2002) On Asian ornithopods (Dinosauria: Ornithischia). 4. *Probactrosaurus* Rozhdestvensky, 1966. Zoological Journal of the Linnean Society 136: 113–144.

Prieto-Márquez A (2005) New information on the cranium of *Brachylophosaurus canadensis* (Dinosauria, Hadrosauridae), with a revision of its phylogenetic position. Journal of Vertebrate Paleontology 25: 144–156.

Prieto-Márquez A (2010a) Global phylogeny of Hadrosauridae (Dinosauria: Ornithopoda) using parsimony and Bayesian methods. Zoological Journal of the Linnean Society 159: 435–502.

Prieto-Márquez A (2010b) *Glishades ericksoni*, a new hadrosauroid (Dinosauria: Ornithopoda) from the Late Cretaceous of North America. Zootaxa 2452: 1–17.

Prieto-Márquez A, Wagner JR (In press) *Saurolophus* *morrisi*, a new species of hadrosaurid dinosaur from the Late Cretaceous of the Pacific coast of North America. Acta Palaeontologica Polonica doi: 10.4202/app.2011.0049.

Prieto-Márquez A, Gaete R, Rivas G, Galobart A, Boada M (2006) Hadrosauroid dinosaurs from Western Europe: *Pararhabdodon isonensis* revisited and *Koutalisaurus kohlerorum* n. gen. et sp. Journal of Vertebrate Paleontology 26: 929–943.

Sereno PC (1986) Phylogeny of the bird-hipped dinosaurs (Order Ornithischia). National Geographic Research 2: 234–256.

Suzuki D, Weishampel DB, Minoura N (2004) *Nipponosaurus sachalinensis* (Dinosauria: Ornithopoda): anatomy and systematic position within Hadrosauridae. Journal of Vertebrate Paleontology 24: 145–164.

Wagner JR (2001) The hadrosaurian dinosaurs (Ornithischia: Hadrosauria) of Big Bend National Park, Brewster County, Texas, with implications for Late Cretaceous Paleozoogeography. 417 pp. Unpublished Master’s Thesis, Texas Tech University, Austin.

Wagner JR (2004) Hard-tissue homologies and their consequences for interpretation of the cranial crests of lambeosaurine dinosaurs (Dinosauria: Hadrosauria). Journal of Vertebrate Paleontology 24: 125A–126A.

Weishampel DB (1981) The nasal cavity of lambeosaurine hadrosaurids (Reptilia: Ornithischia): comparative anatomy and homologies. Journal of Paleontology 55: 1046–1057.

Weishampel DB, Norman DB, Grigorescu D (1993) *Telmatosaurus* *transsylvanicus* from the Late Cretaceous of Romania: the most basal hadrosaurid. Palaeontology 36: 361–385.

You HL, Luo Z, Shubin NH, Witmer LM, Tang ZL, Tang F (2003) The earliest-known duck-billed dinosaur from deposits of late Early Cretaceous age in northwestern China and hadrosaur evolution. Cretaceous Research 24: 347–355.
