# Supplementary material for: Diversity, Relationships, and Biogeography of the Lambeosaurine Dinosaurs from the European Archipelago, with Description of the New Aralosaurin Canardia garonnensis
Source: PLoS One. 2013 Jul 26;8(7):e69835. doi: 10.1371/journal.pone.0069835 (PMC3724916; doi:10.1371/journal.pone.0069835)
Supplement: Information S3 — Results of the Bayesian-Binary MCMC analysis [105] performed on each of the five most parsimonious trees resulting from maximum parsimony analysis of lambeosaurine relationships. Numbers represent probability proportions of inferred ancestral areas. Node numbers correspond to those in the phylograms included below. (PDF) [file pone.0069835.s003.pdf]

Supporting Information S3

Results of the Bayesian-Binary MCMC analysis performed on each of the five most parsimonious trees resulting from maximum parsimony analysis of lambeosaurine relationships. Numbers represent probability proportions of inferred ancestral areas. Node numbers correspond to those in the phylograms included below.

Areas

A = North America; B = European Archipelago; C = Asia

Tree 1

Node 29: C 87.19 BC 6.90 B 3.20 AC 1.71 A 0.79 ABC 0.14 AB 0.06

Node 30: C 86.21 BC 6.43 B 4.78 AC 1.38 A 1.03 ABC 0.10 AB 0.08

Node 31: A 99.85 AC 0.09 AB 0.06 C 0.00 B 0.00 ABC 0.00 BC 0.00

Node 32: A 98.13 AC 1.56 C 0.25 AB 0.05 B 0.01 ABC 0.00 BC 0.00

Node 33: A 44.49 C 37.99 AC 17.04 B 0.24 AB 0.11 BC 0.09 ABC 0.04

Node 34: A 99.84 AC 0.10 AB 0.06 C 0.00 B 0.00 ABC 0.00 BC 0.00

Node 35: A 99.93 AC 0.04 AB 0.03 C 0.00 B 0.00 ABC 0.00 BC 0.00

Node 36: B 88.75 AB 9.04 A 2.15 BC 0.04 C 0.01 ABC 0.00 AC 0.00

Node 37: A 95.98 AB 3.38 B 0.46 AC 0.15 C 0.02 ABC 0.01 BC 0.00

Node 38: C 92.56 AC 5.89 A 1.44 BC 0.08 B 0.02 ABC 0.01 AB 0.00

Node 39: A 93.85 AC 5.07 C 0.95 AB 0.11 B 0.02 ABC 0.01 BC 0.00

Node 40: A 97.87 AC 1.86 C 0.19 AB 0.08 B 0.01 ABC 0.00 BC 0.00

Node 41: A 91.58 AC 6.78 C 1.40 AB 0.18 B 0.04 ABC 0.01 BC 0.00

Node 42: A 98.55 AC 0.83 AB 0.53 C 0.05 B 0.03 ABC 0.00 BC 0.00

Node 43: A 99.72 AC 0.17 AB 0.10 C 0.00 B 0.00 ABC 0.00 BC 0.00

Node 44: A 99.74 AC 0.18 AB 0.08 C 0.00 B 0.00 ABC 0.00 BC 0.00

Node 45: A 97.29 AC 2.34 C 0.26 AB 0.09 B 0.01 ABC 0.00 BC 0.00

Node 46: A 38.53 C 36.08 AC 25.02 B 0.13 AB 0.09 BC 0.09 ABC 0.06

Node 47: C 89.83 AC 5.90 A 2.19 BC 1.42 B 0.53 ABC 0.09 AB 0.03

Node 48: C 95.72 AC 2.79 BC 0.95 A 0.38 B 0.13 ABC 0.03 AB 0.00

Node 49: C 87.43 AC 9.14 A 1.62 BC 1.39 B 0.25 ABC 0.15 AB 0.03

Tree 2 (only probabilities for the *Hypacrosaurus stebingeri-Amurosaurus riabinini* clade)

Node 36: B 90.69 AB 7.62 A 1.62 BC 0.06 C 0.01 ABC 0.00 AC 0.00

Node 37: A 90.35 AB 6.18 B 2.80 AC 0.43 C 0.19 ABC 0.03 BC 0.01

Node 38: A 91.05 AC 4.72 C 2.44 AB 1.12 B 0.58 ABC 0.06 BC 0.03

Node 39: C 91.96 AC 6.43 A 1.52 BC 0.07 B 0.02 ABC 0.00 AB 0.00

Node 40: A 96.40 AC 3.12 C 0.36 AB 0.10 B 0.01 ABC 0.00 BC 0.00

Node 41: A 99.43 AC 0.49 AB 0.06 C 0.02 B 0.00 ABC 0.00 BC 0.00

Node 42: A 98.77 AC 1.00 AB 0.17 C 0.05 B 0.01 ABC 0.00 BC 0.00

Node 43: A 99.77 AC 0.17 AB 0.06 C 0.00 B 0.00 ABC 0.00 BC 0.00

Tree 3 (only probabilities for the *Hypacrosaurus stebingeri-Amurosaurus riabinini* clade)

Node 36: B 87.84 AB 9.59 A 2.52 BC 0.04 C 0.01 ABC 0.00 AC 0.00

Node 37: A 95.87 AB 3.44 B 0.51 AC 0.14 C 0.02 ABC 0.01 BC 0.00

Node 38: C 92.35 AC 6.08 A 1.47 BC 0.07 B 0.02 ABC 0.00 AB 0.00

Node 39: A 96.16 AC 3.29 C 0.43 AB 0.11 B 0.01 ABC 0.00 BC 0.00

Node 40: A 99.39 AC 0.50 AB 0.08 C 0.02 B 0.00 ABC 0.00 BC 0.00

Node 41: A 98.85 AB 0.55 AC 0.51 B 0.05 C 0.04 ABC 0.00 BC 0.00

Node 42: A 95.18 AC 3.78 C 0.74 AB 0.24 B 0.05 ABC 0.01 BC 0.00

Node 43: A 99.31 AC 0.61 AB 0.07 C 0.01 B 0.00 ABC 0.00 BC 0.00

Tree 4 (only probabilities for the *Hypacrosaurus stebingeri-Amurosaurus riabinini* clade)

Node 36: B 97.82 AB 1.72 BC 0.23 A 0.20 C 0.03 ABC 0.00 AC 0.00

Node 37: A 48.75 C 20.39 B 19.07 AC 4.87 AB 4.56 BC 1.91 ABC 0.46

Node 38: A 98.40 AC 0.73 AB 0.69 C 0.09 B 0.08 ABC 0.01 BC 0.00

Node 39: C 91.21 AC 6.99 A 1.70 BC 0.07 B 0.02 ABC 0.01 AB 0.00

Node 40: A 96.48 AC 3.10 C 0.32 AB 0.10 B 0.01 ABC 0.00 BC 0.00

Node 41: A 99.59 AC 0.35 AB 0.05 C 0.01 B 0.00 ABC 0.00 BC 0.00

Node 42: A 99.62 AC 0.25 AB 0.13 C 0.00 B 0.00 ABC 0.00 BC 0.00

Node 43: A 99.88 AC 0.07 AB 0.05 C 0.00 B 0.00 ABC 0.00 BC 0.00

Tree 5 (only probabilities for the *Hypacrosaurus stebingeri-Amurosaurus riabinini* clade)

Node 36: A 88.97 AC 4.86 C 4.26 AB 0.96 B 0.84 ABC 0.05 BC 0.05

Node 37: B 90.99 AB 7.37 A 1.55 BC 0.07 C 0.01 ABC 0.01 AC 0.00

Node 38: A 91.53 AB 4.78 B 2.14 AC 1.01 C 0.45 ABC 0.05 BC 0.02

Node 39: C 92.09 AC 6.32 A 1.50 BC 0.07 B 0.02 ABC 0.00 AB 0.00

Node 40: A 96.42 AC 3.09 C 0.37 AB 0.10 B 0.01 ABC 0.00 BC 0.00

Node 41: A 99.53 AC 0.37 AB 0.08 C 0.01 B 0.00 ABC 0.00 BC 0.00

Node 42: A 99.02 AB 0.59 AC 0.34 B 0.03 C 0.02 ABC 0.00 BC 0.00

Node 43: A 99.79 AB 0.12 AC 0.09 B 0.00 C 0.00 ABC 0.00 BC 0.00

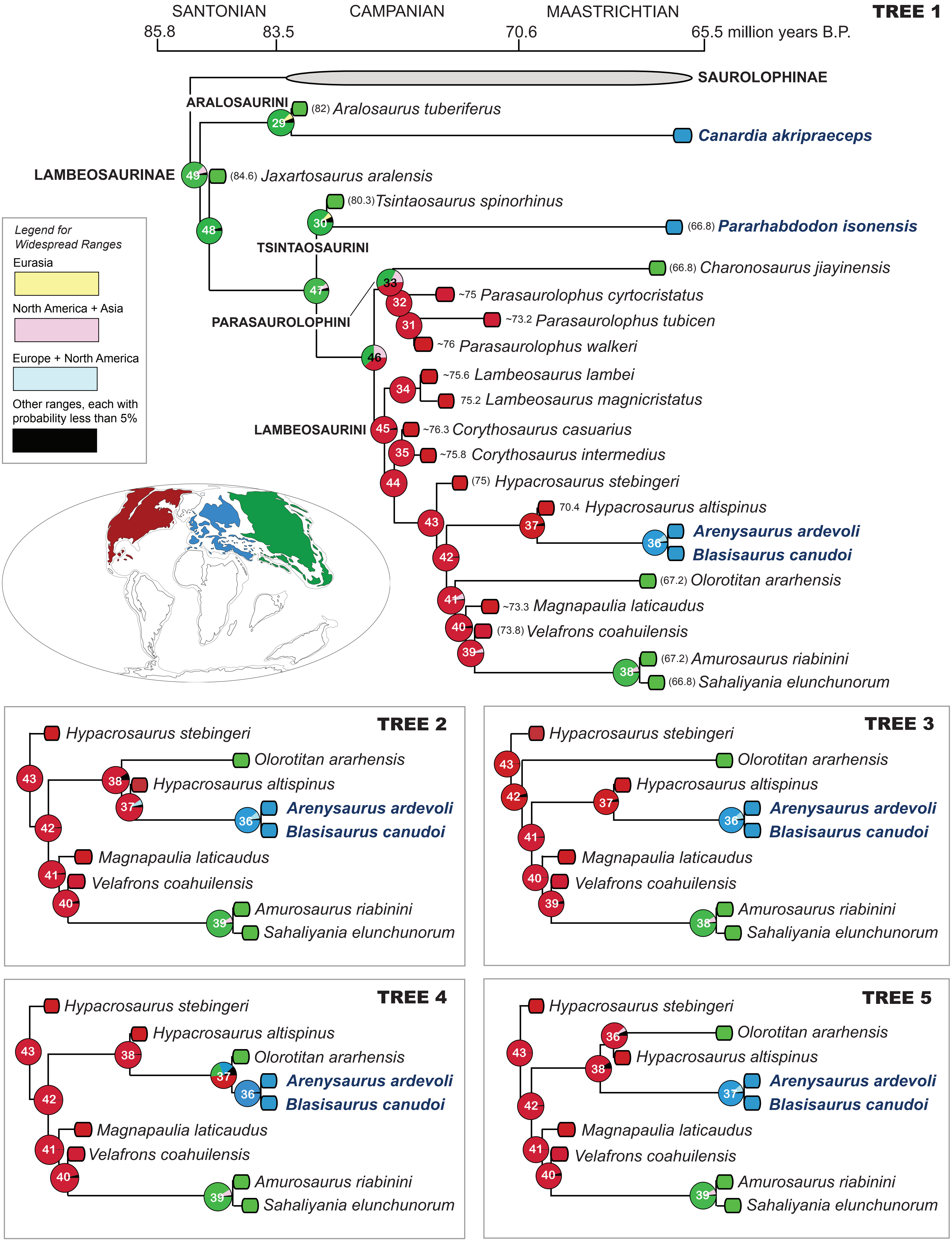
TREE 1
SANTONIAN
CAMPANIAN
MAASTRICHTIAN
85.8
83.5
70.6
65.5 million years B.P.
SAUROLOPHINAE
ARALOSAURINI
(82) Aralosaurus tuberiferus
Canardia akripraeceps
LAMBEOSAURINAE
(84.6) Jaxartosaurus aralensis
(80.3) Tsintaosaurus spinorhinus
(66.8) Pararhabdodon isonensis
TSINTAOSAURINI
(66.8) Charonosaurus jiayinensis
~75 Parasaurolophus cyrtocristatus
PARASAUROLOPHINI
~73.2 Parasaurolophus tubicen
~76 Parasaurolophus walkeri
~75.6 Lambeosaurus lambei
75.2 Lambeosaurus magnicristatus
LAMBEOSAURINI
~76.3 Corythosaurus casuarius
~75.8 Corythosaurus intermedius
(75) Hypacrosaurus stebingeri
70.4 Hypacrosaurus altispinus
Arenysaurus ardevoli
Blasisaurus canudoi
(67.2) Olorotitan ararhensis
~73.3 Magnapaulia laticaudus
(73.8) Velafrons coahuilensis
(67.2) Amurosaurus riabinini
(66.8) Sahaliyania elunchunorum
Legend for Widespread Ranges
Eurasia
North America + Asia
Europe + North America
Other ranges, each with probability less than 5%
TREE 2
Hypacrosaurus stebingeri
Olorotitan ararhensis
Hypacrosaurus altispinus
Arenysaurus ardevoli
Blasisaurus canudoi
Magnapaulia laticaudus
Velafrons coahuilensis
Amurosaurus riabinini
Sahaliyania elunchunorum
TREE 3
Hypacrosaurus stebingeri
Olorotitan ararhensis
Hypacrosaurus altispinus
Arenysaurus ardevoli
Blasisaurus canudoi
Magnapaulia laticaudus
Velafrons coahuilensis
Amurosaurus riabinini
Sahaliyania elunchunorum
TREE 4
Hypacrosaurus stebingeri
Hypacrosaurus altispinus
Olorotitan ararhensis
Arenysaurus ardevoli
Blasisaurus canudoi
Magnapaulia laticaudus
Velafrons coahuilensis
Amurosaurus riabinini
Sahaliyania elunchunorum
TREE 5
Hypacrosaurus stebingeri
Olorotitan ararhensis
Hypacrosaurus altispinus
Arenysaurus ardevoli
Blasisaurus canudoi
Magnapaulia laticaudus
Velafrons coahuilensis
Amurosaurus riabinini
Sahaliyania elunchunorum
